# Alpha-Synuclein Seed Amplification Assays in Parkinson’s Disease: A Systematic Review and Network Meta-Analysis

**DOI:** 10.3390/clinpract15060107

**Published:** 2025-06-03

**Authors:** Jamir Pitton Rissardo, Ana Leticia Fornari Caprara

**Affiliations:** Neurology Department, Cooper University Hospital, Camden, NJ 08103, USA; fornari-caprara-ana@cooperhealth.edu

**Keywords:** alpha-synuclein, synucleinopathy, biomarker, biofluid, protein misfolding cyclic amplification (PMCA), real-time quaking-induced conversion (RT-QuIC)

## Abstract

Introduction and objective: Assessment of α-synuclein (αSyn) seed amplification assays (αSyn-SAA) accuracy in distinguishing Parkinson’s disease (PD) from controls using cerebrospinal fluid (CSF), blood, skin, extracellular vesicles (ECV), saliva, olfactory mucosa (OM), gastrointestinal tract (GIT), and submandibular gland (SMG). Methodology: PubMed was searched for articles from 2010 to January 2025. The quality assessment used robvis. Diagnostic values with a 95% confidence interval (CI) were obtained. Z-test, Wald CI, and ANOVA were performed. Diagnostic odds ratio (DOR) was used. Results: αSyn-SAAs showed strong diagnostic performance in distinguishing PD from controls across various tissue and fluid types. Overall, αSyn-SAAs demonstrated high sensitivity (86%) and specificity (92%). Among all biomatrices, CSF, skin, blood, and ECV yielded the highest diagnostic accuracy, with sensitivity and specificity approaching or exceeding 90%. In contrast, saliva, oral mucosa, and gastrointestinal tract samples showed more modest sensitivity, though specificity remained relatively high. ECV, CSF, skin, and blood matrices also demonstrated the highest DOR, supporting their potential clinical utility. Conclusions: ECV and blood warrant priority in αSyn-SAA for high accuracy and minimal invasiveness, while GIT, OM, and oral samples show limited utility; saliva and SMG need refinement.

## 1. Introduction

α-Synuclein (αSyn) is a presynaptic neuronal protein implicated in the pathogenesis of Parkinson’s disease (PD) and related synucleinopathies, including dementia with Lewy bodies (DLB). Its aggregation into Lewy bodies and Lewy neurites is a hallmark of these disorders, contributing to neuronal dysfunction and degeneration [1]. Significant efforts have been made in terms of the scientific and international patient organization communities to utilize αSyn as a biomarker for early diagnosis and monitoring disease progression in PD. In this context, several methods were created to quantify αSyn levels in biological fluids. Immunoassays, such as enzyme-linked immunosorbent assays (ELISAs), are commonly used to measure total αSyn concentrations in cerebrospinal fluid (CSF) and blood plasma [2]. However, these assays may not distinguish between monomeric and aggregated forms. To address this, techniques like real-time quaking-induced conversion (RT-QuIC) have been employed to detect misfolded αSyn aggregates with high sensitivity and specificity [3]. Immunoprecipitation followed by mass spectrometry (IP-MS) has also been utilized to analyze specific αSyn species, providing detailed information on post-translational modifications and aggregation states [4]. In this way, the current study aims to systematically review the literature regarding the different biomatrices for the seed amplification assay of αSyn and the diagnostic results for PD. We also examine the biomatrices used for αSyn quantification and highlight key features of specific tests employed to measure its levels. The current review differs from previous systematic studies in the literature by incorporating a greater number of matrices, including extracellular vesicles, and by employing diverse comparison models that emphasize specificity among matrices.

## 2. Methodology

### 2.1. Literature Search Strategy

This meta-analysis followed the PRISMA statement [5]. Two reviewers (J.P.R. and A.L.F.C.) systematically searched the electronic PubMed database by the following search terms: alpha-synuclein, real-time quaking-induced conversion, protein misfolding cyclic amplification, cerebrospinal fluid, blood, skin, extracellular vesicles, saliva, olfactory mucosa, gastrointestinal tract, submandibular gland, and Parkinson’s disease (S1—Search Strategy). The protocol of this study was registered on PROSPERO (CRD420250150129).

### 2.2. Inclusion and Exclusion Criteria

The criteria of the published articles to be included in the current systematic review were (1) only manuscripts in the English language; (2) investigating αSyn seed amplification assays (αSyn-SAA) in PD with a control group without synucleinopathy; (3) all biomatrices or source material for the aggregation assay were included; (4) the detection method for αSyn aggregate was PMCA or RT-QuIC; and (5) studies directly or indirectly reporting the sensitivity and specificity outcomes were included.

Studies were excluded if they met any of the following criteria: (1) duplicate publications; (2) systematic reviews, meta-analyses, letters, protocols, case reports, case series, and conference abstracts; (3) studies lacking relevant data; and (4) research conducted on animals. Due to the absence of established standard cut-off values for αSyn-SAA, no predetermined thresholds for test positivity were applied.

### 2.3. Data Extraction

Two investigators (J.P.R. and A.L.F.C.) independently review the literature and extract data about the first author’s last name; year of publication; sample size, including patients with PD and controls; true positives (TP), false negatives (FN), true negatives (TN), and false positives (FP) number of individuals; processing sample; type of assay (PMCA and RT-QuIC); and cut-off values to be considered as positive samples.

### 2.4. Quality Assessment

The quality tool used to assess the methodological quality concerning the risk of bias and applicability was robvis^®^ (Risk-of-bias VISualization, https://www.riskofbias.info/welcome/robvis-visualization-tool, accessed on 22 April 2025) [6]. The data were displayed according to the Quality Assessment Tool for Diagnostic Accuracy Studies (QUADAS).

### 2.5. Statistical Analysis

The statistical analysis was performed in the groups comparing PD and controls (patients without synucleinopathy). The data were extracted and stored in an Excel spreadsheet (Microsoft Excel version 2023 for macOS). The mean data of the results were obtained from Excel.

The data were indirectly calculated with Diagnostic Test Evaluation Calculator version 23.1.5 (https://www.medcalc.org/calc/diagnostic_test.php, accessed on 22 April 2025) for those manuscripts that did not report the sensitivity, specificity, positive likelihood ratio, negative likelihood ratio, positive predictive value, negative predictive value, and accuracy with the 95% confidence interval (CI) [7].

Forest plots were created in GraphPad Prism version 10.4.1 for Mac, GraphPad Software, Boston, Massachusetts, USA (www.graphpad.com, accessed on 22 April 2025) [8]. For data synthesis, we utilized MetaDTA (https://crsu.shinyapps.io/MetaDTA/, accessed on 22 April 2025), an online tool that implements a random-effects bivariate model [9]. To summarize the sensitivity and specificity of each study, hierarchical summary receiver operating characteristic (HSROC) curves were generated, accompanied by 95% CI [10].

Two types of statistical analysis were performed to evaluate the overall sensitivity and specificity, called pooled and single-population analyses [11]. Pooled analysis was a common mean weighted according to the number of individuals in every study, represented in the figures as a transparent diamond (◇). Single-population analysis was based on the pooled diagnostic (TP, TN, FP, and FN) data and described as a unique population, represented in the figures as a black diamond (◆). Noteworthy, single-population analysis was never performed in the literature to evaluate αSyn-SAA in PD and likely represents a more reliable value than the pooled mean since there is a narrower standard deviation. The difference between sensitivities and specificities of the different biomatrices was statistically analyzed with a Z-Test Calculator for 2 Population Proportions from Social Science Statistics (https://www.socscistatistics.com/, accessed on 22 April 2025) [12]. This test compares the difference between two sample proportions (sensitivities or specificities) to determine if the difference is statistically significant. The significance level was *p*-value < 0.05, and the two-tailed hypothesis was selected. Another test performed was Wald CI for the difference, which was used to assess whether there is a statistically significant difference between two proportions (or means) by calculating the CI for their difference and checking whether it includes zero [13].

A network meta-analysis using the analysis of variance (ANOVA) model was conducted to assess the relative sensitivity and specificity of αSyn-SAA across various specimen types, providing a probabilistic ranking of their diagnostic performance with the diagnostic odds ratio (DOR) through the R software package rstan (version 2.32.6) [14]. In the network plot, each node represents a biomatrix; larger nodes indicate higher sensitivity values; edges (connections) simulate pairwise comparisons, weighted based on sample sizes. Heterogeneity was assessed using I^2^ statistics, with values exceeding 50% indicating significant variability [15].

Youden’s Index (J) was used to assess the overall diagnostic accuracy of each test, calculated as J = Sensitivity + Specificity − 1. A higher Youden’s Index indicates better discriminative ability [16]. Differences between tests were evaluated using a Z-test for Youden’s Index, with statistical significance set at *p*-value < 0.05. Receiver operating characteristic (ROC) curves were generated to compare the overall performance of the tests, and the area under the curve (AUC) was computed for each test. DeLong’s test was used to statistically compare AUC values, as it provides a non-parametric estimate of the variance and accounts for the correlation between ROC curves. A *p*-value < 0.05 in DeLong’s test indicated a significant difference in discriminatory performance between tests. The R software package pROC (version 1.18.5) was used [17].

ROC curves were generated to evaluate the diagnostic performance of each test. Since the available data consisted of discrete values (i.e., TP, FN, TN, and FP), a smoothing approach was applied to create a more realistic representation of diagnostic performance [18]. Simulated probability scores were assigned to generate a continuous ROC curve according to diagnostic confidence. TP were given probabilities sampled from a high-confidence range of 0.7 to 1.0, while FN and TN were assigned probabilities within a low-confidence range of 0.0 to 0.3. FP was assigned probabilities similar to true positives, ranging from 0.7 to 1.0, to account for diagnostic uncertainty. These estimated probability distributions allowed for constructing a smooth ROC curve, where the true positive rate (TPR, sensitivity) was plotted against the false positive rate (FPR, 1-specificity) across varying thresholds. The AUC was calculated for each test to compare overall diagnostic performance. All statistical analyses for developing the smoothing approach for ROC curve estimation were conducted using Python version 3.9.0 (https://www.python.org, accessed on 22 April 2025) with the scikit-learn package (v1.2.2) for macOS [19]. The scikit-learn package was employed for ROC curve estimation and AUC calculation, while GraphPad Prism was used for data visualization and additional statistical analyses. Final ROC curves were rendered in grayscale to enhance clarity and ensure a consistent visual presentation. A *p*-value of <0.05 was considered the threshold for statistical significance in all comparisons.

## 3. Results

### 3.1. Study Selection

**Figure 1:**
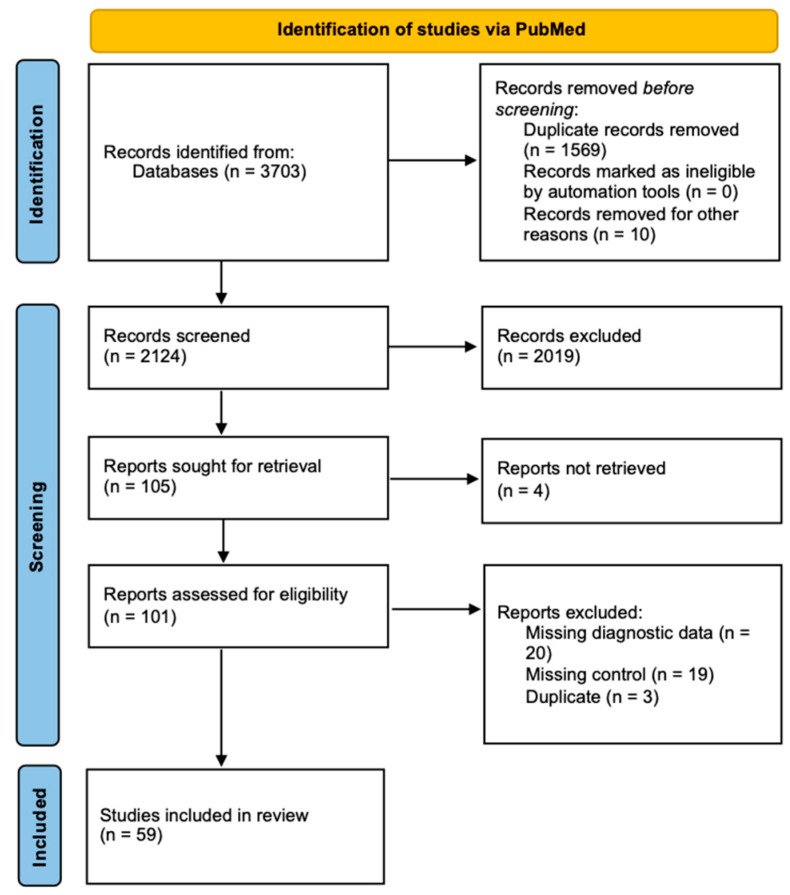
PRISMA flowchart for the identification of included studies.

An initial screening of PubMed yielded 3703 articles, of which 59 met the predefined inclusion criteria. A comprehensive summary of the study selection process is illustrated in the following Figure 1. A total of 70 analyses were performed across the 59 studies, with some studies contributing multiple biomatrix types or utilizing different assay techniques, leading to varied diagnostic results.

De Luca et al. and Shin et al. were only included for the analysis of sensitivity, but they were excluded from the specificity due to the absence of results regarding TN and FP [20,21]. Consequently, they also did not have a likelihood ratio, predictive values, and accuracy. All the other case diagnostic data were described, and the data were extrapolated.

All the studies compared patients with PD and patients with non-neurodegenerative neurological conditions. Most control cases were healthy individuals, except for Sakurai et al., who evaluated individuals with normal pressure hydrocephalus with and without PD [22].

### 3.2. Study Characteristics

The clinical criteria for diagnosing PD varied across the studies: twenty-five studies applied the United Kingdom (UK) Brain Bank diagnostic criteria [23], twenty-seven studies used the Movement Disorder Society (MDS) clinical diagnostic criteria [24], and one study followed the National Institute of Neurological Disorders and Stroke (NINDS) criteria [25]. Sixteen studies did not specify the diagnostic criteria used. Siderowf et al. described a specific diagnostic approach involving clinical criteria and supporting findings with DAT-SPECT [26]. Additionally, nine studies utilized cerebrospinal fluid from patients with PD enrolled in the National Institutes of Health (NIH) NeuroBioBank, where diagnostic criteria were only clearly defined for one of the participating sites (S2—PD Diagnosis Criteria).

RT-QuIC was used as αSyn-SAA in 52 studies, and PMCA was used in 13 studies. Also, five studies reported specific techniques for the amplification of αSyn. Details about sensitivity, specificity, study sample, processing sample, assay, and cut-off values to be considered as positive samples in each included study are given in Table 1. Please refer to the supplementary material for a detailed description of the TP/TN/FP/FN values and other diagnostic characteristics, such as likelihood ratio, predictive value, and accuracy (S3—Diagnostic Results).

### 3.3. Quality Assessment

**Figure 2:**
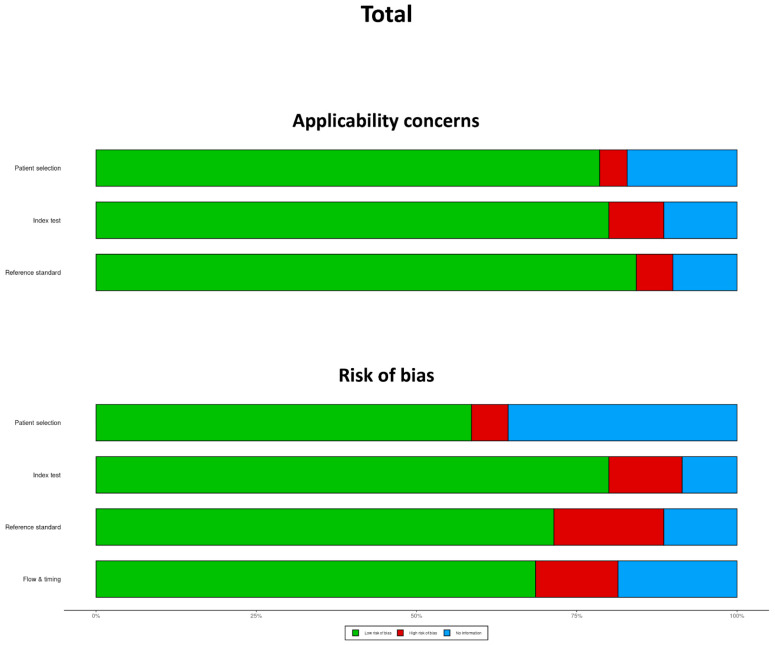
Quality assessment of all eligible studies based on robvis.

Quality assessment results based on the robvis (visualization tool) are represented in Figure 2. Overall, the risk of bias related to the reference standard and index test was generally low, while concerns about applicability were minimal in most studies. However, a high or unclear risk of bias was frequently observed for flow and timing. Among individual biomatrices, the CSF biomatrix had a higher risk of bias due to limited patient selection, flow, and timing reporting. Regarding applicability, the SMG biomatrix raised the most concerns due to insufficient reference standards and patient selection information. However, as only two studies focused on SMG, this finding should be interpreted cautiously. Consider reading supplementary material (S4—Quality) for a complete description of the quality assessment for every matrix.

### 3.4. Meta-Analysis

#### 3.4.1. Sensitivity and Specificity

**Figure 3:**
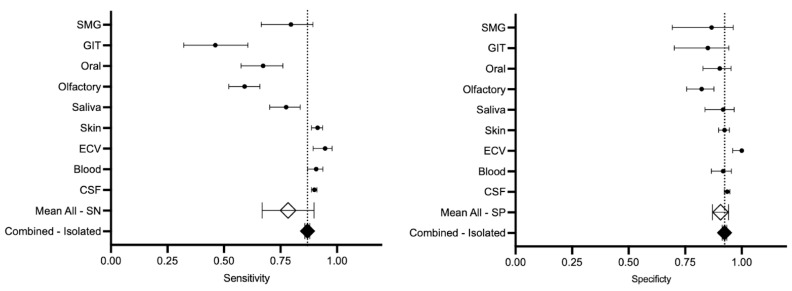
Sensitivity and specificity of all biomatrices investigated for alpha-synuclein in diagnosing Parkinson’s disease. Abbreviations: CSF, cerebrospinal fluid; ECV, extracellular vesicle; GIT, gastrointestinal; SMG, submandibular gland; SN, sensitivity; SP, specificity; transparent diamond (◇), pooled population; black diamond (◆), unique population.

**Figure 4:**
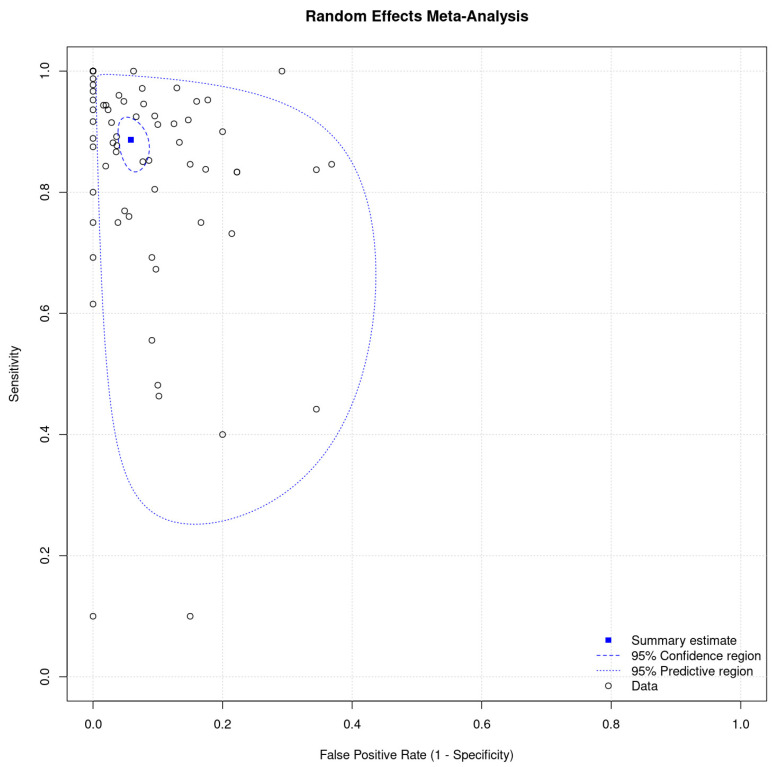
Summary receiver operating characteristic curve of αSyn-SAAs for the diagnosis of Parkinson’s disease versus non-neurodegenerative neurological control (non-synucleinopathies).

The analysis of this systematic review revealed a pooled sensitivity and specificity for αSyn-SAAs (Figure 3), including all biomatrices and types of assays, in the diagnosis of PD with single-population data of 0.86 (95% CI, 0.85–0.87) and 0.92 (95% CI, 0.91–0.93), respectively. When pooled by sample size, the sensitivity and specificity were 0.78 (95% CI, 0.66–0.89) and 0.90 (0.87–0.94). Figure 4 represents the HSROC for this analysis. Consider reading supplementary material (S5—Forest Plot) (S6—Random Effect) for forest plots for all studies and the random effect meta-analysis.

The methods with a sensitivity higher than the single-population data were biomatrices from ECV with a sensitivity of 0.94 (95% CI, 0.89–0.97), skin of 0.91 (95% CI, 0.88–0.93), blood of 0.90 (95% CI, 0.86–0.93), and CSF of 0.89 (95% CI, 0.88–0.91). The methods with a specificity higher than the single-population data were biomatrices from ECV with a sensitivity of 1.00 (95% CI, 1.00–0.95) and CSF of 0.93 (95% CI, 0.92–0.94).

There was no difference (CI includes zero) between the calculation of sensitivity or specificity with pooled or single-population statistical methods for most of the analysis, except for the comparison between the sensitivity of all analyses with (z-score: −9.22, *p*-value < 0.05) and without (z-score: −3.43, *p*-value < 0.05) CSF and the specificity of all biomatrices including CSF (z-score: −3.09, *p*-value < 0.05) (Table 2).

The differences in sensitivity and specificity with CI across the various biomatrices were assessed using the Z-test and Wald CI (S7—Difference). The summary of both tests and their significant results (Z-test with *p*-value < 0.05 and Wald CI not including 1) was that ECV exhibited the highest sensitivity, followed by CSF, blood, and skin, while saliva demonstrated moderate sensitivity. GIT, olfactory, oral, and SMG displayed the lowest sensitivity. Similarly, ECV showed the highest specificity, with CSF following closely behind. Blood, skin, and saliva exhibited moderate specificity, while olfactory, GIT, oral, and SMG had the lowest specificity.

#### 3.4.2. Positive and Negative Likelihood Ratio

**Figure 5:**
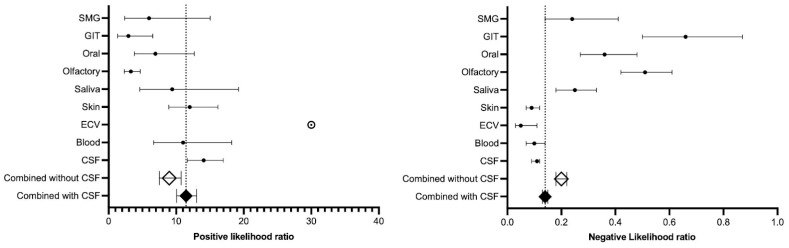
Forest plot of the positive and negative likelihood ratio of the different biomatrices. Abbreviations: CSF, cerebrospinal fluid; ECV, extracellular vesicle; GIT, gastrointestinal; SMG, submandibular gland. The white ball with the black dot is to represent the ∞ of the likelihood ratio of ECV.

The positive and negative likelihood ratios for the different biomatrices are summarized in Figure 5. The positive likelihood ratio differed significantly (no overlap in CI) between the ECV, CSF, skin, and blood group and the olfactory and GIT group. Similarly, the negative likelihood ratio showed a significant difference between the “CSF, ECV, and skin” group and the “saliva, olfactory, oral, and GIT” group. Consider reading supplementary material (S3—Diagnostic Results) for a complete description of the positive and negative likelihood ratio.

#### 3.4.3. ANOVA Results

**Figure 6:**
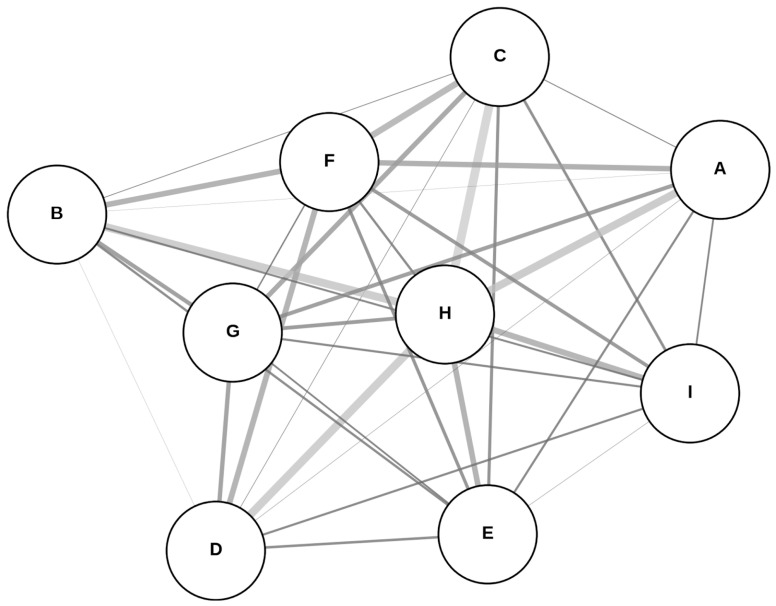
Network plot of the sensitivities. Abbreviations: (A) cerebrospinal fluid; (B) blood; (C) extracellular vesicles; (D) skin; (E) saliva; (F) olfactory; (G) oral; (H) gastrointestinal tract; (I) submandibular gland.

The network structure of biomatrices used in the meta-analysis for sensitivity is illustrated in the plot (Figure 6). The model indicates a weak relationship between sample size (n) and sensitivity (R^2^ = 0.138, *p*-value = 0.326), suggesting that sample size does not significantly predict variations in sensitivity. The estimated intercept is 0.733, representing the average sensitivity across biomatrices. Among them, biomatrices from ECV, skin, and blood exhibited the highest sensitivity, whereas those from the gastrointestinal tract and olfactory system had the lowest. The calculated I^2^ for sensitivity is 25.36%, indicating moderate heterogeneity among the sensitivity estimates across different biomatrices. For the league table (pairwise sensitivity comparisons), consider reading supplementary material (S8—League).

**Figure 7:**
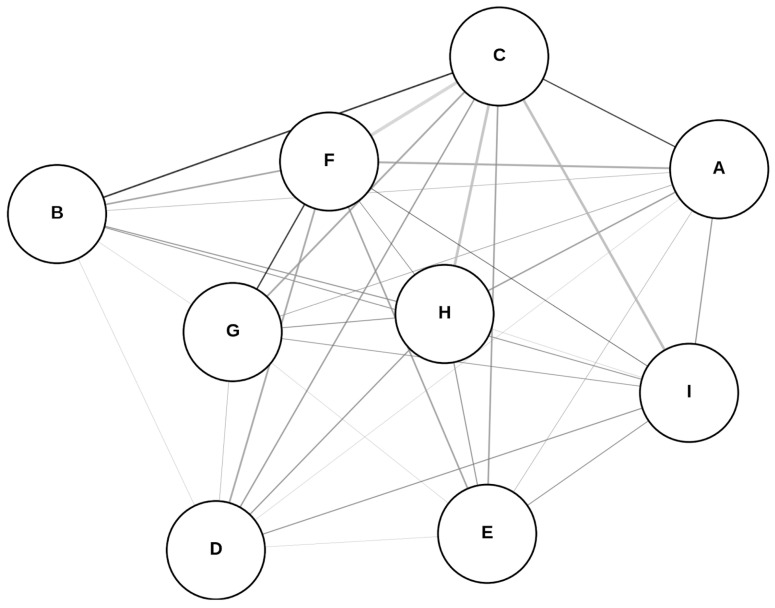
Network plot of the specificities. Abbreviations: (A) cerebrospinal fluid; (B) blood; (C) extracellular vesicles; (D) skin; (E) saliva; (F) olfactory; (G) oral; (H) gastrointestinal tract; (I) submandibular gland.

The network structure of biomatrices used in the meta-analysis for specificity is illustrated in the plot (Figure 7). The model indicates a weak relationship between sample size (n) and specificity (R^2^ = 0.065, *p*-value = 0.51), suggesting that sample size does not significantly predict variations in specificity. The estimated intercept is 0.892, representing the average specificity across biomatrices. ECV demonstrated the highest specificity, whereas the olfactory biomatrix had the lowest accuracy. Notably, moderate-to-high heterogeneity in specificity differences was observed (I^2^ = 66.69%). For the league table (pairwise specificity comparisons), consider reading supplementary material (S8—League).

The network meta-analysis revealed that ECV demonstrated perfect specificity (100%), resulting in an infinite diagnostic odds ratio (DOR), making it the most reliable diagnostic biomatrix. Cerebrospinal fluid and skin followed closely, with high sensitivity (~90%), specificity (~92–94%), and DOR values exceeding 129, indicating strong diagnostic performance with relatively low variability (SE: 16.1–29.1). Blood also exhibited a high DOR (109.4) but with more significant uncertainty (SE: 37.5). In contrast, the gastrointestinal tract had the weakest diagnostic ability, with the lowest DOR (4.45) and high SE (2.34), suggesting considerable variability in performance. Mid-tier biomatrices (saliva, submandibular gland [SMG], oral) showed moderate diagnostic effectiveness, while olfactory performed poorly, with a low DOR (6.49) and reduced diagnostic reliability. The standard error (SE) of DOR highlighted the confidence in these rankings, with higher SE values indicating greater uncertainty in diagnostic effectiveness (Table 3).

#### 3.4.4. Comparison Between RT-QuIC and PMCA

**Figure 8:**
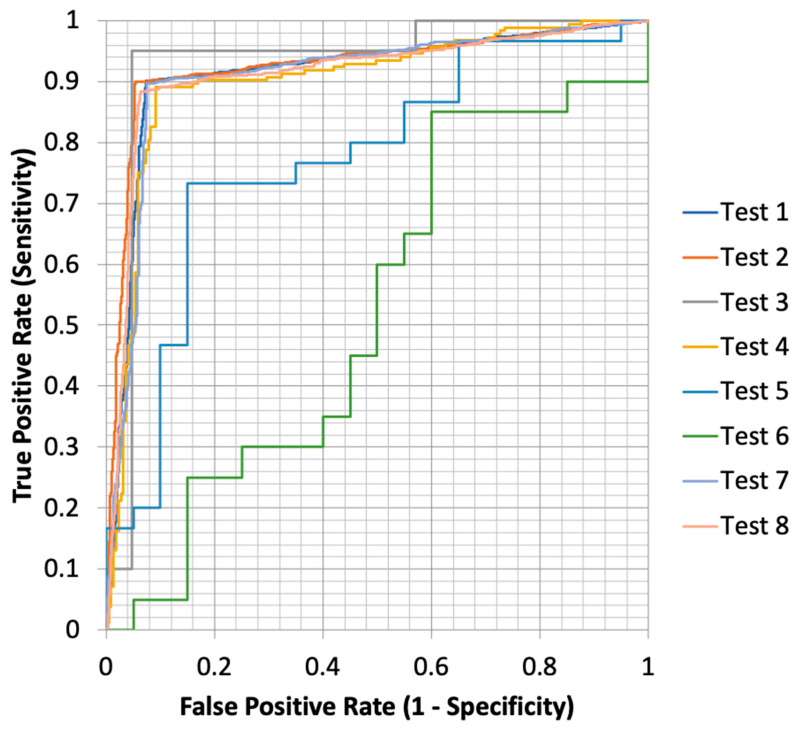
ROC curve plot of RT-QuIC and PMCA tests. Note: Test 1, CSF PMCA; Test 2, CSF RT-QuIC; Test 3, Skin PMCA; Test 4, Skin RT-QuIC; Test 5, GIT PMCA; Test 6, GIT RT-QuIC; Test 7, Combined (CSF, skin, GIT) PMCA; Test 8, Combined (CSF, skin, GIT) RT-QuIC.

RT-QuIC and PMCA techniques were reported for CSF, skin, and GIT. We also include data regarding the combination of these three samples. Therefore, a total of eight analyses were performed. Sensitivity ranged from 0.100 to 0.900, with CSF PMCA and combined (CSF, skin, and GIT) PMCA demonstrating the highest sensitivity, while GIT RT-QuIC had the lowest. Specificity values spanned from 0.500 to 0.960, with GIT RT-QuIC again showing the lowest specificity, whereas combined (CSF, skin, and GIT) RT-QuIC performed the best (Figure 8). Youden’s Index, which measures the overall effectiveness of a test, varied across tests, with combined RT-QuIC achieving the highest score, indicating better diagnostic accuracy. A Z-test comparing Youden’s indices showed significant differences between certain tests, suggesting variation in their ability to distinguish between true positives and negatives. The ROC curves and AUC values further supported these findings, demonstrating that some tests had a greater discriminative power. DeLong’s test, used to compare AUCs statistically, confirmed that differences between some tests were statistically significant, indicating superior performance of certain models over others. Overall, CSF PMCA, Combined PMCA, and Combined RT-QuIC showed higher reliability, while GIT PMCA and GIT RT-QuIC tests exhibited lower diagnostic performance (S9—Assay).

## 4. Discussion

### 4.1. General

In this systematic review and meta-analysis, we evaluated the diagnostic performance of different biomatrices for the seed amplification assay of αSyn-SAAs in PD. Our findings revealed a pooled sensitivity and specificity of αSyn-SAAs across all biomatrices and assay types of 0.86 (95% CI, 0.85–0.87) and 0.92 (95% CI, 0.91–0.93), respectively. When weighted by sample size, sensitivity was slightly lower at 0.78 (95% CI, 0.66–0.89), while specificity remained high at 0.90 (95% CI, 0.87–0.94). Among the tested biomatrices, ECV demonstrated the highest diagnostic accuracy, with perfect specificity (100%) and an infinite diagnostic odds ratio (DOR), positioning it as the most reliable biomatrix. Cerebrospinal fluid (CSF) and skin also exhibited high sensitivity (~90%) and specificity (~92–94%), with DOR values exceeding 129, underscoring their strong diagnostic performance. In contrast, the gastrointestinal tract showed the weakest diagnostic ability, with a low DOR (4.45) and high variability. Mid-tier biomatrices, including saliva, SMG, and oral samples, displayed moderate diagnostic effectiveness, whereas the olfactory region performed poorly (DOR: 6.49). These findings highlight the potential of ECV, CSF, and skin as promising biomatrices for αSyn-SAAs while emphasizing the need for further standardization to optimize diagnostic reliability. CSF PMCA, Combined PMCA, and Combined RT-QuIC showed the highest sensitivity, specificity, and overall diagnostic accuracy. At the same time, GIT PMCA and GIT RT-QuIC demonstrated the lowest performance, as confirmed by ROC analysis and DeLong’s test.

### 4.2. Cerebrospinal Fluid

Methods for obtaining samples from CSF are time-consuming and can be related to many drawbacks, involving patient psychological effects and even risks directly associated with the procedure. In our meta-analysis, tests with CSF biomatrix were ranked second best, and the sensitivity was 0.89 (95% CI, 0.88–0.91) and specificity 0.93 (95% CI, 0.92–0.94), making them one of the most reliable biomatrices for PD diagnosis. Interestingly, some authors proposed a correlation between PD progression and pathology, in which reduced levels of total αSyn and increased seeding activity in CSF correlate with disease severity, progression, and αSyn pathology burden [28]. Also, due to distinct seeding properties and aggregation kinetics, CSF αSyn-SAAs can help differentiate PD from other neurodegenerative disorders, such as multiple system atrophy (MSA) [27]. Moreover, CSF-based αSyn-seed amplification assays (αSyn-SAAs) can detect pathological αSyn aggregates even in prodromal or early-stage PD, suggesting their potential as an early diagnostic tool [32].

### 4.3. Blood

Blood-based αSyn testing offers a minimally invasive alternative to CSF collection, making it more practical for large-scale screening and longitudinal monitoring of PD. We found a pooled sensitivity of 0.90 (95% CI, 0.86–0.93) and specificity of 0.91 (95% CI, 0.86–0.95). However, αSyn is present in plasma, serum, platelets, erythrocytes, and ECV, with the highest concentration found in red blood cells (~99% of total αSyn in the blood), so the distribution can affect assay sensitivity and specificity [75]. Also, blood-based αSyn assays are complicated by contamination from peripheral sources (e.g., erythrocytes, platelets) and pre-analytical factors (e.g., hemolysis, storage conditions), leading to variability in reported results [76].

### 4.4. Neuronal Exosomes/Extracellular Vesicles

Neuronal-derived ECV NDEVs are enriched with central nervous system (CNS)-derived αSyn, reducing peripheral contamination and increasing specificity for neurodegenerative disease diagnostics [77]. In the current meta-analysis, αSyn amplification assays from ECV had the highest specificity with 1.0 (95% CI, 1.0–1.0) and a diagnostic odds ratio of 100%. This is a recent finding for the literature, especially when compared to previous meta-analyses that based their diagnosis data on CSF samples [78]. Neuronal exosomes contain oligomeric and phosphorylated αSyn (p-αSyn), which are more closely linked to disease pathology than total αSyn, and exosomal αSyn is more stable in circulation compared to free αSyn, making it a more reliable biomarker for longitudinal studies [79].

All the references included in the meta-analysis performed anti-neuronal cell adhesion molecule L1 antibody. Different immunocapture antibodies and ECV isolation techniques significantly influence the specificity and purity of NDEVs. Conventional methods such as differential ultracentrifugation and size exclusion chromatography isolate ECVs based on size and density but do not allow for cell-type specificity [80]. In contrast, immunocapture techniques use antibodies targeting neuronal surface markers to enrich for NDEVs. Two commonly used targets are L1 cell adhesion molecule (L1CAM) and neural cell adhesion molecule (NCAM). L1CAM has been widely used in early NDEV studies due to its high expression in neurons [58]; however, recent evidence suggests that L1CAM may also exist in a soluble, non-vesicular form, raising concerns about its reliability for capturing true vesicle-associated proteins [81]. NCAM, another neuronal adhesion molecule, may be more tightly associated with the vesicle membrane, but it is less validated in this context and is also expressed in other cell types such as natural killer (NK) cells and muscle tissue, potentially reducing its neuronal specificity [82]. Therefore, both the choice of antibody and the isolation method can significantly affect the yield, purity, and interpretability of NDEV preparations.

### 4.5. Skin

Interestingly, several studies have identified a proximal-to-distal gradient of abnormal αSyn in PD, with the cervical region (compared to the lumbar or distal limb) showing the highest levels of positivity [83]. Both skin and central nervous system pathology in PD exhibit αSyn abnormalities with commonalities and distinctions. In both tissues, αSyn staining is immunoreactive for p-αSyn using specific antibodies such as 5G4 and syn211 after proteinase K digestion [84]. Deposition of p-αSyn is primarily restricted to unmyelinated fibers in both the skin and CNS, with specific marker differences—vasoactive intestinal peptide (VIP)-, tyrosine hydroxylase (TH)-, substance P (SP)-, and calcitonin gene-related peptide (CGRP)-positive fibers in the skin, whereas the CNS predominantly involves SP- and TH-immunoreactive neurons [85]. Additionally, autonomic fibers are more affected than somatosensory fibers in the skin [86], whereas, in the CNS, autonomic structures are implicated in early stages, with sensory association areas involved later [87]. Both systems also show significant neuronal or nerve fiber loss, with intraepidermal nerve fiber density, mean axonal length, and intraepidermal total nerve fiber length markedly reduced in the skin, demonstrating a length-dependent loss [41]. Similarly, in the CNS, neuronal loss occurs in critical regions such as the substantia nigra, vagal and glossopharyngeal nuclei, reticular formation, and coeruleus complex, alongside impaired axonal transport. Loss of substance P-positive fibers and neurons is observed in both the skin and CNS, reinforcing shared neurodegenerative mechanisms across both systems [88].

### 4.6. Saliva

Saliva collection is a simple, cost-effective, and non-invasive method compared to CSF or blood, making it ideal for large-scale screening and repeated measurements. Saliva contains total αSyn, oligomeric αSyn (o-αSyn), and p-αSyn, with p-αSyn and o-αSyn being more disease-specific [89]. Elevated o-αSyn and p-αSyn in saliva are better indicators of PD than total αSyn, as these forms are associated with disease pathology [90]. Studies show that total αSyn levels are reduced in the saliva of PD patients compared to healthy controls, possibly due to neurodegeneration affecting salivary gland innervation [91]. Saliva showed moderate diagnostic effectiveness with DOR 25.41 (SE: 16.12).

### 4.7. Olfactory Mucosa

The olfactory mucosa can be accessed non-invasively using a nasal swab or with a minimally invasive biopsy, making it a promising biomatrix for early PD detection. Studies have confirmed the presence of misfolded αSyn in olfactory mucosa samples from PD patients but rarely in healthy controls [69]. Hyposmia is an early symptom in PD, often appearing years before motor symptoms, and correlates with αSyn accumulation in the olfactory mucosa [92]. p-αSyn in OM samples shows higher specificity for PD than total αSyn, as p-αSyn is more closely linked to pathological aggregation [46]. In our analysis, olfactory samples performed poorly, with a low DOR (6.49) and reduced diagnostic reliability.

### 4.8. Oral Mucosa

αSyn has been detected in oral mucosal tissues, including the buccal mucosa and SMG, making it a potential biomatrix for PD diagnosis. The presence of αSyn in saliva and oral mucosa could reflect systemic neurodegenerative processes, potentially linking to central nervous system pathology [91]. The oral microbiome may influence αSyn misfolding, similar to gut microbiota interactions, suggesting a possible environmental factor in PD pathology [93]. In the current study, oral mucosa had moderate diagnostic effectiveness similar to the SMG and saliva.

### 4.9. Gastrointestinal Tract (Rectum/Sigmoid/Antrum)

αSyn aggregation is observed in enteric neurons before motor symptoms appear, supporting the gut-first hypothesis of PD [94]. Also, αSyn aggregates are present in colonic tissue up to 20 years before PD diagnosis, making it a potential biomarker for early detection [95]. Misfolded αSyn has been detected in the stomach, colon, rectum, and esophageal biopsies, with the colon being one of the most frequently studied regions [96]. The GIT source of the αSyn sample had the lowest sensitivity and ranked as the worst test regarding effectiveness. However, it is worth mentioning that there was no significance between the multiple comparisons with other tests in the network plot of specificity.

### 4.10. Submandibular Gland

SMG biopsies have shown pathological αSyn aggregates in PD patients, supporting the oral region as an accessible biomatrix for detecting disease markers. In the current study, SMG had moderate diagnostic effectiveness similar to oral mucosa and saliva.

### 4.11. RT-QuIC Versus PMCA

RT-QuIC and PMCA are highly sensitive assays that detect misfolded proteins in neurodegenerative diseases. Our meta-analysis did not reveal significant differences between RT-QuIC and PMCA for detecting misfolded proteins in CSF, skin, and GIT samples. However, PMCA demonstrated higher sensitivity, while RT-QuIC exhibited greater specificity. RT-QuIC employs recombinant proteins and shaking-induced amplification, providing rapid and specific results within 12–48 h, making it suitable for diagnostics [97]. In contrast, PMCA uses brain homogenates and sonication, offering higher sensitivity but requiring several days and carrying a higher risk of contamination [98]. While both methods are valuable, RT-QuIC is preferred for clinical applications due to its speed and real-time fluorescence detection, whereas PMCA is primarily used in research [99]. A detailed comparison of these techniques is provided in Table 4.

## 5. Limitations

The most common limitation of meta-analysis is that it lacks individual patient data due to aggregate outcomes, and no raw data are provided by most studies for individual patient data to be performed. The protocols of the studies and their robustness were all adequate, but they still lacked some critical descriptions, such as the criteria for diagnosing PD. We did not perform sensitivity of the results to provide the raw data from the literature and avoid possible misunderstanding; different types of statistics were performed to reveal the relationship between the matrices. There are also limitations regarding publication bias, in that positive results are more likely to be reported, which can be observed among the different biomatrices. The heterogeneity of the studies was assessed and described to provide the reliability of the pooled estimates, which, for most studies, was moderate.

## 6. Future Directions

Despite promising results, several challenges hinder the widespread clinical adoption of αSyn as a biomarker for PD. Variability in assay protocols, pre-analytical sample handling, and patient heterogeneity contribute to inconsistencies in diagnostic performance. Different biomatrices contain αSyn at varying concentrations, complicating direct comparisons and standardization. Ongoing efforts to establish standardized protocols will improve reproducibility and ensure consistent diagnostic outcomes across different research settings.

A significant challenge in blood- and saliva-based αSyn assays is contamination from peripheral sources, particularly red blood cells containing abundant αSyn. To address this, future studies should focus on refining methods to selectively isolate neuron-derived αSyn, such as ECV enrichment or optimized immunoprecipitation techniques. Additionally, pre-analytical variables—including temperature, handling time, and storage conditions—must be carefully controlled, as they can influence αSyn stability and aggregation, ultimately affecting test reliability. Notably, blood-based αSyn biomarkers hold the potential for monitoring treatment responses in clinical trials targeting αSyn pathology, making their optimization particularly relevant for assessing disease-modifying therapies.

Given its early involvement in PD pathology, the olfactory mucosa has emerged as a promising source for early αSyn detection. However, challenges such as variability in sample quality, contamination with nasal epithelium, and heterogeneous αSyn expression patterns limit its clinical applicability. To determine its actual diagnostic utility, further research is needed to establish standardized collection and processing techniques alongside large-scale validation studies.

Detecting misfolded and aggregated αSyn remains a fundamental challenge due to its low abundance in biological fluids. Highly sensitive detection methods, such as RT-QuIC and seed amplification assays, have shown promise but require specialized equipment and technical expertise, restricting their widespread use. Future efforts should focus on improving assay accessibility by reducing costs and streamlining protocols to facilitate broader clinical implementation.

Another major limitation is that αSyn aggregation is not exclusive to PD but is also observed in MSA and dementia with Lewy bodies (DLB), complicating differential diagnosis. Given the overlapping pathology among synucleinopathies, incorporating αSyn biomarkers into the ATN (Amyloid, Tau, Neurodegeneration) framework could provide a more comprehensive approach to disease classification. While the ATN model currently focuses on Alzheimer’s disease, expanding it to include αSyn pathology (e.g., misfolded αSyn detected through seed amplification assays or p-αSyn quantification) could enhance its applicability to PD and related disorders. Furthermore, integrating αSyn measurements with additional neurodegenerative biomarkers—such as DJ-1 for oxidative stress or inflammatory cytokines—may improve diagnostic specificity and facilitate differentiation between PD and other synucleinopathies. Future research should explore how αSyn biomarkers fit within an expanded ATN framework to refine diagnostic accuracy and enable a more targeted approach to neurodegenerative disease classification.

Finally, while CSF analysis and tissue biopsies (e.g., from the skin, gastrointestinal tract, or SMG) provide valuable diagnostic insights, their invasive nature limits patient acceptance and feasibility for routine screening. Future research should prioritize the development of noninvasive or minimally invasive alternatives—such as saliva, blood, or urine-based assays—that offer comparable diagnostic accuracy while improving patient accessibility and compliance.

By addressing these challenges through technological advancements, biomarker standardization, and large-scale validation studies, αSyn-based diagnostics have the potential to become an integral component of PD diagnosis and disease monitoring in both clinical and research settings.

Our meta-analysis highlights the need for prioritizing certain biomatrices for further investigation in SAA for αSyn detection. Given their high diagnostic performance and minimally invasive collection methods, the rank analysis identified ECV and blood samples as the top candidates for future research. While skin and CSF demonstrated similar effectiveness to blood-based assays, their invasive nature makes them less favorable for widespread clinical application. Saliva and SMG samples exhibited considerable variability in diagnostic accuracy, warranting further investigation with more refined analytical algorithms to enhance reliability. Conversely, biomatrices from the gastrointestinal tract, olfactory mucosa, and oral cavity demonstrated limited diagnostic utility in our analysis, suggesting that future research should focus on more promising alternatives.

### Limitations of αSyn-SAA

A positive αSyn-SAA test confirms the presence of aggregated αSyn but does not provide insight into the underlying disease mechanisms. It cannot determine the biological cause of aggregation, whether due to genetic, toxic, or infectious factors [100]. Additionally, αSyn-SAA positivity may not correlate with disease severity or progression, as some studies have found no association with motor dysfunction, cognitive impairment, or autonomic symptoms [26]. The test also fails to distinguish between different α-synucleinopathies, such as PD and dementia with Lewy bodies. However, kinetic measures may help differentiate MSA with limited accuracy [38]. Moreover, its diagnostic performance varies across study cohorts, raising concerns about its clinical applicability. Lastly, αSyn-SAA cannot be used to assess treatment response, as it remains positive regardless of disease-modifying interventions, as demonstrated in trials like cinpanemab [101]. These limitations highlight the need for additional biomarkers to enhance diagnostic and prognostic capabilities in α-synucleinopathies.

## 7. Conclusions

When comparing different biomatrices for αSyn investigation in PD, CSF and ECVs stand out for their high diagnostic accuracy, with CSF showing strong sensitivity (~89%) and specificity (~93%). In comparison, ECVs demonstrate perfect specificity (100%), making them an up-and-coming, though technically challenging, option. Blood-based αSyn-SAA, while non-invasive, faces higher variability due to peripheral αSyn sources, though studies suggest potential in isolating neuron-derived αSyn. Saliva and oral mucosa offer non-invasive accessibility, but αSyn levels in these biomatrices exhibit moderate diagnostic accuracy, requiring refinement in detection methods. GIT biopsies, particularly from the colon, have been explored for early αSyn deposition, but invasiveness and inconsistent findings limit their utility. The olfactory mucosa has been investigated due to its early involvement in PD, yet low diagnostic reliability hinders clinical application. Similarly, SMG biopsies have shown high specificity and early-stage αSyn accumulation, making them a strong peripheral biomarker candidate, though biopsy success rates remain a limitation. CSF and ECVs currently hold the highest diagnostic potential, while blood, saliva, and SMG biopsies offer promising but developing alternatives for non-invasive or minimally invasive testing.

## Figures and Tables

**Table 1:** Types of biospecimens for alpha-synuclein seed amplification assays in Parkinson’s disease.

Sample	Sensitivity	Specificity	N	Sample from PD	Processing of Sample	Assay	Cut-Off Value	Reference
Cerebrospinal fluid	95.24%	100.00%	21 PD, 2 PSP, 35 HC	Biopsy	NA	RT-QuIC	Mean of negative controls +2SD	Fairfoul et al. (2016) [27]
	88.16%	96.92%	76 PD, 10 MSA, 65 NNC	Biopsy	NA	PMCA	≥50 FU	Shahnawaz et al. (2017) [28]
	91.67%	100.00%	12 PD, 2 PSP, 1 CBD, 12 HC	Biopsy	NA	RT-QuIC	Mean of all samples +3SD	Groveman et al. (2018) [29]
	10.00%	100.00%	10 PD, 10 LBD, 10 HC	Autopsy	Frozen	RT-QuIC	50% of maximum value	Candelise et al. (2019) [30]
	90.00%; 40.00%	80.00%; 80.00%	10 PD LRRK2 negative, 10 HC; 15 PD LRRK2 positive, 10 HC	Biopsy	NA; NA	RT-QuIC	Mean of negative controls +2SD	Garrido et al. (2019) [31]
	95.24%; 97.14%	82.28%; 92.41%	105 PD, 79 HC	Biopsy	NA	PMCA	≥1000 FU	Kang et al. (2019) [32]
	100.00%	100.00%	15 PD, 5 PSP, 16 HC	Biopsy	NA	RT-QuIC	Mean of all samples +10SD	Manne et al. (2019) [33]
	85.25%	91.37%	278 PD, 278 NNC	Biopsy	NA	PMCA	≥150 FU	Ning et al. (2019) [34]
	84.31%	98.04%	51 PD, 17 MSA, 8 PSP, 51 HC	Biopsy	NA	RT-QuIC	Mean of negative controls +2SD	van Rumund et al. (2019) [35]
	94.37%	98.39%	71 PD, 62 NNC, 33 MSA	Biopsy	NA	RT-QuIC	Mean of neuropathological controls +30SD	Rossi et al. (2020) [36]
	93.62%	100.00%	94 PD, 56 NNC, 75 MSA	Biopsy	NA	PMCA	≥50 FU	Shahnawaz et al. (2020) [37]
	100.00%	100.00%	16 PD, 62 MSA, 29 HC	Biopsy	NA	PMCA	≥150 AU	Singer et al. (2020) [38]
	97.73%	100.00%	88 PD, 38 NNC, 9 PSP	Autopsy	NA	RT-QuIC	Mean background fluorescence +5SD	Bargar et al. (2021) [39]
	85.05%	92.31%	107 PD, 26 HC	Biopsy	NA	RT-QuIC	Mean of negative controls +30SD	Brockmann et al. (2021) [40]
	100.00%	100.00%	2 PD, 2 MSA, 1 PSP, 13 HC	Biopsy	NA	RT-QuIC	Mean of negative controls +3SD	Donadio et al. (2021) [41]
	100.00%	100.00%	7 PD, 27 NNC	Biopsy	NA	RT-QuIC	15% of maximum value	Mammana et al. (2021) [42]
	97.22%	87.06%	108 PD, 85 HC	Biopsy	NA	RT-QuIC	10% of maximum value	Orrù et al. (2021) [43]
	91.50%	97.14%	153 PD/PDD, 68 MSA, 35 HC	Biopsy	NA	RT-QuIC	NA	Quadalti et al. (2021) [44]
	86.67%; 96.67%	96.43%; 100.00%	30 PD, 1 MSA, 28 HC	Biopsy	NA	RT-QuIC	10% of maximum value	Russo et al. (2021) [45]
	91.30%; 100.00%	87.50%; 100.00%	23 PD, 8 NNC; 1 PD, 11 NNC	Biopsy	NA	RT-QuIC	20% of maximum value	Bongianni et al. (2022) [46]
	75.00%; 80.00%	100.00%; 100.00%	20 PD, 19 HC, 37 MSA, 23 PSP, 13 CBD	Biopsy	NA	RT-QuIC	Mean of negative controls +2SD	Compta et al. (2022) [47]
	95.00%	84.00%	20 PD, 25 HC, 1 MSA, 4 PSP, 1 CBD	Autopsy	NA	RT-QuIC	10% of maximum value	Hall et al. (2022) [48]
	91.94%	85.29%	62 PD, 34 HC	Biopsy	NA	RT-QuIC	Mean of all samples +3SD	Majbour et al. (2022) [49]
	84.62%	63.16%	13 NPH + PD/PDD, 19 NPH	Biopsy	NA	RT-QuIC	Mean of negative controls +2SD	Sakurai et al. (2022) [22]
	89.19%	96.36%	74 PD, 55 HC	Biopsy	NA	RT-QuIC	Mean of initial fluorescence at 120 h + 5SD	Poggiolini et al. (2022) [50]
	92.59%	79.17%	54 PD, 21 HC	Biopsy	NA	PMCA	≥1000 FU	Chahine et al. (2023) [51]
	94.37%	98.00%	71 PD, 2 MSA, 50 HC	Biopsy	NA	PMCA	≥50 AU	Concha-Marambio et al. (2023) [52]
	100.00%	70.83%	55 PD, 27 MSA, 7 CBD, 16 PSP, 24 HC	Biopsy	NA	PMCA	≥100 FU	Fernandes Gomes et al. (2023) [53]
	87.50%	100.00%	8 PD, 3 HC	Autopsy	Frozen	RT-QuIC	Mean of negative controls +2SD	Garrido et al. (2023) [54]
	100.00%	100.00%	6 PD, 3 MSA, 35 NNC	Biopsy	NA	RT-QuIC	Mean of all samples +3SD	Okuzumi et al. (2023) [55]
	87.71%	96.32%	545 PD, 163 HC	Biopsy	NA	PMCA	≥50 AU	Siderowf et al. (2023) [26]
	100.00%	100.00%	41 LBD, 6 PD, 37 NNC, 42 AD	Biopsy	NA	RT-QuIC	Mean of all samples +3SD	Verdurand et al. (2025) [56]
Blood	94.57%	92.19%	221 PD, 39 MSA, 10 LBD, 9 RBD, 30 PSP. 25 AD, 128 HC	Biopsy	NA	RT-QuIC	Mean of all samples +3SD	Okuzumi et al. (2023) [55]
	80.49%	90.48%	82 PD, 42 HC	Biopsy	NA	RT-QuIC	Mean of all samples +4SD	Wang et al. (2024) [57]
Neuronal exosomes/extracellular vesicles	100.00%	100.00%	30 PD, 50 HC	Biopsy	NA	Specific technique	>100 ng α-synuclein monomers	Kluge et al. (2023) [58]
	61.54%; 88.89%	100.00%; 100.00%	13 PD Parkin positive, 10 HC; 9 idiopathic PD, 10 HC	Biopsy	NA; NA	Specific technique	>500 ng α-synuclein monomers	Kluge et al. (2024) [59]
	98.75%	100.00%	80 PD, 20 HC	Biopsy	NA	RT-QuIC	Mean of all samples +5SD	Schaeffer et al. (2024) [60]
Skin	96.00%; 75.00%	96.00%; 83.33%	25 PD, 25 HC; 12 PD, 12 HC	Autopsy from scalp	Frozen tissues; FFPE tissues	RT-QuIC	Mean of all samples +10SD	Manne et al. (2020) [61]
	93.62%; 95.00%	97.67%; 95.24%	47 PD, 43 HC; 20 PD, 21 HC	Autopsy from abdomen and scalp; biopsy from C7 paravertebral and legs	Frozen tissues; fresh tissues	RT-QuIC; PMCA	Mean of all samples +3SD; NA	Wang et al. (2020) [62]
	83.33%	77.78%	6 PD, 18 HC	Biopsy from C7 paravertebral, thigh, and leg	Frozen tissues	RT-QuIC	Mean of negative controls +3SD	Donadio et al. (2021) [41]
	91.18%	90.00%	34 PD; 30 HC	Biopsy from neck, lower back, thigh, and lower leg	Frozen tissues	RT-QuIC	Mean of negative controls +5SD	Kuzkina et al. (2021) [63]
	76.92%	95.12%	13 PD, 41 NNC	Biopsy from neck, leg, and thigh	NA	RT-QuIC	15% of maximum value	Mammana et al. (2021) [42]
	84.62%	85.00%	13 PD, 10 MSA, 7 PSP, 20 HC	Biopsy	NA	RT-QuIC	FU > 20,000	Martinez-Valbuena et al. (2022) [64]
	88.24%	86.67%	34 PD, 30 NNC	Biopsy from C7, Th10, and proximal leg.	Frozen tissues	RT-QuIC	Mean of negative controls +5SD	Kuzkina et al. (2023) [65]
	92.47%	93.33%	332 PD, 285 HC	Biopsy from cervical region	Frozen tissues	Specific technique	Mean of all samples +10SD	Kuang et al. (2024) [66]
Saliva	76.00%	94.44%	75 PD, 36 HC	Biopsy	NA	RT-QuIC	Mean of all samples +2SD	Luan et al. (2022) [67]
	83.78%	82.61%	37 PD, 23 HC	Biopsy	NA	RT-QuIC	FU > 2990.5	Vivacqua et al. (2023) [68]
	75.00%	96.15%	48 PD, 26 HC	Biopsy	NA	RT-QuIC	Mean of all samples +4SD	Wang et al. (2024) [57]
Olfactory mucosa	55.55%	NA	18 PD, 11 MSA, 6 CBD, 12 PSP	Biopsy	NA	RT-QuIC	AU > 500	De Luca et al. (2019) [20]
	69.23%; 69.23%	90.91% 100.00%	13 PD, 10 MSA-C; 20 MSA-P; 11 HC	Biopsy	NA	RT-QuIC	AU > 30,000	Bargar et al. (2021) [39]
	46.34%	89.83%	41 PD, 59 HC	Biopsy	NA	RT-QuIC	Mean of all samples +3SD	Stefani et al. (2021) [69]
	44.19%; 83.72%	65.52%; 65.52%	43 PD, 6 PSP, NNC 29	Biopsy	NA	RT-QuIC	20% of maximum value	Bongianni et al. (2022) [46]
	48.15%	90.00%	27 PD, 3 MSA, 3 PSP, 30 NNC	Biopsy	NA	RT-QuIC	10% of maximum value	Kuzkina et al. (2023) [70]
Oral mucosa	67.29%	90.29%	107 PD, 99 MSA, 33 RBD, 103 HC	Biopsy of oral mucosa	Frozen tissues	Specific technique	AU > 49,219	Zheng et al. (2024) [71]
Gastrointestinal tract (rectum/sigmoid/antrum)	55.56%	90.91%	18 PD, 11 HC	Biopsy from rectum, sigmoid, and antrum	Frozen tissues	PMCA	30% of maximum value	Fenyi et al. (2019) [72]
	83.33%	77.78%	12 PD, 8 LBD, 9 HC	Autopsy gastric cardia	Formaldehyde-fixedtissues	PMCA	30% of maximum value	Fenyi et al. (2021) [73]
	100.00%; 10.00%	NA; 85.00%	2 PD, 1, LBD, 1 MSA; 20 PD, 20 HC	Autopsy from stomach; biopsy from stomach, esophagus, colon, and rectum	FFPE tissues; FFPE tissues	RT-QuIC	Mean of all samples +10SD	Shin et al. (2022) [21]
Submandibular gland	100.00%	93.75%	13 PD, 16 HC	Autopsy	FFPE	RT-QuIC	Mean of all samples +10SD	Manne et al. (2020) [74]
	73.17%	78.57%	41 PD, 14 HC	Biopsy	FFPE tissues	RT-QuIC	Mean of all samples +10SD	Chahine et al. (2023) [51]

Abbreviations: AD, Alzheimer’s disease; AU, arbitrary units; CBD, corticobasal degeneration; CI, confidence interval; FFPE, formaldehyde-fixed paraffin-embedded; FFUs, fibril-forming units; FU, fluorescence units; HC, healthy control; LBD, Lewy body dementia; MSA, multiple system atrophy; MSA-C, MSA-cerebellar type; MSA-P, MSA-parkinsonian type; NA, not available/not applicable; NNC, non-neurodegenerative neurological control; NPH, normal pressure hydrocephalus; PD, Parkinson’s disease; PDD, Parkinson’s disease dementia; PSP, progressive supranuclear palsy; RBD, rapid eye movement sleep behavior disorder; SD, standard deviation; WT, wildtype.

**Table 2:** Sensitivity and specificity of biomatrices for diagnosing Parkinson’s disease.

Biomatrices	*n*	Sensitivity (95% CI)			Specificity (95% CI)		
		Pooled ^a^	Single-Population ^b^	Z-Test	Pooled ^a^	Single-Population ^b^	Z-Test
CSF	2382	0.88 (95% CI, 0.83–0.94)	0.89 (95% CI, 0.88–0.91)	−1.08	0.93 (95% CI, 0.90–0.96)	0.93 (95% CI, 0.92–0.94)	0
Blood	303	0.88 (95% CI, 0.70–1.06)	0.90 (95% CI, 0.86–0.93)	−0.78	0.91 (95% CI, 0.89–0.93)	0.91 (95% CI, 0.86–0.95)	0
Extracellular vesicles	132	0.88 (95% CI, 0.69–1.08)	0.94 (95% CI, 0.89–0.97)	−1.70	1.00 (95% CI, 1.00–1.00)	1.00 (95% CI, 0.95–1.00)	0
Skin	536	0.88 (95% CI, 0.83–0.92)	0.91 (95% CI, 0.88–0.93)	−1.60	0.90 (95% CI, 0.86–0.94)	0.92 (95% CI, 0.89–0.94)	−1.14
Saliva	160	0.78 (95% CI, 0.71–0.84)	0.77 (95% CI, 0.70–0.83)	0.21	0.91 (95% CI, 0.81–1.01)	0.91 (95% CI, 0.83–0.96)	0
Olfactory	198	0.59 (95% CI, 0.47–0.71)	0.59 (95% CI, 0.52–0.65)	0	0.83 (95% CI, 0.71–0.95)	0.82 (95% CI, 0.75–0.87)	0.26
Oral	107	0.67 (95% CI, 0.57–0.76)	0.67 (95% CI, 0.57–0.76)	0	0.90 (95% CI, 0.82–0.95)	0.90 (95% CI, 0.82–0.95)	0
Gastrointestinal tract	52	0.59 (95% CI, 0.15–1.02)	0.46 (95% CI, 0.32–0.60)	1.32	0.84 (95% CI, 0.76–0.93)	0.85 (95% CI, 0.70–0.94)	−0.14
Submandibular gland	54	0.84 (95% CI, 0.49–1.191)	0.79 (95% CI, 0.66–0.89)	0.66	0.86 (95% CI, 0.67–1.05)	0.86 (95% CI, 0.69–0.96)	0
All ^c^ except CSF	1542	0.77 (95% CI, 0.70–0.84)	0.82 (95% CI, 0.80–0.84)	−3.43 *	0.89 (95% CI, 0.86–0.92)	0.90 (95% CI, 0.89–0.92)	−0.90
All ^c^ including CSF	3924	0.78 (95% CI, 0.66–0.89)	0.86 (95% CI, 0.85–0.87)	−9.22 *	0.90 (95% CI, 0.87–0.94)	0.92 (95% CI, 0.91–0.93)	−3.09 *

Abbreviations: CSF, cerebrospinal fluid. * significant result with *p*-value < 0.05. ^a^ pooled analysis was based on the mean weighted by the sample size. ^b^ single-population analysis was based on the inclusion of all data in a single population. ^c^ all biomatrices are CSF, blood, extracellular vesicles, skin, saliva, olfactory, oral, gastrointestinal tract, and submandibular gland.

**Table 3:** The results of network meta-analysis based on specificity.

Rank	Biomatrix	Relative Sensitivity (95% CI)	Relative Specificity (95% CI)	DOR (SE)
1	Extracellular vesicles	0.94 (0.90–0.98)	1.00 (1.00–1.00)	∞ (Perfect specificity)
2	Cerebrospinal fluid	0.89 (0.88–0.91)	0.93 (0.92–0.94)	131.25 (16.14)
3	Skin	0.91 (0.89–0.93)	0.92 (0.90–0.94)	129.16 (29.12)
4	Blood	0.90 (0.87–0.94)	0.91 (0.87–0.95)	109.44 (37.46)
5	Saliva	0.77 (0.71–0.83)	0.91 (0.85–0.97)	38.38 (16.80)
6	Submandibular gland	0.79 (0.68–0.90)	0.86 (0.74–0.98)	25.41 (16.12)
7	Oral mucosa	0.67 (0.58–0.76)	0.90 (0.84–0.96)	19.13 (7.49)
8	Olfactory mucosa	0.58 (0.51–0.65)	0.90 (0.84–0.96)	6.49 (1.63)
9	Gastrointestinal tract	0.44 (0.30–0.57)	0.85 (0.73–0.96)	4.45 (2.34)

Abbreviations: CI, confidence interval; DOR, diagnostic odds ratio; SE, standard error. Note: The analysis was conducted using a network meta-analysis approach, comparing the diagnostic performance of various biomatrices. Relative sensitivity and specificity were estimated with 95% CI, while the DOR was calculated to assess overall diagnostic effectiveness. The SE of DOR was derived to quantify uncertainty in the estimates. Higher DOR values indicate superior diagnostic performance, while higher SE values suggest greater variability in the estimates.

**Table 4:** Comparison between RT-QuIC and PMCA.

Feature	RT-QuIC (Real-Time Quaking-Induced Conversion)	PMCA (Protein Misfolding Cyclic Amplification)
Principle	Misfolded proteins seed the conversion of recombinant proteins, detected via fluorescence	Cyclic amplification of misfolded proteins using brain homogenates and sonication
Amplification Mechanism	Shaking-induced conversion	Sonication-induced conversion
Readout	Fluorescence detection in real time	End-point detection (Western blot, Thioflavin T)
Assay Duration	12–48 h	Several days
Sensitivity	High	Very high (more sensitive than RT-QuIC)
Specificity	High	High, but depends on conditions
Substrate Used	Recombinant proteins	Brain homogenates (more complex)
Risk of Contamination	Lower	Higher due to exponential amplification
Equipment Required	Fluorescence plate reader	Sonicator and specialized incubation equipment
Quantification Capability	Yes, real-time fluorescence monitoring	No, usually an endpoint assay

## Data Availability

All new data created is provided as Supplementary Material.

## Supplementary Materials

The following supporting information can be downloaded at https://www.mdpi.com/article/10.3390/clinpract15060107/s1, S1: Search Strategy; S2: PD diagnosis criteria; S3: Diagnostic Results; S4: Quality; S5: Forest Plot; S6: Random Effect; S7: Difference; S8: League; S9: Assay; S10: PRISMA Checklist.

## Abbreviations

The following abbreviations are used in this manuscript:

αSyn	alpha-synuclein
αSyn-SAA	alpha-synuclein seed amplification assay
CI	confidence interval
CNS	central nervous system
DOR	diagnostic odds ratio
ECV	extracellular vesicle
GIT	gastrointestinal tract
MSA	multiple system atrophy
OM	olfactory mucosa
p-αSyn	phosphorylated alpha-synuclein
PD	Parkinson’s disease
PMCA	protein misfolding cyclic amplification
RT-QuIC	real-time quaking-induced conversion
SMG	submandibular gland
