# Supplementary material for: Alpha-Synuclein Seed Amplification Assays in Parkinson’s Disease: A Systematic Review and Network Meta-Analysis
Source: Clin Pract. 2025 Jun 3;15(6):107. doi: 10.3390/clinpract15060107 (PMC12192484; doi:10.3390/clinpract15060107)
Supplement: Supplementary file 1 [file clinpract-15-00107-s001.zip › clinpract-3631320-supplementary.pdf]

Article: Alpha-Synuclein Seed Amplification Assays In Parkinson's Disease: A Systematic Review And Network Meta-Analysis
Rissardo et al. (2025)

Supplementary Material

S1. Search Strategy

The table presents the FreeText and MeSH search terms used for literature retrieval in the U.S. National Library of Medicine. It includes keyword-based FreeText terms and standardized Medical Subject Headings (MeSH) terms to ensure comprehensive coverage of relevant studies. This structured approach facilitates reproducibility and enhances the precision of the literature search strategy.

S2. PD diagnosis criteria

The table provides an overview of the diagnostic criteria for Parkinson's disease used in each included study. It details the specific clinical or biomarker-based criteria applied, including established guidelines such as the UK Parkinson's Disease Society Brain Bank criteria, the Movement Disorder Society clinical diagnostic criteria, or other study-specific definitions. This allows for a comparative evaluation of diagnostic approaches across studies.

S3. Diagnostic Results

The table presents the diagnostic results of the included studies, detailing the number of individuals with Parkinson's disease and controls. It includes key diagnostic performance metrics: true positives, false negatives, true negatives, and false positives. Additionally, sensitivity, specificity, positive likelihood ratio, negative likelihood ratio, positive predictive value, negative predictive value, and overall accuracy are reported, each with their corresponding 95% confidence intervals.

S4. Quality

The table presents a quality assessment of the results for each study, categorized according to biomatrices. The evaluation was divided into two domains: risk of bias and applicability. The assessment included four key aspects: patient selection, index test, reference standard, and flow and timing.

S5. Forest Plot

To present the results visually, forest plots were generated for all analyses, illustrating effect sizes and confidence intervals for each study. These plots will display individual study estimates along with their corresponding 95% confidence intervals, allowing for a clear comparison of diagnostic performance metrics such as sensitivity, specificity, and likelihood ratios.

S6. Random Effect

A random-effects meta-analysis was conducted to evaluate diagnostic performance, including a summary receiver operating characteristic (SROC) curve analysis to illustrate overall accuracy with a 95% confidence and

prediction region. Additionally, a forest plot was generated to display study-level sensitivity and specificity estimates with corresponding 95% confidence intervals, accounting for between-study heterogeneity.

S7. <u>Difference</u>

The Z-test for comparing two proportions was used to determine whether a significant difference existed between the proportions in two groups, with the Wald confidence interval (CI) applied to estimate the difference. To summarize the results for sensitivity and specificity, a Venn diagram was utilized to illustrate the logical relationships between sets.

S8. <u>League</u>

Two league tables were constructed to present pairwise comparisons of sensitivity and specificity within the meta-analysis. This table facilitates direct comparisons between studies, ranking their diagnostic performance while accounting for variability across datasets.

S9. <u>Assay</u>

RT-QuIC and PMCA techniques were compared across cerebrospinal fluid, skin, and gastrointestinal tract samples. The analysis included Youden's Index (J) to assess overall diagnostic performance, DeLong's Test for statistical comparison of AUCs, and probabilistic results for area under the curve (AUC) estimates.

S10. <u>PRISMA Checklist</u>

The PRISMA (Preferred Reporting Items for Systematic Reviews and Meta-Analyses) checklist was used to ensure transparency and methodological rigor in the systematic review. It includes key reporting items covering study selection, data extraction, risk of bias assessment, and synthesis of results, enhancing reproducibility and reliability.

S1. Search Strategy

Table. FreeText and MeSH search terms in the U.S. National Library of Medicine.		
**Query**	**Search Terms**	**Re-sults**
(alpha-synuclein) AND (real-time quaking-induced conversion)	"real-time"[All Fields] AND "quaking-induced"[All Fields] AND ("conversion"[All Fields] OR "conversions"[All Fields]) AND ("alpha synuclein"[MeSH Terms] OR "alpha synuclein"[All Fields] OR ("alpha"[All Fields] AND "synuclein"[All Fields]) OR "alpha synuclein"[All Fields])	113
(alpha-synuclein) AND (protein misfolding cyclic amplification)	("alpha synuclein"[MeSH Terms] OR "alpha synuclein"[All Fields] OR ("alpha"[All Fields] AND "synuclein"[All Fields]) OR "alpha synuclein"[All Fields]) AND (("protein s"[All Fields] OR "proteinous"[All Fields] OR "proteins"[MeSH Terms] OR "proteins"[All Fields] OR "protein"[All Fields]) AND ("misfold"[All Fields] OR "misfolded"[All Fields] OR "misfold-ing"[All Fields] OR "misfoldings"[All Fields] OR "misfolds"[All Fields]) AND ("cyclic"[All Fields] OR "cyclics"[All Fields]) AND ("amplificate"[All Fields] OR "amplificates"[All Fields] OR "amplification"[All Fields] OR "amplifications"[All Fields]))	51
(alpha-synuclein) AND (cerebrospi-nal fluid)	("alpha synuclein"[MeSH Terms] OR "alpha synuclein"[All Fields] OR ("alpha"[All Fields] AND "synuclein"[All Fields]) OR "alpha synuclein"[All Fields]) AND ("cerebrospinal fluid"[MeSH Subheading] OR ("cerebrospinal"[All Fields] AND "fluid"[All Fields]) OR "cerebrospinal fluid"[All Fields] OR "cerebrospinal fluid"[MeSH Terms])	897
(alpha-synuclein) AND (blood)	("alpha synuclein"[MeSH Terms] OR "alpha synuclein"[All Fields] OR ("alpha"[All Fields] AND "synuclein"[All Fields]) OR "alpha synuclein"[All Fields]) AND ("blood"[MeSH Subheading] OR "blood"[All Fields] OR "blood"[MeSH Terms] OR "bloods"[All Fields] OR "haematology"[All Fields] OR "hematology"[MeSH Terms] OR "hematology"[All Fields] OR "hae-matoma"[All Fields] OR "hematoma"[MeSH Terms] OR "hematoma"[All Fields] OR "haemorrhage"[All Fields] OR "hem-orrhage"[MeSH Terms] OR "hemorrhage"[All Fields] OR "haemorrhages"[All Fields] OR "hemorrhages"[All Fields] OR "haemorrhagic"[All Fields] OR "haemorrhaging"[All Fields] OR "hematologies"[All Fields] OR "haematomas"[All Fields] OR "hematomas"[All Fields] OR "hematoma s"[All Fields] OR "hematomae"[All Fields] OR "hemorrhaged"[All Fields] OR "hemorrhagic"[All Fields] OR "hemorrhagical"[All Fields] OR "hemorrhaging"[All Fields])	1582
(alpha-synuclein) AND (skin)	("alpha synuclein"[MeSH Terms] OR "alpha synuclein"[All Fields] OR ("alpha"[All Fields] AND "synuclein"[All Fields]) OR "alpha synuclein"[All Fields]) AND ("skin"[MeSH Terms] OR "skin"[All Fields])	277
(alpha-synuclein) AND (extracellu-lar vesicles)	("alpha synuclein"[MeSH Terms] OR "alpha synuclein"[All Fields] OR ("alpha"[All Fields] AND "synuclein"[All Fields]) OR "alpha synuclein"[All Fields]) AND ("extracellular vesicles"[MeSH Terms] OR ("extracellular"[All Fields] AND "vesi-cles"[All Fields]) OR "extracellular vesicles"[All Fields])	258
(alpha-synuclein) AND (saliva)	("alpha synuclein"[MeSH Terms] OR "alpha synuclein"[All Fields] OR ("alpha"[All Fields] AND "synuclein"[All Fields]) OR "alpha synuclein"[All Fields]) AND ("saliva"[MeSH Terms] OR "saliva"[All Fields] OR "salivas"[All Fields] OR "saliva s"[All Fields])	83
(alpha-synuclein) AND (olfactory mucosa)	("alpha synuclein"[MeSH Terms] OR "alpha synuclein"[All Fields] OR ("alpha"[All Fields] AND "synuclein"[All Fields]) OR "alpha synuclein"[All Fields]) AND ("olfactory mucosa"[MeSH Terms] OR ("olfactory"[All Fields] AND "mucosa"[All Fields]) OR "olfactory mucosa"[All Fields])	43
(alpha-synuclein) AND (gastroin-testinal tract)	("alpha synuclein"[MeSH Terms] OR "alpha synuclein"[All Fields] OR ("alpha"[All Fields] AND "synuclein"[All Fields]) OR "alpha synuclein"[All Fields]) AND ("gastrointestinal tract"[MeSH Terms] OR ("gastrointestinal"[All Fields] AND "tract"[All Fields]) OR "gastrointestinal tract"[All Fields])	356
(alpha-synuclein) AND (subman-dibular gland)	("alpha synuclein"[MeSH Terms] OR "alpha synuclein"[All Fields] OR ("alpha"[All Fields] AND "synuclein"[All Fields]) OR "alpha synuclein"[All Fields]) AND ("submandibular gland"[MeSH Terms] OR ("submandibular"[All Fields] AND "gland"[All Fields]) OR "submandibular gland"[All Fields])	43

S2. PD diagnosis criteria

Table. Parkinson's disease diagnosis criteria	
**Reference**	Description
Cerebrospinal Fluid	
Fairfoul (2016) [22]	UK Brain Bank diagnostic criteria
Shahnawaz (2017) [23]	UK Brain Bank diagnostic criteria
Groveman (2018) [24]	MDS clinical diagnostic criteria
Candelise (2019) [25]	Not described
Garrido 1 (2019) [26]	UK Brain Bank diagnostic criteria
Garrido 2 (2019) [26]	UK Brain Bank diagnostic criteria
Kang 1 (2019) [27]	UK Brain Bank diagnostic criteria
Kang 2 (2019) [27]	UK Brain Bank diagnostic criteria
Manne (2019) [28]	UK Brain Bank diagnostic criteria; NIH NeuroBioBank
Ning (2019) [29]	UK Brain Bank diagnostic criteria
van Rumund (2019) [30]	MDS clinical diagnostic criteria
Rossi (2020) [31]	MDS clinical diagnostic criteria
Shahnawaz (2020) [32]	UK Brain Bank diagnostic criteria
Singer (2020) [33]	MDS clinical diagnostic criteria
Bargar (2021) [34]	UK Brain Bank diagnostic criteria; NIH NeuroBioBank
Brockmann (2021) [35]	UK Brain Bank diagnostic criteria
Donadio (2021) [36]	Not described
Mammana (2021) [37]	MDS clinical diagnostic criteria
Orrù (2021) [38]	UK Brain Bank diagnostic criteria
Quadalti (2021) [39]	MDS clinical diagnostic criteria
Russo 1 (2021) [40]	Not described
Russo 2 (2021) [40]	Not described
Bongianni 1 (2022) [41]	MDS clinical diagnostic criteria
Bongianni 2 (2022) [41]	MDS clinical diagnostic criteria
Compta 1 (2022) [42]	MDS clinical diagnostic criteria
Compta 2 (2022) [42]	MDS clinical diagnostic criteria
Hall (2022) [43]	NINDS diagnostic criteria
Majbour (2022) [44]	UK Brain Bank diagnostic criteria
Sakurai (2022) [17]	MDS clinical diagnostic criteria
Poggiolini (2022) [45]	UK Brain Bank diagnostic criteria
Chahine (2023) [46]	No described
Concha-Marambio (2023) [47]	UK Brain Bank diagnostic criteria
Fernandes Gomes (2023) [48]	MDS clinical diagnostic criteria
Garrido (2023) [49]	Not described
Okuzumi (2023) [50]	MDS clinical diagnostic criteria
Siderowf (2023) [21]	Specified (Clinical diagnosis and DAT-SPECT)
Verdurand (2025) [51]	MDS clinical diagnostic criteria

Blood	
Okuzumi (2023) [50]	MDS clinical diagnostic criteria
Wang (2024) [52]	UK Brain Bank diagnostic criteria; NIH NeuroBioBank
Extracellular Vesicles	
Kluge (2023) [53]	UK Brain Bank diagnostic criteria
Kluge 1 (2024) [54]	Not described
Kluge 2 (2024) [54]	Not described
Schaeffer (2024) [55]	UK Brain Bank diagnostic criteria
Skin	
Manne 1 (2020) [56]	UK Brain Bank diagnostic criteria; NIH NeuroBioBank
Manne 2 (2020) [56]	UK Brain Bank diagnostic criteria; NIH NeuroBioBank
Wang 1 (2020) [57]	MDS clinical diagnostic criteria
Wang 2 (2020) [57]	MDS clinical diagnostic criteria
Donadio (2021) [36]	Not described
Kuzkina (2021) [58]	MDS clinical diagnostic criteria
Mammana (2021) [37]	Not described
Martinez-Valbuena (2022) [59]	Not described
Kuzkina (2023) [60]	MDS clinical diagnostic criteria
Kuang (2024) [61]	MDS clinical diagnostic criteria
Saliva	
Luan (2022) [62]	MDS clinical diagnostic criteria
Vivacqua (2023) [63]	Not described
Wang (2024) [52]	UK Brain Bank diagnostic criteria; NIH NeuroBioBank
Olfactory Mucosa	
De Luca (2019) [15]	MDS clinical diagnostic criteria
Bargar 1 (2021) [34]	UK Brain Bank diagnostic criteria; NIH NeuroBioBank
Bargar 2 (2021) [34]	UK Brain Bank diagnostic criteria; NIH NeuroBioBank
Stefani (2021) [64]	MDS clinical diagnostic criteria
Bongianni 1 (2022) [41]	MDS clinical diagnostic criteria
Bongianni 2 (2022) [41]	MDS clinical diagnostic criteria
Kuzkina (2023) [65]	MDS clinical diagnostic criteria
Oral Mucosa	
Zheng (2024) [66]	MDS clinical diagnostic criteria
Gastrointestinal Tract	
Fenyi (2019) [67]	UK Brain Bank diagnostic criteria
Fenyi (2021) [68]	Not described
Shin 1 (2022) [16]	Not described
Shin 2 (2022) [16]	Not described
Submandibular Gland	
Manne (2020) [69]	UK Brain Bank diagnostic criteria; NIH NeuroBioBank
Chahine (2023) [46]	Not described

S3. Diagnostic Results

Table. Diagnostic Results Of The Included Studies

Reference	PD; Controls	TP; FN; TN; FP	SN (95% CI)	SP (95% CI)	PLR (95% CI)	NLR (95% CI)	PPV (95% CI)	NPV (95% CI)	Accuracy (95% CI)
**Cerebrospinal Fluid**									
Fairfoul (2016) [22]	21; 35	20; 1; 35; 0	0.9524 (0.7618 to 0.9988)	1 (0.9 to 1)	NA	0.05 (0.01 to 0.32)	1 (0.8316 to 1)	0.9722 (0.8379 to 0.9958)	0.9821 (0.9045 to 0.9995)
Shahnawaz (2017) [23]	76; 65	67; 9; 63; 2	0.8816 (0.7871 to 0.9444)	0.9692 (0.8932 to 0.9963)	28.65 (7.3 to 112.41)	0.12 (0.07 to 0.23)	0.971 (0.8952 to 0.9924)	0.875 (0.791 to 0.9283)	0.922 (0.8647 to 0.9604)
Groveman (2018) [24]	12; 12	11; 1; 12; 0	0.9167 (0.6152 to 0.9979)	1 (0.7354 to 1)	NA	0.08 (0.01 to 0.54)	1 (0.7151 to 1)	0.9231 (0.6476 to 0.9874)	0.9583 (0.7888 to 0.9989)
Candelise (2019) [25]	10; 10	1; 9; 10; 0	0.1 (0.0025 to 0.445)	1 (0.6915 to 1)	NA	0.9 (0.73 to 1.11)	1 (0.025 to 1)	0.5263 (0.4747 to 0.5774)	0.55 (0.3153 to 0.7694)
Garrido 1 (2019) [26]	10; 10	9; 1; 8; 2	0.9 (0.555 to 0.9975)	0.8 (0.4439 to 0.9748)	4.5 (1.28 to 15.81)	0.12 (0.02 to 0.82)	0.8182 (0.5615 to 0.9405)	0.8889 (0.5484 to 0.9814)	0.85 (0.6211 to 0.9979)
Garrido 2 (2019) [26]	15; 10	6; 9; 8; 2	0.4 (0.1634 to 0.6771)	0.8 (0.4439 to 0.9748)	2 (0.5 to 8)	0.75 (0.45 to 1.23)	0.75 (0.4287 to 0.9231)	0.4706 (0.3465 to 0.5984)	0.56 (0.3493 to 0.756)
Kang 1 (2019) [27]	105; 79	100; 5; 65; 14	0.9524 (0.8924 to 0.9844)	0.8228 (0.7206 to 0.8996)	5.37 (3.34 to 8.66)	0.06 (0.02 to 0.14)	0.8772 (0.8159 to 0.9201)	0.9286 (0.846 to 0.9685)	0.8967 (0.8434 to 0.9367)
Kang 2 (2019) [27]	105; 79	102; 3; 73; 6	0.9714 (0.9188 to 0.9941)	0.9241 (0.842 to 0.9716)	12.79 (5.92 to 27.62)	0.03 (0.01 to 0.09)	0.9444 (0.8873 to 0.9735)	0.9605 (0.8884 to 0.9867)	0.9511 (0.9092 to 0.9774)
Manne (2019) [28]	15; 16	15; 0; 16; 0	1 (0.782 to 1)	1 (0.7941 to 1)	NA	0	1 (0.782 to 1)	1 (0.7941 to 1)	1 (0.8878 to 1)
Ning (2019) [29]	278; 278	237; 41; 254; 24	0.8525 (0.8053 to 0.892)	0.9137 (0.8743 to 0.9439)	9.87 (6.72 to 14.52)	0.16 (0.12 to 0.21)	0.908 (0.8704 to 0.9356)	0.861 (0.8233 to 0.8917)	0.8831 (0.8534 to 0.9086)
van Rumund (2019) [30]	51; 51	43; 8; 50; 1	0.8431 (0.7141 to 0.9298)	0.9804 (0.8955 to 0.9995)	43 (6.15 to 300.51)	0.16 (0.08 to 0.3)	0.9967 (0.8602 to 0.9773)	0.8621 (0.7677 to 0.922)	0.9118 (0.8391 to 0.9589)
Rossi (2020) [31]	71; 62	67; 4; 61; 1	0.9437 (0.862 to 0.9844)	0.9839 (0.9134 to 0.9996)	58.51 (8.37 to 409.16)	0.06 (0.02 to 0.15)	0.9853 (0.9055 to 0.9979)	0.9385 (0.8547 to 0.9753)	0.9624 (0.9144 to 0.9877)
Shahnawaz (2020) [32]	94; 56	88; 6; 56; 0	0.9362 (0.8662 to 0.9762)	1 (0.9362 to 1)	NA	0.06 (0.03 to 0.14)	1 (0.9589 to 1)	0.9032 (0.8114 to 0.9529)	0.96 (0.915 to 0.9852)
Singer (2020) [33]	16; 29	16; 0; 29; 0	1 (0.7941 to 1)	1 (0.8806 to 1)	NA	0	1 (0.7941 to 1)	1 (0.8806 to 1)	1 (0.9213 to 1)
Bargar (2021) [34]	88; 38	86; 2; 38; 0	0.9773 (0.9203 to 0.9972)	1 (0.9075 to 1)	NA	0.02 (0.01 to 0.09)	1 (0.958 to 1)	0.95 (0.8284 to 0.9868)	0.9841 (0.9438 to 0.9981)
Brockmann (2021) [35]	107; 26	91; 16; 24; 2	0.8505 (0.7686 to 0.912)	0.9231 (0.7487 to 0.9905)	11.06 (2.91 to 41.97)	0.16 (0.1 to 0.26)	0.9785 (0.923 to 0.9942)	0.6 (0.485 to 0.7049)	0.8647 (0.7946 to 0.9178)
Donadio (2021) [36]	2; 13	2; 0; 13; 0	1 (0.1581 to 1)	1 (0.7529 to 1)	NA	0	1 (0.1581 to 1)	1 (0.7529 to 1)	1 (0.782 to 1)
Mammana (2021) [37]	7; 27	7; 0; 27; 0	1 (0.5904 to 1)	1 (0.8723 to 1)	NA	0	1 (0.5904 to 1)	1 (0.8723 to 1)	1 (0.8972 to 1)
Orrù (2021) [38]	108; 85	105; 3; 74; 11	0.9722 (0.921 to 0.9942)	0.8706 (0.7802 to 0.9336)	7.51 (4.32 to 13.05)	0.03 (0.01 to 0.1)	0.9052 (0.846 to 0.9431)	0.961 (0.8896 to 0.9869)	0.9275 (0.8813 to 0.9598)
Quadalti (2021) [39]	153; 35	140; 13; 34; 1	0.915 (0.8591 to 0.954)	0.9714 (0.8508 to 0.9993)	32.03 (4.64 to 221.17)	0.09 (0.05 to 0.15)	0.9929 (0.953 to 0.999)	0.7234 (0.6079 to 0.8153)	0.9255 (0.8782 to 0.9587)
Russo 1 (2021) [40]	30; 28	26; 4; 27; 1	0.8667 (0.6928 to 0.9624)	0.9643 (0.8165 to 0.9991)	24.27 (3.52 to 167.15)	0.14 (0.06 to 0.35)	0.966 (0.7906 to 0.994)	0.871 (0.73 to 0.944)	0.9138 (0.8102 to 0.9714)
Russo 2 (2021) [40]	30; 28	29; 1; 28; 0	0.9667 (0.8278 to 0.9992)	1 (0.8766 to 1)	NA	0.03 (0 to 0.23)	1 (0.8806 to 1)	0.9655 (0.803 to 0.9948)	0.9828 (0.9076 to 0.9996)
Bongianni 1 (2022) [41]	23; 8	21; 2; 7; 1	0.913 (0.7196 to 0.9893)	0.875 (0.4735 to 0.9968)	7.3 (1.16 to 45.89)	0.1 (0.03 to 0.38)	0.9545 (0.7697 to 0.9925)	0.7778 (0.4757 to 0.931)	0.9032 (0.7425 to 0.9796)
Bongianni 2 (2022) [41]	1; 11	1; 0; 11; 0	1 (0.025 to 1)	1 (0.7151 to 1)	NA	0	1 (0.025 to 1)	1 (0.7151 to 1)	1 (0.7354 to 1)
Compta 1 (2022) [42]	20; 19	15; 5; 19; 0	0.75 (0.509 to 0.9134)	1 (0.8235 to 1)	NA	0.25 (0.12 to 0.53)	1 (0.782 to 1)	0.7917 (0.6401 to 0.8903)	0.8718 (0.7257 to 0.957)
Compta 2 (2022) [42]	20; 19	16; 4; 19; 0	0.8 (0.5634 to 0.9427)	1 (0.8235 to 1)	NA	0.2 (0.08 to 0.48)	1 (0.7941 to 1)	0.8261 (0.6641 to 0.9194)	0.8974 (0.7578 to 0.9713)
Hall (2022) [43]	20; 25	19; 1; 21; 4	0.95 (0.7513 to 0.9987)	0.84 (0.6392 to 0.9546)	5.94 (2.4 to 14.66)	0.06 (0.01 to 0.41)	0.8261 (0.658 to 0.9214)	0.9545 (0.7552 to 0.9931)	0.8889 (0.7595 to 0.9629)
Majbour (2022) [44]	62; 34	57; 5; 29; 5	0.9194 (0.8217 to 0.9733)	0.8529 (0.6894 to 0.9505)	6.25 (2.77 to 14.09)	0.09 (0.04 to 0.22)	0.9194 (0.8345 to 0.9625)	0.8529 (0.7122 to 0.9315)	0.8958 (0.8168 to 0.9489)
Sakurai (2022) [17]	13; 19	11; 2; 12; 7	0.8462 (0.5455 to 0.9808)	0.6316 (0.3836 to 0.8371)	2.3 (1.22 to 4.32)	0.24 (0.07 to 0.91)	0.6111 (0.4549 to 0.7474)	0.8571 (0.6157 to 0.9574)	0.7188 (0.5325 to 0.8625)
Poggiolini (2022) [45]	74; 55	66; 8; 53; 2	0.8919 (0.798 to 0.9522)	0.9636 (0.8747 to 0.9956)	24.53 (6.28 to 95.83)	0.11 (0.06 to 0.22)	0.9706 (0.8941 to 0.9923)	0.8689 (0.7746 to 0.9274)	0.9225 (0.8621 to 0.9622)
Chahine (2023) [46]	54; 21	50; 4; 19; 2	0.9259 (0.8211 to 0.9794)	0.7917 (0.5785 to 0.9287)	4.44 (2.03 to 9.73)	0.09 (0.04 to 0.25)	0.9091 (0.8204 to 0.9563)	0.8261 (0.6441 to 0.9258)	0.8846 (0.7922 to 0.9459)
Concha-Marambio (2023) [47]	71; 50	67; 4; 49; 1	0.9437 (0.862 to 0.9844)	0.98 (0.8935 to 0.9995)	47.18 (6.77 to 328.71)	0.06 (0.02 to 0.15)	0.9853 (0.9058 to 0.9979)	0.9245 (0.8253 to 0.9695)	0.9587 (0.9062 to 0.9864)
Fernandes Gomes (2023) [48]	55; 24	55; 0; 17; 7	1 (0.9351 to 1)	0.7083 (0.4891 to 0.8738)	3.43 (1.84 to 6.4)	0	0.8871 (0.8081 to 0.9361)	1 (0.8049 to 1)	0.9114 (0.8259 to 0.9636)
Garrido (2023) [49]	8; 3	7; 1; 3; 0	0.875 (0.4735 to 0.9968)	1 (0.2924 to 1)	NA	0.12 (0.02 to 0.78)	1 (0.5904 to 1)	0.75 (0.3241 to 0.9494)	0.9091 (0.5872 to 0.9977)
Okuzumi (2023) [50]	6; 35	6; 0; 35; 0	1 (0.5407 to 1)	1 (0.9 to 1)	NA	0	1 (0.5407 to 1)	1 (0.9 to 1)	1 (0.914 to 1)
Siderowf (2023) [21]	545; 163	478; 67; 157; 6	0.8771 (0.8465 to 0.9034)	0.9632 (0.9216 to 0.9864)	23.83 (10.86 to 52.29)	0.13 (0.1 to 0.16)	0.9876 (0.9732 to 0.9943)	0.7009 (0.6514 to 0.7461)	0.8969 (0.8721 to 0.9183)
Verdurand (2025) [51]	6; 37	6; 0; 37; 0	1 (0.5407 to 1)	1 (0.9051 to 1)	NA	0	1 (0.5407 to 1)	1 (0.9051 to 1)	1 (0.9178 to 1)

**Blood**								
Okuzumi (2023) [50]	221; 128	209; 12; 118; 10	0.9457 (0.9071 to 0.9716)	0.9219 (0.861 to 0.9619)	12.1 (6.67 to 21.97)	0.06 (0.03 to 0.1)	0.9543 (0.9201 to 0.9743)	0.9077 (0.8498 to 0.9447)	0.937 (0.9061 to 0.9601)
Wang (2024) [52]	82; 42	66; 16; 38; 4	0.8049 (0.7026 to 0.8842)	0.9048 (0.7738 to 0.9734)	8.45 (3.31 to 21.6)	0.22 (0.14 to 0.34)	0.9429 (0.8659 to 0.9768)	0.7037 (0.6022 to 0.7884)	0.8387 (0.7619 to 0.8986)
**Extracellular Vesicles**									
Kluge (2023) [53]	30; 50	30; 0; 50; 0	1 (0.8843 to 1)	1 (0.9289 to 1)	NA	0	1 (0.8843 to 1)	1 (0.9289 to 1)	1 (0.9549 to 1)
Kluge 1 (2024) [54]	13; 10	8; 5; 10; 0	0.6154 (0.3158 to 0.8614)	1 (0.6915 to 1)	NA	0.38 (0.19 to 0.76)	1 (0.6306 to 1)	0.6667 (0.5014 to 0.7991)	0.7826 (0.563 to 0.9254)
Kluge 2 (2024) [54]	9; 10	8; 1; 10; 0	0.8889 (0.5175 to 0.9972)	1 (0.6915 to 1)	NA	0.11 (0.02 to 0.71)	1 (0.6306 to 1)	0.9091 (0.6118 to 0.9845)	0.9474 (0.7397 to 0.9987)
Schaeffer (2024) [55]	80; 20	79; 1; 20; 0	0.9875 (0.9323 to 0.9997)	1 (0.8316 to 1)	NA	0.01 (0 to 0.09)	1 (0.9544 to 1)	0.9524 (0.7404 to 0.9929)	0.99 (0.9455 to 0.9997)
**Skin**									
Manne 1 (2020) [56]	25; 25	24; 1; 24; 1	0.96 (0.7965 to 0.999)	0.96 (0.7965 to 0.999)	24 (3.51 to 164.04)	0.04 (0.01 to 0.28)	0.96 (0.7783 to 0.9939)	0.96 (0.7783 to 0.9939)	0.96 (0.8629 to 0.9951)
Manne 2 (2020) [56]	12; 12	9; 3; 10; 2	0.75 (0.4281 to 0.9451)	0.8333 (0.5159 to 0.9791)	4.5 (1.22 to 16.62)	0.3 (0.11 to 0.83)	0.8182 (0.5492 to 0.9433)	0.7692 (0.5478 to 0.9017)	0.7917 (0.5785 to 0.9287)
Wang 1 (2020) [57]	47; 43	44; 3; 42; 1	0.9362 (0.8246 to 0.9866)	0.9767 (0.8771 to 0.9994)	40.26 (5.79 to 279.71)	0.07 (0.02 to 0.2)	0.9778 (0.8636 to 0.9967)	0.9333 (0.8239 to 0.9767)	0.9556 (0.8901 to 0.9878)
Wang 2 (2020) [57]	20; 21	19; 1; 20; 1	0.95 (0.7513 to 0.9987)	0.9524 (0.7618 to 0.9988)	19.95 (2.94 to 135.45)	0.05 (0.01 to 0.36)	0.95 (0.7367 to 0.9923)	0.9524 (0.7471 to 0.9927)	0.9512 (0.8347 to 0.994)
Donadio (2021) [36]	6; 18	5; 1; 14; 4	0.8333 (0.3588 to 0.9958)	0.7778 (0.5236 to 0.9359)	3.75 (1.47 to 9.56)	0.21 (0.04 to 1.3)	0.5556 (0.3291 to 0.7611)	0.9333 (0.697 to 0.9884)	0.7917 (0.5785 to 0.9287)
Kuzkina (2021) [58]	34; 30	31; 3; 27; 3	0.9118 (0.7632 to 0.9814)	0.9 (0.7347 to 0.9789)	9.12 (3.1 to 26.81)	0.1 (0.03 to 0.29)	0.9118 (0.7785 to 0.9681)	0.9 (0.7522 to 0.9639)	0.9062 (0.807 to 0.9648)
Mammana (2021) [37]	13; 41	10; 3; 39; 2	0.7692 (0.4619 to 0.9496)	0.9512 (0.8347 to 0.994)	15.77 (3.95 to 62.94)	0.24 (0.09 to 0.66)	0.8333 (0.5561 to 0.9523)	0.9286 (0.8278 to 0.9723)	0.9074 (0.797 to 0.9692)
Martinez-Valbuena (2022) [59]	13; 20	11; 2; 17; 3	0.8462 (0.5455 to 0.9808)	0.85 (0.6211 to 0.9679)	5.64 (1.94 to 16.42)	0.18 (0.05 to 0.66)	0.7857 (0.5574 to 0.9144)	0.8947 (0.701 to 0.9686)	0.8485 (0.681 to 0.9489)
Kuzkina (2023) [60]	34; 30	30; 4; 26; 4	0.8824 (0.7255 to 0.967)	0.8667 (0.6928 to 0.9624)	6.62 (2.64 to 16.61)	0.14 (0.05 to 0.34)	0.8824 (0.7492 to 0.9496)	0.8667 (0.7192 to 0.9428)	0.875 (0.7685 to 0.9445)
Kuang (2024) [61]	332; 285	307; 25; 266; 19	0.9247 (0.8908 to 0.9507)	0.9333 (0.8978 to 0.9594)	13.87 (8.97 to 21.44)	0.08 (0.06 to 0.12)	0.9417 (0.9127 to 0.9615)	0.9141 (0.8794 to 0.9395)	0.9287 (0.9054 to 0.9477)
**Saliva**									
Luan (2022) [62]	75; 36	57; 18; 34; 2	0.76 (0.6475 to 0.8511)	0.9444 (0.8134 to 0.9932)	13.68 (3.54 to 52.92)	0.25 (0.17 to 0.38)	0.9661 (0.8805 to 0.991)	0.6538 (0.5561 to 0.7404)	0.8198 (0.7355 to 0.8863)
Vivacqua (2023) [63]	37; 23	31; 6; 19; 4	0.8378 (0.6799 to 0.9381)	0.8261 (0.6122 to 0.9505)	4.82 (1.95 to 11.87)	0.2 (0.09 to 0.42)	0.8857 (0.7587 to 0.9502)	0.76 (0.5979 to 0.8709)	0.8333 (0.7148 to 0.9171)
Wang (2024) [52]	48; 26	36; 12; 25; 1	0.75 (0.604 to 0.8636)	0.9615 (0.8036 to 0.999)	19.5 (2.83 to 134.19)	0.26 (0.16 to 0.43)	0.973 (0.8395 to 0.996)	0.6757 (0.5592 to 0.7738)	0.8243 (0.7183 to 0.903)
**Olfactory Mucosa**									
De Luca (2019) [15]	18; 0	18; 10; NA; NA	0.5555 (0.5555 to 0.5555)	NA	NA	NA		NA	
Bargar 1 (2021) [34]	13; 11	9; 4; 10; 1	0.6923 (0.3857 to 0.9091)	0.9091 (0.5872 to 0.9977)	7.62 (1.13 to 51.1)	0.34 (0.15 to 0.78)	0.9 (0.5729 to 0.9837)	0.7143 (0.5199 to 0.8523)	0.7917 (0.5785 to 0.9287)
Bargar 2 (2021) [34]	13; 11	9; 4; 11; 0	0.6923 (0.3857 to 0.9091)	1 (0.7151 to 1)	NA	0.31 (0.14 to 0.7)	1 (0.6637 to 1)	0.7333 (0.5489 to 0.8614)	0.8333 (0.6262 to 0.9526)
Stefani (2021) [64]	41; 59	19; 22; 53; 6	0.4634 (0.3066 to 0.6258)	0.8983 (0.7917 to 0.9618)	4.56 (1.99 to 10.42)	0.6 (0.44 to 0.8)	0.76 (0.5807 to 0.8786)	0.7067 (0.6416 to 0.7643)	0.72 (0.6213 to 0.8052)
Bongianni 1 (2022) [41]	43; 29	19; 24; 19; 10	0.4419 (0.2908 to 0.6012)	0.6552 (0.4567 to 0.8206)	1.28 (0.7 to 2.34)	0.85 (0.59 to 1.24)	0.6552 (0.5095 to 0.7765)	0.4419 (0.3524 to 0.5352)	0.5278 (0.4065 to 0.6467)
Bongianni 2 (2022) [41]	43; 29	36; 7; 19; 10	0.8372 (0.693 to 0.9319)	0.6552 (0.4567 to 0.8206)	2.43 (1.45 to 4.08)	0.25 (0.12 to 0.51)	0.7826 (0.6818 to 0.8581)	0.7308 (0.5674 to 0.8489)	0.7639 (0.6491 to 0.856)
Kuzkina (2023) [65]	27; 30	13; 14; 27; 3	0.4815 (0.2867 to 0.6805)	0.9 (0.7347 to 0.9789)	4.81 (1.54 to 15.1)	0.58 (0.39 to 0.84)	0.8125 (0.5802 to 0.9314)	0.6585 (0.5681 to 0.7387)	0.7018 (0.566 to 0.8157)
**Oral Mucosa**									
Zheng (2024) [66]	107; 103	72; 35; 93; 10	0.6729 (0.5755 to 0.7605)	0.9029 (0.8287 to 0.9525)	6.93 (3.79 to 12.67)	0.36 (0.27 to 0.48)	0.878 (0.7975 to 0.9294)	0.7266 (0.6678 to 0.7784)	0.7857 (0.724 to 0.8392)
**Gastrointestinal Tract**									
Fenyi (2019) [67]	18; 11	10; 8; 10; 1	0.5556 (0.3076 to 0.7847)	0.9091 (0.5872 to 0.9977)	6.11 (0.9 to 41.43)	0.49 (0.28 to 0.85)	0.9091 (0.596 to 0.9855)	0.5556 (0.4192 to 0.684)	0.6897 (0.4917 to 0.8472)
Fenyi (2021) [68]	12; 9	10; 2; 7; 2	0.8333 (0.5159 to 0.9791)	0.7778 (0.3999 to 0.9719)	3.75 (1.08 to 13.07)	0.21 (0.06 to 0.8)	0.8333 (0.5893 to 0.9457)	0.7778 (0.4851 to 0.9286)	0.8095 (0.5809 to 0.9455)
Shin 1 (2022) [16]	2; 20	2; 0; NA; NA	1 (1 to 1)	NA	NA	NA	NA	NA	NA
Shin 2 (2022) [16]	20; 20	2; 18; 17; 3	0.1 (0.0123 to 0.317)	0.85 (0.6211 to 0.9679)	0.67 (0.12 to 3.57)	1.06 (0.84 to 1.34)	0.4 (0.1107 to 0.7813)	0.4857 (0.4275 to 0.5444)	0.475 (0.3151 to 0.6387)
**Submandibular Gland**									
Manne (2020) [69]	13; 16	13; 0; 15; 1	1 (0.7529 to 1)	0.9375 (0.6977 to 0.9984)	16 (2.4 to 106.74)	0	0.9286 (0.6609 to 0.9886)	1 (0.782 to 1)	0.9655 (0.8224 to 0.9991)
Chahine (2023) [46]	41; 14	30; 11; 11; 3	0.7317 (0.5706 to 0.8578)	0.7857 (0.492 to 0.9534)	3.41 (1.23 to 9.47)	0.34 (0.19 to 0.61)	0.9091 (0.7829 to 0.9652)	0.5 (0.3601 to 0.6399)	0.7455 (0.61 to 0.8533)

Table. Positive And Negative Likelihood Ratio of biomatrices for diagnosing Parkinson's disease		
Biomatrices	Positive Likelihood Ratio	Negative Likelihood Ratio
Cerebrospinal fluid	14.07 (95% CI, 11.65–16.98)	0.11 (95% CI, 0.09–0.12)
Blood	11.02 (95% CI, 6.66–18.23)	0.10 (95% CI, 0.07–0.14)
Extracellular vesicles	∞ (infinity)	0.05 (95% CI, 0.03–0.11)
Skin	12.00 (95% CI, 8.90–16.18)	0.09 (95% CI, 0.07–0.12)
Saliva	9.41 (95% CI, 4.61–19.23)	0.25 (95% CI, 0.18–0.33)
Olfactory	3.29 (95% CI, 2.32–4.65)	0.51 (95% CI, 0.42–0.61)
Oral	6.93 (95% CI, 3.79–12.67)	0.36 (95% CI, 0.27–0.48)
Gastrointestinal tract	2.93 (95% CI, 1.32–6.54)	0.66 (95% CI, 0.50–0.87)
Submandibular gland	5.97 (95% CI, 2.37–15.02)	0.24 (95% CI, 0.14–0.41)
All[a] except CSF	8.98 (95% CI, 7.51–10.73)	0.20 (95% CI, 0.18–0.22)
All[a] including CSF	11.45 (95% CI, 10.06–13.04)	0.14 (95% CI, 0.13–0.15)

Abbreviations: CI, confidence interval.

[a] All biomatracies are CSF, blood, extracellular vesicles, skin, saliva, olfactory, oral, gastronintestinal tract, and submandibular gland.

S4. Quality

Table. Quality Assessment Results							
	Risk of Bias				Applicability Concerns		
**Study**	**D1**	**D2**	**D3**	**D4**	**D1**	**D2**	**D3**
**Cerebrospinal Fluid**							
Fairfoul (2016) [22]	No information	High	Low	No information	Low	Low	Low
Shahnawaz (2017) [23]	No information	High	Low	No information	Low	No information	Low
Groveman (2018) [24]	No information	Low	High	No information	No information	Low	Low
Candelise (2019) [25]	No information	Low	High	No information	No information	Low	Low
Garrido 1 (2019) [26]	No information	Low	No information	No information	Low	High	Low
Garrido 2 (2019) [26]	No information	Low	No information	No information	Low	High	Low
Kang 1 (2019) [27]	Low	Low	Low	Low	Low	Low	Low
Kang 2 (2019) [27]	Low	Low	Low	Low	Low	Low	Low
Manne (2019) [28]	No information	Low	High	High	High	No information	High
Ning (2019) [29]	High	High	Low	High	No information	High	Low
van Rumund (2019) [30]	No information	No information	Low	No information	Low	Low	Low
Rossi (2020) [31]	No information	Low	Low	High	Low	Low	Low
Shahnawaz (2020) [32]	No information	High	Low	High	Low	High	Low
Singer (2020) [33]	Low	High	No information	No information	High	No information	High
Bargar (2021) [34]	No information	Low	Low	Low	Low	Low	Low
Brockmann (2021) [35]	No information	Low	Low	No information	No information	No information	Low
Donadio (2021) [36]	Low	Low	No information	No information	Low	Low	No information
Mammana (2021) [37]	No information	Low	Low	High	High	No information	No information
Orrù (2021) [38]	Low	Low	Low	No information	Low	Low	Low
Quadalti (2021) [39]	No information	Low	Low	Low	Low	Low	Low
Russo 1 (2021) [40]	No information	Low	High	High	No information	Low	Low
Russo 2 (2021) [40]	No information	Low	High	High	No information	Low	Low
Bongianni 1 (2022) [41]	Low	Low	Low	Low	Low	Low	Low
Bongianni 2 (2022) [41]	Low	Low	Low	Low	Low	Low	No information
Compta 1 (2022) [42]	No information	Low	No information	Low	Low	Low	Low
Compta 2 (2022) [42]	No information	Low	Low	Low	Low	Low	Low
Hall (2022) [43]	No information	Low	Low	High	Low	High	High
Majbour (2022) [44]	Low	No information	Low	Low	Low	Low	Low
Sakurai (2022) [17]	Low	Low	Low	Low	Low	Low	Low
Poggiolini (2022) [45]	No information	Low	Low	High	Low	Low	Low
Chahine (2023) [46]	Low	Low	High	Low	No information	Low	Low
Concha-Marambio (2023) [47]	Low	Low	Low	Low	Low	Low	High
Fernandes Gomes (2023) [48]	No information	Low	Low	Low	Low	Low	Low
Garrido (2023) [49]	Low	Low	No information	Low	Low	Low	Low
Okuzumi (2023) [50]	No information	Low	Low	Low	Low	Low	Low
Siderowf (2023) [21]	Low	Low	Low	Low	Low	Low	No information

Verdurand (2025) [51]	Low	Low	High	Low	Low	Low	Low
**Blood**							
Okuzumi (2023) [50]	Low	Low	Low	Low	Low	Low	Low
Wang (2024) [52]	Low	Low	Low	Low	Low	Low	Low
**Extracellular Vesicles**							
Kluge (2023) [53]	Low	Low	Low	Low	Low	Low	Low
Kluge 1 (2024) [54]	Low	High	Low	Low	Low	No information	Low
Kluge 2 (2024) [54]	Low	Low	Low	Low	Low	Low	Low
Schaeffer (2024) [55]	Low	Low	Low	Low	Low	Low	Low
**Skin**							
Manne 1 (2020) [56]	Low	Low	High	Low	Low	Low	Low
Manne 2 (2020) [56]	Low	Low	Low	Low	Low	Low	Low
Wang 1 (2020) [57]	High	Low	Low	Low	Low	Low	Low
Wang 2 (2020) [57]	No information	Low	No information	Low	Low	Low	No information
Donadio (2021) [36]	Low	Low	Low	Low	No information	Low	Low
Kuzkina (2021) [58]	Low	Low	High	Low	Low	Low	Low
Mammana (2021) [37]	Low	High	Low	Low	Low	Low	Low
Martinez-Valbuena (2022) [59]	Low	Low	Low	No information	Low	No information	Low
Kuzkina (2023) [60]	Low	Low	Low	Low	Low	Low	Low
Kuang (2024) [61]	Low	Low	Low	Low	Low	Low	Low
**Saliva**							
Luan (2022) [62]	Low	Low	Low	Low	Low	Low	Low
Vivacqua (2023) [63]	Low	No information	High	Low	No information	Low	Low
Wang (2024) [52]	High	Low	Low	Low	Low	Low	Low
**Olfactory Mucosa**							
De Luca (2019) [15]	Low	Low	Low	Low	Low	Low	Low
Bargar 1 (2021) [34]	Low	Low	Low	Low	Low	Low	Low
Bargar 2 (2021) [34]	Low	No information	Low	Low	No information	Low	Low
Stefani (2021) [64]	Low	Low	Low	Low	Low	Low	Low
Bongianni 1 (2022) [41]	No information	Low	High	Low	Low	No information	No information
Bongianni 2 (2022) [41]	Low	Low	No information	Low	Low	Low	Low
Kuzkina (2023) [65]	High	Low	Low	Low	Low	Low	Low
**Oral Mucosa**							
Zheng (2024) [66]	Low	Low	Low	Low	Low	Low	Low
**Gastrointestinal Tract**							
Fenyi (2019) [67]	No information	Low	Low	Low	Low	Low	Low
Fenyi (2021) [68]	Low	High	Low	No information	No information	Low	Low
Shin 1 (2022) [16]	Low	No information	High	Low	Low	Low	Low
Shin 2 (2022) [16]	Low	Low	Low	Low	Low	High	Low
**Submandibular Gland**							
Manne (2020) [69]	Low	No information	Low	Low	Low	Low	No information
Chahine (2023) [46]	Low	Low	Low	Low	No information	Low	Low

Abbreviations: D1, patient selection; D2, index test; D3, reference standard; D4, flow and timing.

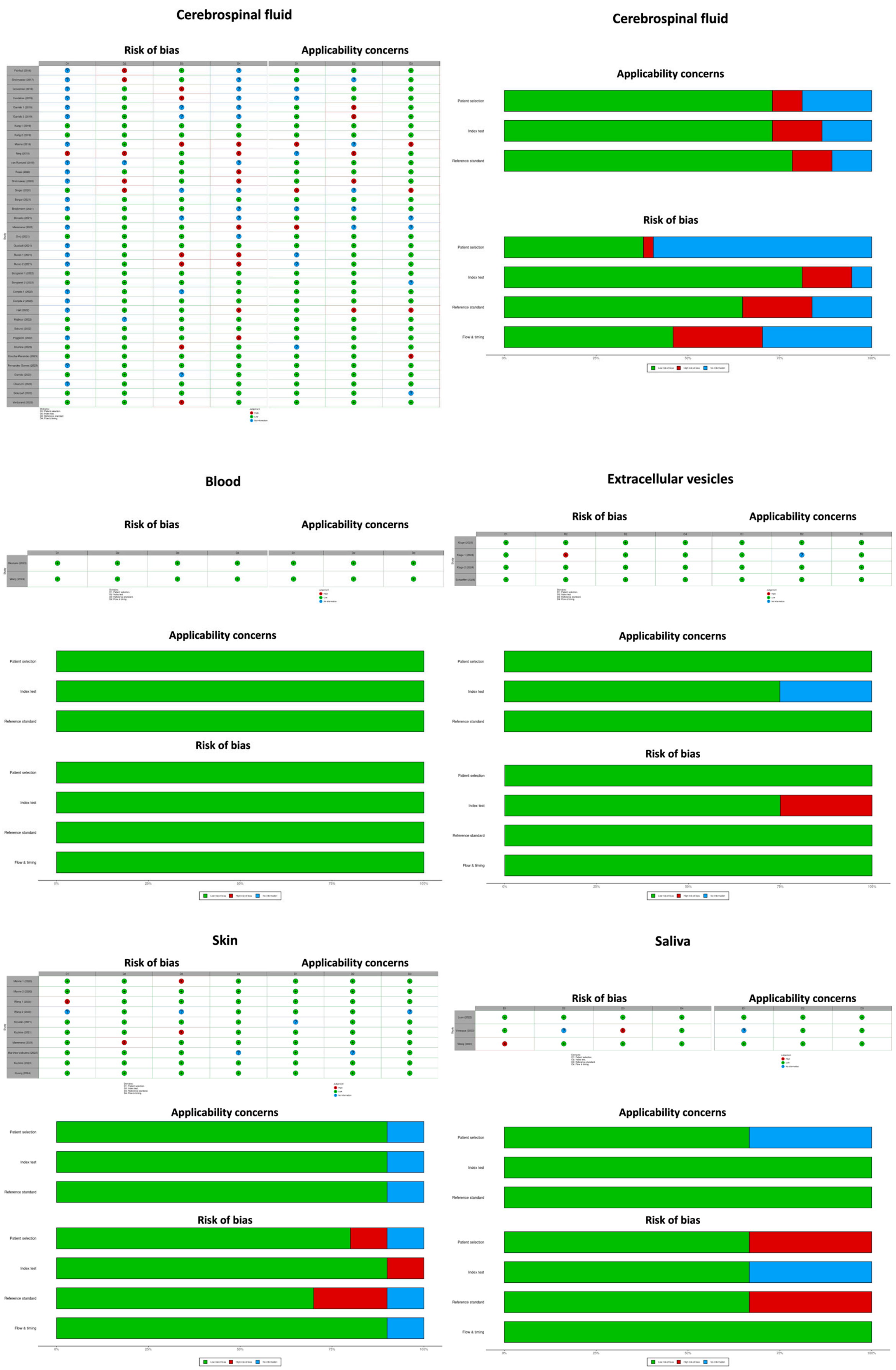

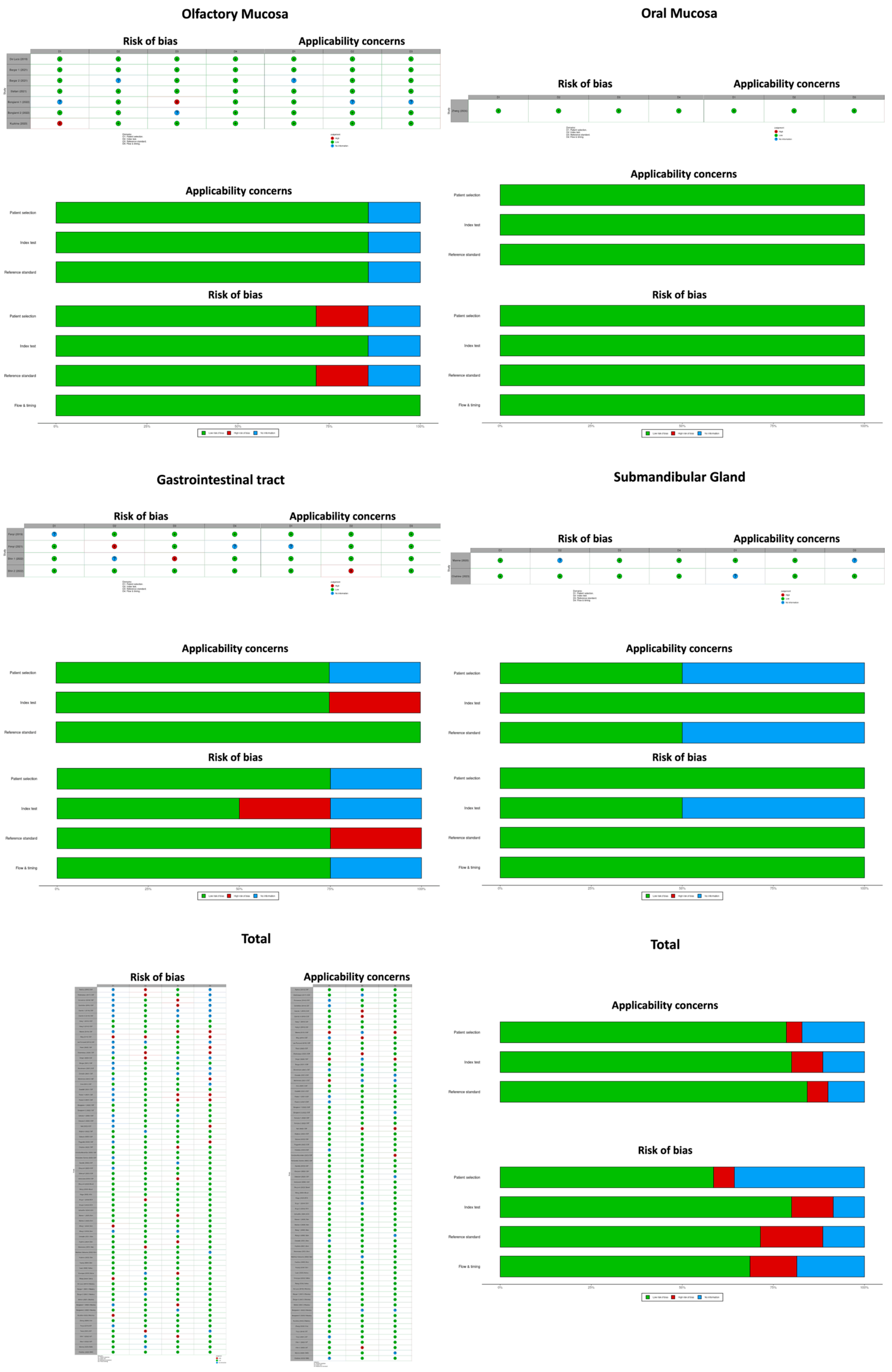
Olfactory Mucosa
Risk of bias
Applicability concerns
Applicability concerns
Risk of bias
Oral Mucosa
Risk of bias
Applicability concerns
Applicability concerns
Risk of bias
Gastrointestinal tract
Risk of bias
Applicability concerns
Applicability concerns
Risk of bias
Submandibular Gland
Risk of bias
Applicability concerns
Applicability concerns
Risk of bias
Total
Risk of bias
Applicability concerns
Total
Applicability concerns
Risk of bias

S5. Forest Plot

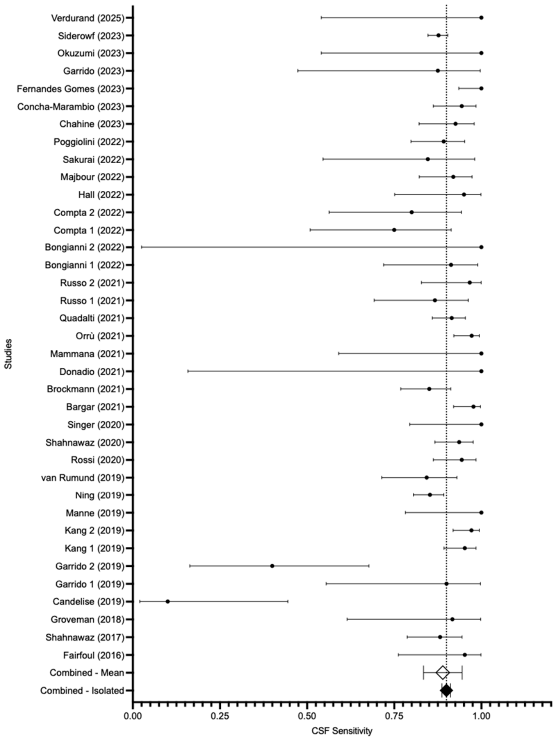

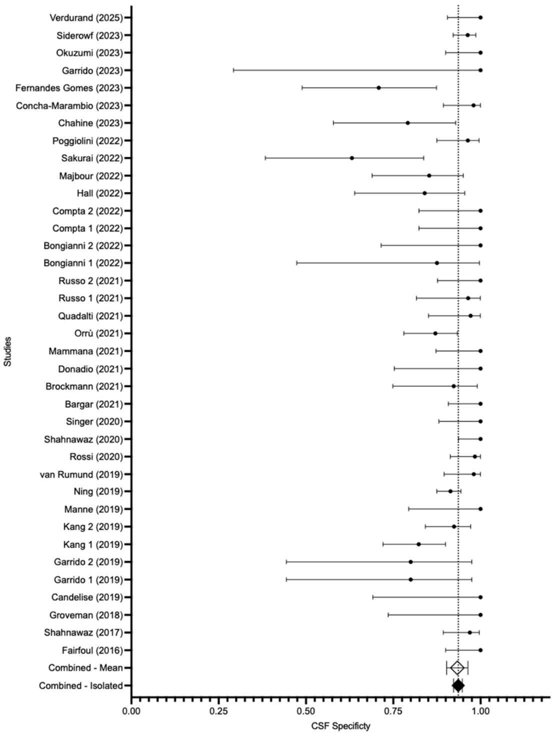

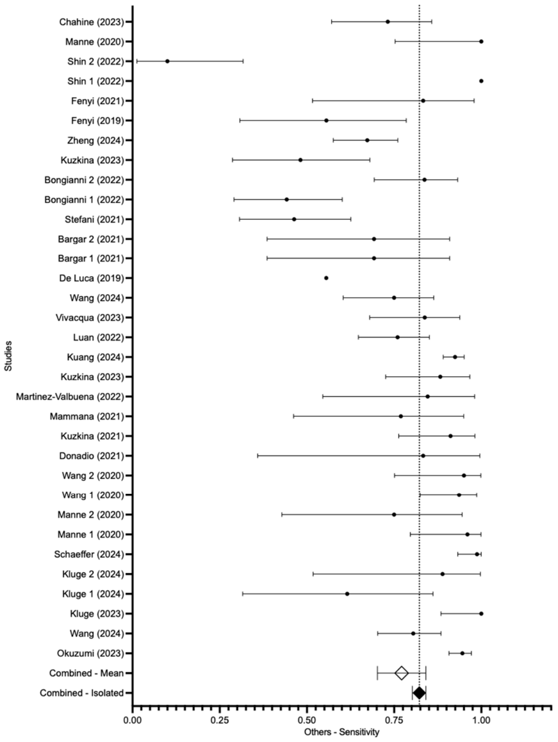

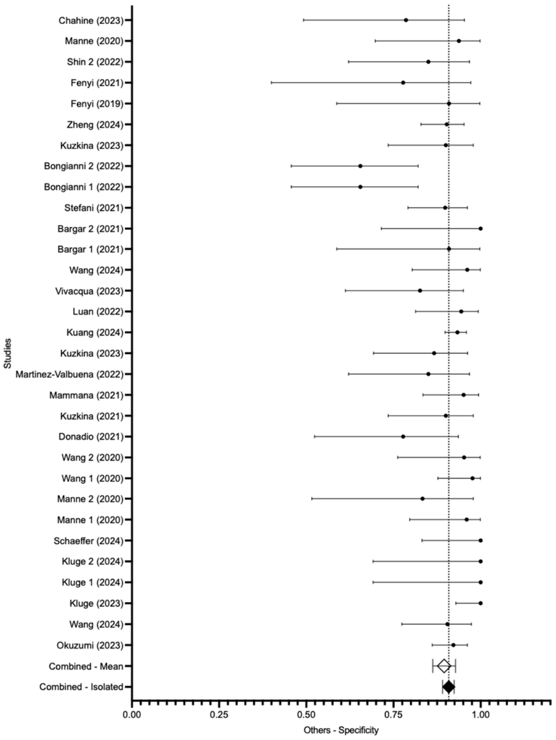

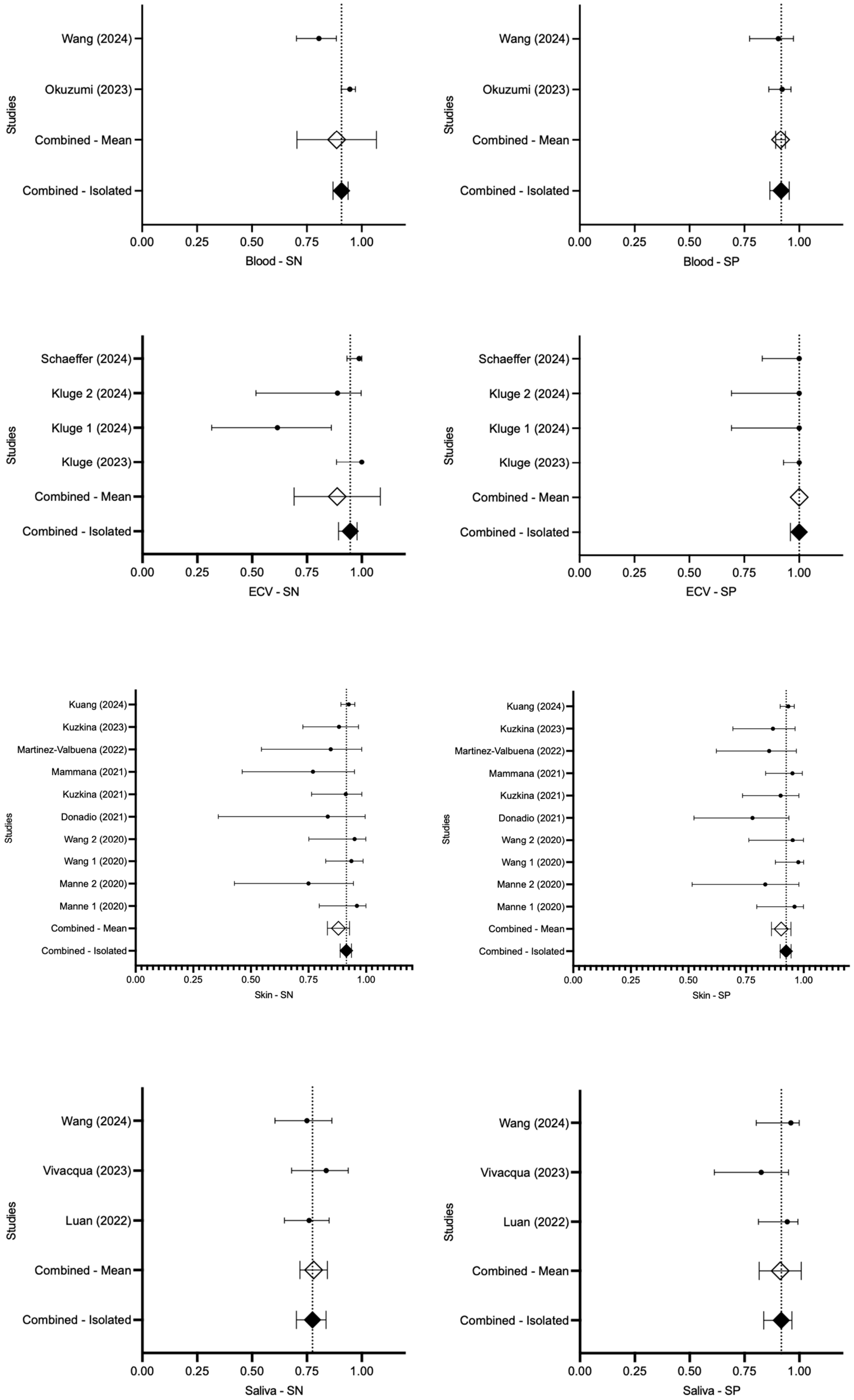
Studies
Wang (2024)
Okuzumi (2023)
Combined - Mean
Combined - Isolated
0.00 0.25 0.50 0.75 1.00
Blood - SN
Blood - SP
Schaeffer (2024)
Kluge 2 (2024)
Kluge 1 (2024)
Kluge (2023)
ECV - SN
ECV - SP
Kuang (2024)
Kuzkina (2023)
Martinez-Valbuena (2022)
Mammana (2021)
Kuzkina (2021)
Donadio (2021)
Wang 2 (2020)
Wang 1 (2020)
Manne 2 (2020)
Manne 1 (2020)
Skin - SN
Skin - SP
Vivacqua (2023)
Luan (2022)
Saliva - SN
Saliva - SP

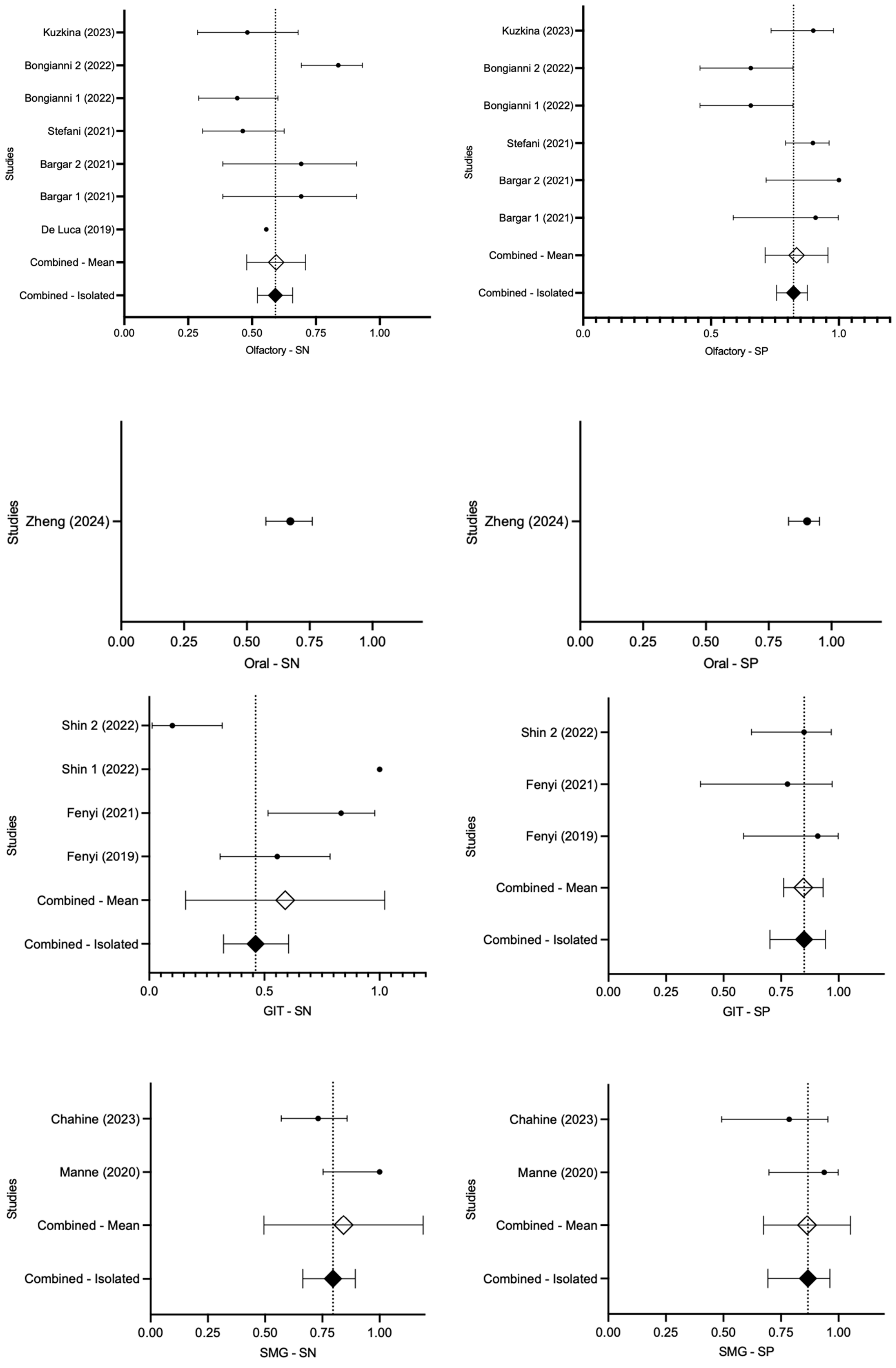
Kuzkina (2023)
Bongianni 2 (2022)
Bongianni 1 (2022)
Stefani (2021)
Bargar 2 (2021)
Bargar 1 (2021)
De Luca (2019)
Combined - Mean
Combined - Isolated
Studies
0.00
0.25
0.50
0.75
1.00
Olfactory - SN
Kuzkina (2023)
Bongianni 2 (2022)
Bongianni 1 (2022)
Stefani (2021)
Bargar 2 (2021)
Bargar 1 (2021)
Combined - Mean
Combined - Isolated
Studies
0.0
0.5
1.0
Olfactory - SP
Zheng (2024)
Studies
0.00
0.25
0.50
0.75
1.00
Oral - SN
Zheng (2024)
Studies
0.00
0.25
0.50
0.75
1.00
Oral - SP
Shin 2 (2022)
Shin 1 (2022)
Fenyi (2021)
Fenyi (2019)
Combined - Mean
Combined - Isolated
Studies
0.0
0.5
1.0
GIT - SN
Shin 2 (2022)
Fenyi (2021)
Fenyi (2019)
Combined - Mean
Combined - Isolated
Studies
0.00
0.25
0.50
0.75
1.00
GIT - SP
Chahine (2023)
Manne (2020)
Combined - Mean
Combined - Isolated
Studies
0.00
0.25
0.50
0.75
1.00
SMG - SN
Chahine (2023)
Manne (2020)
Combined - Mean
Combined - Isolated
Studies
0.00
0.25
0.50
0.75
1.00
SMG - SP

S6. Random Effect

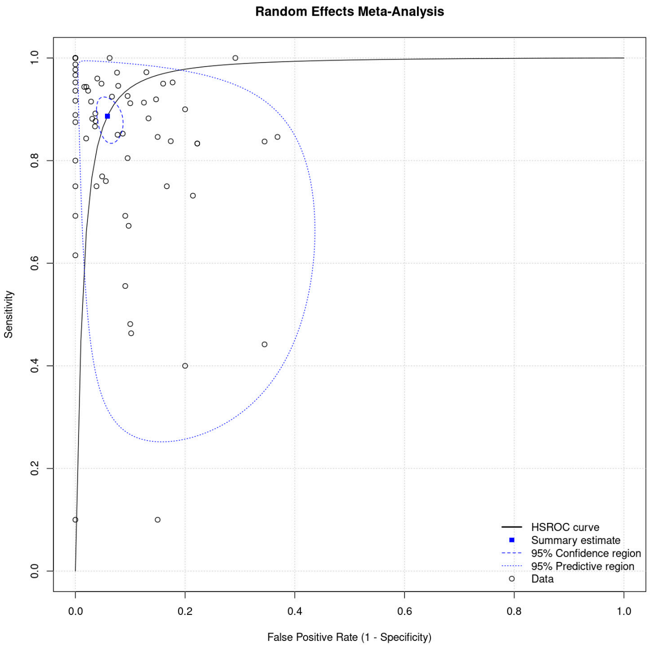

Random effect meta-analysis graph with summary receiver operating characteristic curve analysis.

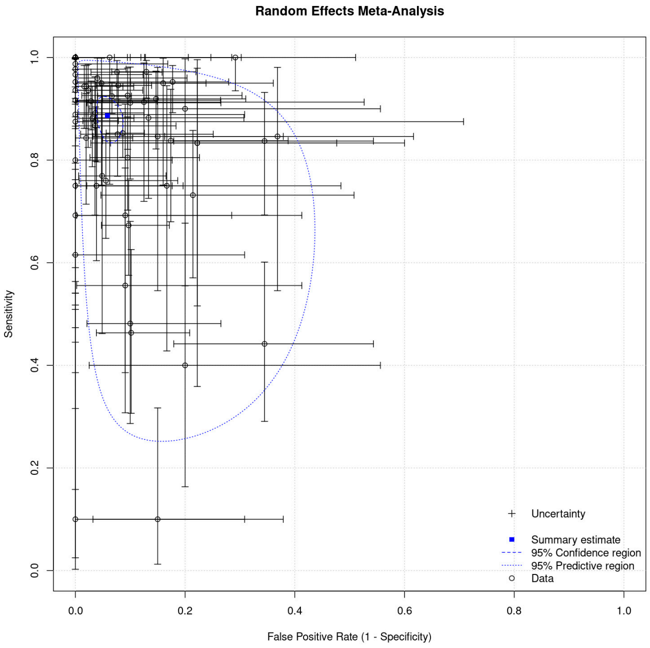

Random effect meta-analysis displaying 95% study level confidence intervals of sensitivity and specificity.

S7. Difference

The Z-test for comparing two proportions is used to test if there is a significant difference between the proportions in two groups. The formula for the Z-test statistic is:

$$Z = \frac{(p_1 - p_2)}{\sqrt{\hat{p}(1-\hat{p})\left(\frac{1}{n_1} + \frac{1}{n_2}\right)}}$$

Where:

- $p1$ and $p2$ are the sample proportions from each group.

- $n1$ and $n2$ are the sample sizes from each group.

- $\hat{p}$ is the pooled proportion, which is calculated as:

$$\hat{p} = \frac{x_1 + x_2}{n_1 + n_2}$$

- where $x1$ and $x2$ are the number of successes in each group.

The Wald Confidence Interval (CI) for the difference between two proportions can be calculated using the following formula:

$$\text{CI} = (p_1 - p_2) \pm Z \times \sqrt{\frac{p_1(1-p_1)}{n_1} + \frac{p_2(1-p_2)}{n_2}}$$

Where:

- $p1$ and $p2$ are the proportions (for example, sensitivities or specificities) for the two groups

- $n1$ and $n2$ are the sample sizes for the two groups

- Z is the critical value corresponding to the desired confidence level (e.g., for 95% confidence, $Z \approx 1.96$)

Table. Baseline data			
Biomatrice	n	Sensitivity	Specificity
CSF	2382	0.89 (95% CI, 0.88–0.91)	0.93 (95% CI, 0.92–0.94)
Blood	303	0.90 (95% CI, 0.86–0.93)	0.91 (95% CI, 0.86–0.95)
Extracellular vesicles	132	0.94 (95% CI, 0.89–0.97)	1.00 (95% CI, 0.95–1.00)
Skin	536	0.91 (95% CI, 0.88–0.93)	0.92 (95% CI, 0.89–0.94)
Saliva	160	0.77 (95% CI, 0.70–0.83)	0.91 (95% CI, 0.83–0.96)
Olfactory	198	0.59 (95% CI, 0.52–0.65)	0.82 (95% CI, 0.75–0.87)
Oral	107	0.67 (95% CI, 0.57–0.76)	0.90 (95% CI, 0.82–0.95)

Gastrointestinal tract	52	0.46 (95% CI, 0.32–0.60)	0.85 (95% CI, 0.70–0.94)
Submandibular gland	54	0.79 (95% CI, 0.66–0.89)	0.86 (95% CI, 0.69–0.96)

Table. Comparison table of sensitivity with single-population analysis with Z-test

Biomatrice	CSF	Blood	ECV	Skin	Saliva	Olfactory	Oral	GIT	SMG
CSF	0	-0.52	-1.80	-1.35	4.56*	11.94*	6.86*	9.46*	2.30*
Blood	0.52	0	-1.35	-0.46	3.77*	8.15*	5.57*	7.90*	2.32*
ECV	1.80	1.35	0	1.11	4.01*	7.01*	5.39*	7.39*	3.04*
Skin	1.35	0.46	-1.11	0	4.74*	10.09*	6.74*	9.21*	2.78*
Saliva	-4.56*	-3.77*	-4.01*	-4.74*	0	3.60*	1.80	4.21*	-0.30
Olfactory	-11.94*	-8.15*	-7.01*	-10.09*	-3.60*	0	-1.37	1.68	-2.70*
Oral	-6.86*	-5.57*	-5.39*	-6.74*	-1.80	1.37	0	2.53*	-1.58
GIT	-9.46*	-7.90*	-7.39*	-9.21*	-4.21*	-1.68	-2.53*	0	-3.51*
SMG	-2.30*	-2.32*	-3.04*	-2.78*	0.30	2.70*	1.58	3.51*	0

Summary of sensitivity scale: (CSF, blood, ECV, Skin) > (saliva, oral, SMG) > (olfactory, GIT)

*, if p-value < 0.05

Table. Comparison table of sensitivity with single-population analysis with Wald Confidence Interval

Bioma-trice	CSF	Blood	ECV	Skin	Saliva	Olfactory	Oral	GIT	SMG
CSF	X	(-0.04, 0.02)	(-0.08, -0.01)*	(-0.04, 0.01)	(0.05, 0.18)*	(0.23, 0.36)*	(0.12, 0.31)*	(0.28, 0.57)*	(-0.01, 0.20)
Blood	(-0.02, 0.04)	X	(-0.01, 0.08)	(-0.02, 0.04)	(0.06, 0.19)*	(0.24, 0.37)*	(0.13, 0.32)*	(0.29, 0.58)*	(0.01, 0.21)*
ECV	(0.01, 0.08)*	(-0.08, 0.01)	X	(-0.01, 0.06)	(0.10, 0.23)*	(0.28, 0.41)*	(0.17, 0.36)*	(0.33, 0.62)*	(0.04, 0.25)*
Skin	(-0.01, 0.04)	(-0.04, 0.02)	(-0.06, 0.01)	X	(0.07, 0.20)*	(0.25, 0.38)*	(0.14, 0.33)*	(0.30, 0.59)*	(0.01, 0.22)*
Saliva	(-0.18, -0.05)*	(-0.19, -0.06)*	(-0.23, -0.10)*	(-0.20, -0.07)*	X	(0.09, 0.26)*	(-0.01, 0.20)	(0.15, 0.46)*	(-0.09, 0.13)
Olfactory	(-0.36, -0.23)*	(-0.37, -0.24)*	(-0.41, -0.28)*	(-0.38, -0.25)*	(-0.26, -0.09)*	X	(-0.02, 0.18)	(-0.02, 0.28)	(0.08, 0.31)*
Oral	(-0.31, -0.12)*	(-0.32, -0.13)*	(-0.36, -0.17)*	(-0.33, -0.14)*	(-0.20, 0.01)	(-0.18, 0.02)	X	(0.04, 0.37)*	(-0.01, 0.25)

GIT	(-0.57, -0.28)*	(-0.58, -0.29)*	(-0.62, -0.33)*	(-0.59, -0.30)*	(-0.46, -0.15)*	(-0.28, 0.02)	(-0.37, -0.04)*	X	(0.15, 0.50)*
SMG	(-0.20, 0.01)	(-0.21, -0.01)*	(-0.25, -0.04)*	(-0.22, -0.01)*	(-0.13, 0.09)	(-0.31, -0.08)*	(-0.25, 0.01)	(-0.50, -0.15)*	X

Summary of sensitivity scale: (ECV) ≥ (CSF, blood, skin) > (saliva) ≥ (GIT, olfactory) ≥ (oral, SMG)

*, if the result is significant (not including zero)

Table. Comparison table of specificity with single-population analysis with Z-test

Biomatrice	CSF	Blood	ECV	Skin	Saliva	Olfactory	Oral	GIT	SMG
CSF	0	1.26	-3.14*	0.81	0.95	5.53*	1.17	2.21*	1.97*
Blood	-1.26	0	-3.56*	-0.50	0	2.97*	0.30	1.33	1.14
ECV	3.14*	3.56*	0	3.35*	3.53*	5.16*	3.71*	4.54*	4.38*
Skin	-0.81	0.50	-3.35*	0	0.40	3.89*	0.68	1.71	1.50
Saliva	-0.95	0	-3.53*	-0.40	0	2.44*	0.27	1.22	1.04
Olfactory	-5.53*	-2.97*	-5.16*	-3.89*	-2.44*	0	-1.85	-0.50	-0.69
Oral	-1.17	-0.30	-3.71*	-0.68	-0.27	1.85	0	0.92	0.75
GIT	-2.21*	-1.33	-4.54*	-1.71	-1.22	0.50	-0.92	0	-0.14
SMG	-1.97*	-1.14	-4.38*	-1.50	-1.04	0.69	-0.75	0.14	X

Summary of specificity scale: (ECV) > (CSF) ≥ (blood, skin, saliva) ≥ (GIT, oral, SMG) ≥ olfactory

*, if p-value < 0.05

Table. Comparison table of specificity with single-population analysis with Wald Confidence Interval

Bioma-trice	CSF	Blood	ECV	Skin	Saliva	Olfactory	Oral	GIT	SMG
CSF	X	(-0.02, 0.06)	(0.01, 0.12)*	(-0.01, 0.03)	(-0.03, 0.07)	(0.05, 0.16)*	(-0.02, 0.08)	(-0.01, 0.17)	(-0.03, 0.17)
Blood	(-0.06, 0.02)	X	(-0.02, 0.06)	(-0.03, 0.05)	(-0.06, 0.06)	(0.02, 0.15)	(-0.05, 0.07)	(-0.03, 0.15)	(-0.05, 0.15)
ECV	(-0.12, -0.01)*	(-0.06, 0.02)	X	(0.02, 0.13)*	(0.01, 0.16)*	(0.10, 0.25)*	(0.02, 0.17)*	(0.04, 0.25)*	(0.02, 0.25)*
Skin	(-0.03, 0.01)	(-0.05, 0.03)	(-0.13, -0.02)*	X	(-0.12, 0.14)	(-0.03, 0.23)	(-0.11, 0.15)	(-0.17, 0.31)	(-0.21, 0.31)
Saliva	(-0.07, 0.03)	(0.06, -0.06)	(-0.16, -0.01)*	(-0.14, 0.12)	X	(-0.08, 0.25)	(-0.17, 0.19)	(-0.21, 0.33)	(-0.25, 0.35)
Olfactory	(-0.16, -0.05)*	(-0.15, -0.02)	(-0.25, -0.10)*	(-0.23, 0.03)	(-0.25, 0.08)	X	(-0.09, 0.25)	(-0.23, 0.29)	(-0.23, 0.29)

Oral	(-0.08, 0.02)	(-0.07, 0.05)	(-0.17, -0.02)*	(-0.15, 0.11)	(-0.19, 0.17)	(-0.25, 0.09)	X	(-0.22, 0.32)	(-0.25, 0.33)
GIT	(-0.17, 0.01)	(-0.15, 0.03)	(-0.25, -0.04)*	(-0.31, 0.17)	(-0.33, 0.21)	(-0.29, 0.23)	(-0.32, 0.22)	X	(-0.37, 0.35)
SMG	(-0.17, 0.03)	(-0.15, 0.05)	(-0.25, -0.02)*	(-0.31, 0.21)	(-0.35, 0.25)	(-0.29, 0.23)	(-0.33, 0.25)	(-0.35, 0.37)	X

Summary of specificity scale: (ECV) > (CSF) ≥ (olfactory, blood, skin, saliva, oral, GIT, SMG)

*, if the result is significant (not including zero)

X, not calculated

S8. League

Table. Sensitivity League				
**Biomatrix 1**	**Biomatrix 2**	**Sensitivity 1**	**Sensitivity 2**	**Difference**
C	H	0.94	0.46	0.48
D	H	0.91	0.46	0.45
B	H	0.9	0.46	0.44
A	H	0.89	0.46	0.43
C	F	0.94	0.59	0.35
H	I	0.46	0.79	0.33
D	F	0.91	0.59	0.32
B	F	0.9	0.59	0.31
R	H	0.77	0.46	0.31
A	F	0.89	0.59	0.3
C	G	0.94	0.67	0.27
D	G	0.91	0.67	0.24
B	G	0.9	0.67	0.23
A	G	0.89	0.67	0.22
G	H	0.67	0.46	0.21
F	I	0.59	0.79	0.2
R	F	0.77	0.59	0.18
C	R	0.94	0.77	0.17
C	I	0.94	0.79	0.15
D	R	0.91	0.77	0.14
B	R	0.9	0.77	0.13
F	H	0.59	0.46	0.13
A	R	0.89	0.77	0.12
D	I	0.91	0.79	0.12
G	I	0.67	0.79	0.12
B	I	0.9	0.79	0.11
A	I	0.89	0.79	0.1
R	G	0.77	0.67	0.1
F	G	0.59	0.67	0.08
A	C	0.89	0.94	0.05
B	C	0.9	0.94	0.04
C	D	0.94	0.91	0.03
A	D	0.89	0.91	0.02
R	I	0.77	0.79	0.02
B	D	0.9	0.91	0.01
A	B	0.89	0.9	0.01
Abbreviations: A) Cerebrospinal fluid; B) Blood; C) Extracellular vesicles; D) Skin; E) Saliva; F) Olfactory; G) Oral; H) Gastrointestinal tract; I) Submandibular gland.				

Table. Specificity League				
**Biomatrix 1**	**Biomatrix 2**	**Specificity 1**	**Specificity 2**	**Difference**
A	B	0.93	0.91	0.02
A	C	0.93	1	-0.07
A	D	0.93	0.92	0.01
A	E	0.93	0.91	0.02
A	F	0.93	0.82	0.11
A	G	0.93	0.9	0.03
A	H	0.93	0.85	0.08
A	I	0.93	0.86	0.07
B	C	0.91	1	-0.09
B	D	0.91	0.92	-0.01
B	E	0.91	0.91	0
B	F	0.91	0.82	0.09
B	G	0.91	0.9	0.01
B	H	0.91	0.85	0.06
B	I	0.91	0.86	0.05
C	D	1	0.92	0.08
C	E	1	0.91	0.09
C	F	1	0.82	0.18
C	G	1	0.9	0.1
C	H	1	0.85	0.15
C	I	1	0.86	0.14
D	E	0.92	0.91	0.01
D	F	0.92	0.82	0.1
D	G	0.92	0.9	0.02
D	H	0.92	0.85	0.07
D	I	0.92	0.86	0.06
E	F	0.91	0.82	0.09
E	G	0.91	0.9	0.01
E	H	0.91	0.85	0.06
E	I	0.91	0.86	0.05
F	G	0.82	0.9	-0.08
F	H	0.82	0.85	-0.03
F	I	0.82	0.86	-0.04
G	H	0.9	0.85	0.05
G	I	0.9	0.86	0.04
H	I	0.85	0.86	-0.01
Abbreviations: A) Cerebrospinal fluid; B) Blood; C) Extracellular vesicles; D) Skin; E) Saliva; F) Olfactory; G) Oral; H) Gastrointestinal tract; I) Submandibular gland.				

S9. Assay

RT-QuIC and PMCA techniques were reported for cerebrospinal fluid (CSF), skin, and gastrointestinal tract (GIT)

Youden's Index (J)

J = Sensitivity + Specificity − 1

DeLong's Test

It is a non-parametric method used to compare two correlated ROC curves by estimating the variance of the AUC difference. It is based on rank-based U-statistics. For each test, we calculate:

V (DeLong score matrix): The ranks of predictions for positive and negative cases. Given a set of true positive scores (S^+) and true negative scores (S^-). Compute the pairwise difference matrix between positive and negative cases. Convert these differences into ranks. Compute U-statistics from these ranks. The variance of the AUC estimate is given by:

$$Var(AUC) = \frac{V^+}{m} + \frac{V^-}{n}$$

Where: V^+ and V^- are variances of positive and negative score ranks; m = Number of positive cases; n = Number of negative cases.

The Z-score for comparing two AUCs is:

$$Z = \frac{AUC_1 - AUC_2}{\sqrt{Var(AUC_1) + Var(AUC_2)}}$$

where AUC1 and AUC2 are the AUC values of the two tests

Step 4: Compute p-value. The p-value is obtained from the normal distribution:

$$p = 2 \times (1 - \Phi(|Z|))$$

Φ is the cumulative distribution function (CDF) of the standard normal distribution.

Table. RT-QuIC and PMCA

Sample & Assay	PD	Controls	TP	FN	TN	FP	SN	SP	J	ROC AUC
CSF PMCA	1399	844	1260	139	782	62	0.90 (95% CI 0.88–0.91)	0.92 (95% CI 0.90–0.94)	0.827	0.914
CSF RT-QuIC	983	751	883	100	711	40	0.89 (95% CI 0.87–0.91)	0.94 (95% CI 0.93–0.96)	0.845	0.923
Skin PMCA	20	21	19	1	20	1	0.95 (95% CI 0.85–1.00)	0.95 (95% CI 0.86–1.00)	0.902	0.951
Skin RT-QuIC	184	219	164	20	199	20	0.89 (95% CI 0.84–0.93)	0.90 (95% CI 0.87–0.94)	0.800	0.900
GIT PMCA	30	20	20	10	17	3	0.66 (95% CI 0.49–0.83)	0.85 (95% CI 0.69–1.00)	0.517	0.758
GIT RT-QuIC	20	20	2	18	17	3	0.10 (95% CI -0.03–0.23)	0.85 (95% CI 0.69–1.00)	-0.050	0.475

All PMCA	1449	885	1299	150	819	66	0.89 (95% CI 0.88–0.91)	0.92 (95% CI 0.90–0.94)	0.822	0.911
All RT-QuIC	1187	990	1049	138	927	63	0.88 (95% CI 0.86–0.90)	0.93 (95% CI 0.92–0.95)	0.820	0.910
Abbreviations: CI, confidence interval; CSF, cerebrospinal fluid; FN, false negative; FP,false positive; GIT, gastrointestinal tract; J, Youden's index; PMCA, Protein misfolding cyclic amplification; ROC AUC, Receiver operating characteristic area under the curve; RT-QuIC; Real-time quaking-induced conversion; SN, sensitivity; SP, specificity; TN, true negative; TP, true positive.										

Table. RT-QuIC versus PMCA				
Comparison	SN[I]	SP[I]	J index[II]	ROC Curve & AUC
CSF PMCA versus RT-QuIC	No diference	No diference	Z-score -1.0, p-value 0.30	p-value 0.00032*
Skin PMCA versus RT-QuIC	No diference	No diference	Z-score 1.39, p-value 0.70	p-value 0.70
GIT PMCA versus RT-QuIC	Different	No diference	Z-score 3.61, p-value 0.00003*	p-value 0.0007*
PMCA[IV] versus RT-QuIC[IV]	No diference	No diference	Z-score 0.10, p-value 0.916	p-value $5.94x10^{-7}$*

Abbreviations: CI, confidence interval; CSF, cerebrospinal fluid; GIT, gastrointestinal tract; J, Youden's index; PMCA, Protein misfolding cyclic amplification; ROC AUC, Receiver operating characteristic area under the curve; RT-QuIC; Real-time quaking-induced conversion; SN, sensitivity; SP, specificity; *, significant values.

Note

[I] evaluated difference by the overlap between the confidence intervals

[II] J index results were comapred with z-test, for which the results of z-score and p-value are provided

[III] DeLong's test was performed to compare the differents AUC results, noteworthy that the size of the sample is important for the formula

[IV] PMCA from CSF, skin and GIT compared with RT-QuIC from CSF, skin and GIT

<u>AUC probabilistic results</u>

The data contains the following results (Test Name); (False Positive Rate); (True Positive Rate); (Threshold); (Test Name); (False Positive Rate); (True Positive Rate); (Threshold); etc. Note: Test 1, CSF PMCA; Test 2, CSF RT-QuIC; Test 3, Skin PMCA; Test 4, Skin RT-QuIC; Test 5, GIT PMCA; Test 6, GIT RT-QuIC; Test 7, Combined (CSF, skin, GIT) PMCA; Test 8, Combined (CSF, skin, GIT) RT-QuIC

Test 1; 0.000000; 0.000000; 1.999917; Test 1; 0.000000; 0.000715; 0.999917; Test 1; 0.000000; 0.003574; 0.998936; Test 1; 0.001185; 0.003574; 0.998877; Test 1; 0.001185; 0.004289; 0.998803; Test 1; 0.002370; 0.004289; 0.998735; Test 1; 0.002370; 0.018585; 0.995222; Test 1; 0.003555; 0.018585; 0.995213; Test 1; 0.003555; 0.037884; 0.990053; Test 1; 0.005924; 0.037884; 0.989814; Test 1; 0.005924; 0.049321; 0.985049;

Test 1; 0.007109; 0.049321; 0.984924; Test 1; 0.007109; 0.071480; 0.978090; Test 1; 0.008294; 0.071480; 0.977929; Test 1; 0.008294; 0.081487; 0.975245; Test 1; 0.009479; 0.081487; 0.975140; Test 1; 0.009479; 0.102931; 0.967043; Test 1; 0.010664; 0.102931; 0.967025; Test 1; 0.010664; 0.130808; 0.957910; Test 1; 0.011848; 0.130808; 0.957737; Test 1; 0.011848; 0.134382; 0.956710; Test 1; 0.013033; 0.134382; 0.956642;

Test 1; 0.013033; 0.146533; 0.953571; Test 1; 0.014218; 0.146533; 0.953499; Test 1; 0.014218; 0.160114; 0.949304; Test 1; 0.015403; 0.160114; 0.949085; Test 1; 0.015403; 0.170836; 0.945658; Test 1; 0.016588; 0.170836; 0.945303; Test 1; 0.016588; 0.176555; 0.943940; Test 1; 0.017773; 0.176555; 0.943720; Test 1; 0.017773; 0.192280; 0.939728; Test 1; 0.018957; 0.192280; 0.939340; Test 1; 0.018957; 0.204432; 0.936197;

Test 1; 0.020142; 0.204432; 0.935994; Test 1; 0.020142; 0.233024; 0.925506; Test 1; 0.021327; 0.233024; 0.925491; Test 1; 0.021327; 0.258041; 0.916056; Test 1; 0.022512; 0.258041; 0.915658; Test 1; 0.022512; 0.314510; 0.899201; Test 1; 0.023697; 0.314510; 0.899134; Test 1; 0.023697; 0.330236; 0.893911; Test 1; 0.024882; 0.330236; 0.893785; Test 1; 0.024882; 0.335954; 0.891813; Test 1; 0.026066; 0.335954; 0.891295;

Test 1; 0.026066; 0.355969; 0.886621; Test 1; 0.027251; 0.355969; 0.886424; Test 1; 0.027251; 0.363117; 0.885126; Test 1; 0.028436; 0.363117; 0.884469; Test 1; 0.028436; 0.375983; 0.881132; Test 1; 0.029621; 0.375983; 0.880783; Test 1; 0.029621; 0.377412; 0.880558; Test 1; 0.030806; 0.377412; 0.880442; Test 1; 0.030806; 0.385275; 0.878472; Test 1; 0.031991; 0.385275; 0.878242; Test 1; 0.031991; 0.386705; 0.877880;

Test 1; 0.033175; 0.386705; 0.877878; Test 1; 0.033175; 0.395997; 0.874692; Test 1; 0.034360; 0.395997; 0.874556; Test 1; 0.034360; 0.418156; 0.868900; Test 1; 0.035545; 0.418156; 0.868465; Test 1; 0.035545; 0.420300; 0.867625; Test 1; 0.036730; 0.420300; 0.867220; Test 1; 0.036730; 0.431737; 0.863913; Test 1; 0.037915; 0.431737; 0.863780; Test 1; 0.037915; 0.438885; 0.861112; Test 1; 0.039100; 0.438885; 0.860830;

Test 1; 0.039100; 0.478914; 0.845861; Test 1; 0.040284; 0.478914; 0.845598; Test 1; 0.040284; 0.488921; 0.840863; Test 1; 0.041469; 0.488921; 0.840720; Test 1; 0.041469; 0.493924; 0.838267; Test 1; 0.042654; 0.493924; 0.837650; Test 1; 0.042654; 0.528949; 0.827225; Test 1; 0.043839; 0.528949; 0.827164; Test 1; 0.043839; 0.567548; 0.816536; Test 1; 0.045024; 0.567548; 0.815895; Test 1; 0.045024; 0.570407; 0.814758;

Test 1; 0.046209; 0.570407; 0.814569; Test 1; 0.046209; 0.598284; 0.801453; Test 1; 0.047393; 0.598284; 0.801108; Test 1; 0.047393; 0.604718; 0.798821; Test 1; 0.048578; 0.604718; 0.798480; Test 1; 0.048578; 0.609721; 0.796744; Test 1; 0.049763; 0.609721; 0.796718; Test 1; 0.049763; 0.646176; 0.783396; Test 1; 0.050948; 0.646176; 0.783050; Test 1; 0.050948; 0.674053; 0.773533; Test 1; 0.052133; 0.674053; 0.773376;

Test 1; 0.052133; 0.686919; 0.769032; Test 1; 0.053318; 0.686919; 0.768969; Test 1; 0.053318; 0.701930; 0.764888; Test 1; 0.054502; 0.701930; 0.764807; Test 1; 0.054502; 0.702645; 0.764644; Test 1; 0.055687; 0.702645; 0.764213; Test 1; 0.055687; 0.703360; 0.764208; Test 1; 0.056872; 0.703360; 0.763963; Test 1; 0.056872; 0.724089; 0.757289; Test 1; 0.058057; 0.724089; 0.757112; Test 1; 0.058057; 0.736955; 0.754490;

Test 1; 0.059242; 0.736955; 0.754193; Test 1; 0.059242; 0.741959; 0.752143; Test 1; 0.060427; 0.741959; 0.752138; Test 1; 0.060427; 0.793424; 0.736841; Test 1; 0.062796; 0.793424; 0.736261; Test 1; 0.062796; 0.794139; 0.736218; Test 1; 0.063981; 0.794139; 0.736019; Test 1; 0.063981; 0.812009; 0.732033; Test 1; 0.065166; 0.812009; 0.731974; Test 1; 0.065166; 0.826305; 0.725464; Test 1; 0.066351; 0.826305; 0.724877;

Test 1; 0.066351; 0.832023; 0.723035; Test 1; 0.067536; 0.832023; 0.723023; Test 1; 0.067536; 0.837026; 0.720178; Test 1; 0.068720; 0.837026; 0.719915; Test 1; 0.068720; 0.855611; 0.714850; Test 1; 0.069905; 0.855611; 0.714170; Test 1; 0.069905; 0.864904; 0.711605; Test 1; 0.071090; 0.864904; 0.711207; Test 1; 0.071090; 0.868477; 0.709945; Test 1; 0.072275; 0.868477; 0.709794; Test 1; 0.072275; 0.889207; 0.704214;

Test 1; 0.073460; 0.889207; 0.704036; Test 1; 0.073460; 0.901358; 0.298786; Test 1; 0.086493; 0.901358; 0.294898; Test 1; 0.086493; 0.902073; 0.294599; Test 1; 0.095972; 0.902073; 0.291596; Test 1; 0.095972; 0.903503; 0.290789; Test 1; 0.098341; 0.903503; 0.289604; Test 1; 0.098341; 0.904217; 0.289240; Test 1; 0.111374; 0.904217; 0.286793; Test 1; 0.111374; 0.904932; 0.286604; Test 1; 0.113744; 0.904932; 0.285481;

Test 1; 0.113744; 0.905647; 0.285053; Test 1; 0.117299; 0.905647; 0.283602; Test 1; 0.117299; 0.906362; 0.283576; Test 1; 0.125592; 0.906362; 0.281231; Test 1; 0.125592; 0.907076; 0.281225; Test 1; 0.148104; 0.907076; 0.273206; Test 1; 0.148104; 0.907791; 0.273152; Test 1; 0.161137; 0.907791; 0.269184; Test 1; 0.161137; 0.908506; 0.268942; Test 1; 0.169431; 0.908506; 0.266571; Test 1; 0.169431; 0.909221; 0.264803;

Test 1; 0.171801; 0.909221; 0.264641; Test 1; 0.171801; 0.909936; 0.263631; Test 1; 0.175355; 0.909936; 0.261676; Test 1; 0.175355; 0.910650; 0.261634; Test 1; 0.176540; 0.910650; 0.261536; Test 1; 0.176540; 0.911365; 0.261528; Test 1; 0.187204; 0.911365; 0.259204; Test 1; 0.187204; 0.912080; 0.258912; Test 1; 0.193128; 0.912080; 0.256792; Test 1; 0.193128; 0.912795; 0.256674; Test 1; 0.195498; 0.912795; 0.256486;

Test 1; 0.195498; 0.913510; 0.255943; Test 1; 0.197867; 0.913510; 0.255850; Test 1; 0.197867; 0.914224; 0.255451; Test 1; 0.203791; 0.914224; 0.253730; Test 1; 0.203791; 0.914939; 0.253728; Test 1; 0.208531; 0.914939; 0.253473; Test 1; 0.208531; 0.915654; 0.253396; Test 1; 0.216825; 0.915654; 0.250661; Test 1; 0.216825; 0.916369; 0.250416; Test 1; 0.227488; 0.916369; 0.246469; Test 1; 0.227488; 0.917798; 0.244971;

Test 1; 0.232227; 0.917798; 0.243313; Test 1; 0.232227; 0.918513; 0.242922; Test 1; 0.241706; 0.918513; 0.240670; Test 1; 0.241706; 0.919228; 0.240313; Test 1; 0.266588; 0.919228; 0.233261; Test 1; 0.266588; 0.920658; 0.232755; Test 1; 0.273697; 0.920658; 0.231562; Test 1; 0.273697; 0.922087; 0.231024; Test 1; 0.276066; 0.922087; 0.230762; Test 1; 0.276066; 0.923517; 0.230139; Test 1; 0.277251; 0.923517; 0.230039;

Test 1; 0.277251; 0.924232; 0.229991; Test 1; 0.281991; 0.924232; 0.229439; Test 1; 0.281991; 0.924946; 0.229284; Test 1; 0.286730; 0.924946; 0.228762; Test 1; 0.286730; 0.925661; 0.228532; Test 1; 0.292654; 0.925661; 0.227510; Test 1; 0.292654; 0.926376; 0.227304; Test 1; 0.296209; 0.926376; 0.226409; Test 1; 0.296209; 0.927091; 0.226396; Test 1; 0.303318; 0.927091; 0.224525; Test 1; 0.303318; 0.927806; 0.223519;

Test 1; 0.319905; 0.927806; 0.218651; Test 1; 0.319905; 0.928520; 0.218474; Test 1; 0.335308; 0.928520; 0.213855; Test 1; 0.335308; 0.929235; 0.213792; Test 1; 0.355450; 0.929235; 0.208233; Test 1; 0.355450; 0.929950; 0.208222; Test 1; 0.360190; 0.929950; 0.207465; Test 1; 0.360190; 0.930665; 0.206837; Test 1; 0.363744; 0.930665; 0.206591; Test 1; 0.363744; 0.931380; 0.205719; Test 1; 0.368483; 0.931380; 0.203114;

Test 1; 0.368483; 0.932094; 0.202834; Test 1; 0.376777; 0.932094; 0.201262; Test 1; 0.376777; 0.932809; 0.201262; Test 1; 0.380332; 0.932809; 0.199717; Test 1; 0.380332; 0.933524; 0.198561; Test 1; 0.381517; 0.933524; 0.198016; Test 1; 0.381517; 0.934239; 0.197671; Test 1; 0.386256; 0.934239; 0.197152; Test 1; 0.386256; 0.934954; 0.196574; Test 1; 0.400474; 0.934954; 0.193732; Test 1; 0.400474; 0.935668; 0.193681;

Test 1; 0.401659; 0.935668; 0.193540; Test 1; 0.401659; 0.936383; 0.193394; Test 1; 0.407583; 0.936383; 0.192515; Test 1; 0.407583; 0.937098; 0.192291; Test 1; 0.420616; 0.937098; 0.189681; Test 1; 0.420616; 0.937813; 0.188545; Test 1; 0.422986; 0.937813; 0.187593; Test 1; 0.422986; 0.939242; 0.186704; Test 1; 0.424171; 0.939242; 0.186526; Test 1; 0.424171; 0.939957; 0.186424; Test 1; 0.436019; 0.939957; 0.181056;

Test 1; 0.436019; 0.940672; 0.180097; Test 1; 0.449052; 0.940672; 0.176679; Test 1; 0.449052; 0.941387; 0.176629; Test 1; 0.454976; 0.941387; 0.175483; Test 1; 0.454976; 0.942816; 0.174216; Test 1; 0.457346; 0.942816; 0.173825; Test 1; 0.457346; 0.943531; 0.173259; Test 1; 0.459716; 0.943531; 0.173183; Test 1; 0.459716; 0.945675; 0.172048; Test 1; 0.462085; 0.945675; 0.171750; Test 1; 0.462085; 0.946390; 0.171175;

Test 1; 0.465640; 0.946390; 0.169851; Test 1; 0.465640; 0.947105; 0.169564; Test 1; 0.478673; 0.947105; 0.167459; Test 1; 0.478673; 0.947820; 0.167417; Test 1; 0.489336; 0.947820; 0.164509; Test 1; 0.489336; 0.948535; 0.163735; Test 1; 0.492891; 0.948535; 0.162826; Test 1; 0.492891; 0.949964; 0.161689; Test 1; 0.503555; 0.949964; 0.158869; Test 1; 0.503555; 0.950679; 0.158819; Test 1; 0.513033; 0.950679; 0.157064;

Test 1; 0.513033; 0.951394; 0.156999; Test 1; 0.549763; 0.951394; 0.148134; Test 1; 0.549763; 0.952109; 0.148093; Test 1; 0.566351; 0.952109; 0.144416; Test 1; 0.566351; 0.952823; 0.144239; Test 1; 0.569905; 0.952823; 0.144031; Test 1; 0.569905; 0.953538; 0.143031; Test 1; 0.609005; 0.953538; 0.134243; Test 1; 0.609005; 0.954253; 0.133866; Test 1; 0.610190; 0.954253; 0.133436; Test 1; 0.610190; 0.955683; 0.132732;

Test 1; 0.613744; 0.955683; 0.132321; Test 1; 0.613744; 0.957112; 0.131967; Test 1; 0.614929; 0.957112; 0.131302; Test 1; 0.614929; 0.957827; 0.131136; Test 1; 0.623223; 0.957827; 0.126877; Test 1; 0.623223; 0.958542; 0.126811; Test 1; 0.624408; 0.958542; 0.126806; Test 1; 0.624408; 0.959257; 0.126286; Test 1; 0.629147; 0.959257; 0.125394; Test 1; 0.629147; 0.959971; 0.125210; Test 1; 0.630332; 0.959971; 0.124983;

Test 1; 0.630332; 0.960686; 0.124446; Test 1; 0.642180; 0.960686; 0.120625; Test 1; 0.642180; 0.961401; 0.120500; Test 1; 0.650474; 0.961401; 0.119448; Test 1; 0.650474; 0.962116; 0.118964; Test 1; 0.656398; 0.962116; 0.118209; Test 1; 0.656398; 0.962831; 0.118015; Test 1; 0.659953; 0.962831; 0.117819; Test 1; 0.659953; 0.963545; 0.117685; Test 1; 0.668246; 0.963545; 0.113628; Test 1; 0.668246; 0.964260; 0.113523;

Test 1; 0.674171; 0.964260; 0.111425; Test 1; 0.674171; 0.964975; 0.111390; Test 1; 0.676540; 0.964975; 0.109383; Test 1; 0.676540; 0.966405; 0.108968; Test 1; 0.681280; 0.966405; 0.107073; Test 1; 0.681280; 0.967119; 0.106561; Test 1; 0.682464; 0.967119; 0.105981; Test 1; 0.682464; 0.967834; 0.104006; Test 1; 0.695498; 0.967834; 0.100465; Test 1; 0.695498; 0.969979; 0.099933; Test 1; 0.696682; 0.969979; 0.099541;

Test 1; 0.696682; 0.970693; 0.098895; Test 1; 0.697867; 0.970693; 0.098533; Test 1; 0.697867; 0.971408; 0.098456; Test 1; 0.713270; 0.971408; 0.093748; Test 1; 0.713270; 0.972123; 0.091105; Test 1; 0.715640; 0.972123; 0.090633; Test 1; 0.715640; 0.972838; 0.090520; Test 1; 0.718009; 0.972838; 0.090244; Test 1; 0.718009; 0.973553; 0.090041; Test 1; 0.720379; 0.973553; 0.089706; Test 1; 0.720379; 0.974267; 0.089606;

Test 1; 0.750000; 0.974267; 0.077760; Test 1; 0.750000; 0.974982; 0.077676; Test 1; 0.773697; 0.974982; 0.069949; Test 1; 0.773697; 0.975697; 0.069921; Test 1; 0.778436; 0.975697; 0.068507; Test 1; 0.778436; 0.976412; 0.068321; Test 1; 0.781991; 0.976412; 0.067366; Test 1; 0.781991; 0.977127; 0.067093; Test 1; 0.783175; 0.977127; 0.066549; Test 1; 0.783175; 0.977841; 0.066228; Test 1; 0.785545; 0.977841; 0.065449;

Test 1; 0.785545; 0.978556; 0.065114; Test 1; 0.787915; 0.978556; 0.064976; Test 1; 0.787915; 0.979271; 0.064830; Test 1; 0.796209; 0.979271; 0.062981; Test 1; 0.796209; 0.979986; 0.062009; Test 1; 0.804502; 0.979986; 0.060091; Test 1; 0.804502; 0.980701; 0.060008; Test 1; 0.805687; 0.980701; 0.059757; Test 1; 0.805687; 0.981415; 0.058899; Test 1; 0.812796; 0.981415; 0.056514; Test 1; 0.812796; 0.982130; 0.056250;

Test 1; 0.817536; 0.982130; 0.055029; Test 1; 0.817536; 0.982845; 0.054926; Test 1; 0.837678; 0.982845; 0.050645; Test 1; 0.837678; 0.983560; 0.049978; Test 1; 0.841232; 0.983560; 0.049143; Test 1; 0.841232; 0.984274; 0.048807; Test 1; 0.844787; 0.984274; 0.047834; Test 1; 0.844787; 0.985704; 0.047610; Test 1; 0.856635; 0.985704; 0.043537; Test 1; 0.856635; 0.986419; 0.043312; Test 1; 0.864929; 0.986419; 0.041681;

Test 1; 0.864929; 0.987134; 0.041405; Test 1; 0.885071; 0.987134; 0.036090; Test 1; 0.885071; 0.987848; 0.035485; Test 1; 0.904028; 0.987848; 0.029653; Test 1; 0.904028; 0.989278; 0.029533; Test 1; 0.906398; 0.989278; 0.029002; Test 1; 0.906398; 0.989993; 0.028992; Test 1; 0.913507; 0.989993; 0.027008; Test 1; 0.913507; 0.990708; 0.026813; Test 1; 0.921801; 0.990708; 0.024287; Test 1; 0.921801; 0.991422; 0.023884;

Test 1; 0.927725; 0.991422; 0.022343; Test 1; 0.927725; 0.992137; 0.022203; Test 1; 0.933649; 0.992137; 0.021051; Test 1; 0.933649; 0.992852; 0.020781; Test 1; 0.943128; 0.992852; 0.016957; Test 1; 0.943128; 0.993567; 0.016305; Test 1; 0.952607; 0.993567; 0.013204; Test 1; 0.952607; 0.994996; 0.012753; Test 1; 0.957346; 0.994996; 0.010005; Test 1; 0.957346; 0.995711; 0.009691; Test 1; 0.958531; 0.995711; 0.009683;

Test 1; 0.958531; 0.996426; 0.009388; Test 1; 0.964455; 0.996426; 0.008132; Test 1; 0.964455; 0.997141; 0.008027; Test 1; 0.970379; 0.997141; 0.006258; Test 1; 0.970379; 0.997856; 0.005831; Test 1; 0.985782; 0.997856; 0.002202; Test 1; 0.985782; 0.999285; 0.001973; Test 1; 0.986967; 0.999285; 0.001797; Test 1; 0.986967; 1.000000; 0.001681; Test 1; 1.000000; 1.000000; 0.000093; Test 2; 0.000000; 0.000000; 1.999968;

Test 2; 0.000000; 0.001017; 0.999968; Test 2; 0.000000; 0.002035; 0.999869; Test 2; 0.001332; 0.002035; 0.999418; Test 2; 0.001332; 0.035605; 0.988347; Test 2; 0.002663; 0.035605; 0.988334; Test 2; 0.002663; 0.072228; 0.977864; Test 2; 0.003995; 0.072228; 0.977731; Test 2; 0.003995; 0.077314; 0.976774; Test 2; 0.005326; 0.077314; 0.976462; Test 2; 0.005326; 0.145473; 0.954386; Test 2; 0.006658; 0.145473; 0.953739;

Test 2; 0.006658; 0.219736; 0.924727; Test 2; 0.007989; 0.219736; 0.924310; Test 2; 0.007989; 0.224822; 0.923627; Test 2; 0.009321; 0.224822; 0.923424; Test 2; 0.009321; 0.231943; 0.921466; Test 2; 0.010652; 0.231943; 0.921429; Test 2; 0.010652; 0.259410; 0.910864; Test 2; 0.011984; 0.259410; 0.910860; Test 2; 0.011984; 0.279756; 0.904675; Test 2; 0.013316; 0.279756; 0.903795; Test 2; 0.013316; 0.297050; 0.899926;

Test 2; 0.014647; 0.297050; 0.899897; Test 2; 0.014647; 0.325534; 0.891608; Test 2; 0.015979; 0.325534; 0.890210; Test 2; 0.015979; 0.328586; 0.889025; Test 2; 0.017310; 0.328586; 0.888612; Test 2; 0.017310; 0.341811; 0.884768; Test 2; 0.018642; 0.341811; 0.884498; Test 2; 0.018642; 0.449644; 0.851534; Test 2; 0.019973; 0.449644; 0.851428; Test 2; 0.019973; 0.455748; 0.848542; Test 2; 0.021305; 0.455748; 0.847525;

Test 2; 0.021305; 0.468973; 0.842413; Test 2; 0.022636; 0.468973; 0.841816; Test 2; 0.022636; 0.469990; 0.841731; Test 2; 0.023968; 0.469990; 0.841647; Test 2; 0.023968; 0.474059; 0.840643; Test 2; 0.025300; 0.474059; 0.840564; Test 2; 0.025300; 0.505595; 0.834725; Test 2; 0.026631; 0.505595; 0.834458; Test 2; 0.026631; 0.536114; 0.824482; Test 2; 0.027963; 0.536114; 0.824472; Test 2; 0.027963; 0.547304; 0.820519;

Test 2; 0.029294; 0.547304; 0.820179; Test 2; 0.029294; 0.577823; 0.808488; Test 2; 0.030626; 0.577823; 0.807879; Test 2; 0.030626; 0.582909; 0.806342; Test 2; 0.031957; 0.582909; 0.805569; Test 2; 0.031957; 0.606307; 0.796902; Test 2; 0.033289; 0.606307; 0.796576; Test 2; 0.033289; 0.622584; 0.791992; Test 2; 0.034621; 0.622584; 0.791291; Test 2; 0.034621; 0.640895; 0.786925; Test 2; 0.035952; 0.640895; 0.786323;

Test 2; 0.035952; 0.649034; 0.784816; Test 2; 0.037284; 0.649034; 0.784146; Test 2; 0.037284; 0.674466; 0.779329; Test 2; 0.038615; 0.674466; 0.779254; Test 2; 0.038615; 0.684639; 0.776180; Test 2; 0.039947; 0.684639; 0.776054; Test 2; 0.039947; 0.721261; 0.766580; Test 2; 0.041278; 0.721261; 0.766095; Test 2; 0.041278; 0.758901; 0.751431; Test 2; 0.042610; 0.758901; 0.751104; Test 2; 0.042610; 0.767040; 0.747683;

Test 2; 0.043941; 0.767040; 0.746674; Test 2; 0.043941; 0.773143; 0.745022; Test 2; 0.045273; 0.773143; 0.744956; Test 2; 0.045273; 0.781282; 0.741024; Test 2; 0.046605; 0.781282; 0.740941; Test 2; 0.046605; 0.793489; 0.735178; Test 2; 0.047936; 0.793489; 0.734853; Test 2; 0.047936; 0.797558; 0.733103; Test 2; 0.049268; 0.797558; 0.732586; Test 2; 0.049268; 0.798576; 0.732286; Test 2; 0.050599; 0.798576; 0.732183;

Test 2; 0.050599; 0.837233; 0.722181; Test 2; 0.051931; 0.837233; 0.721946; Test 2; 0.051931; 0.895219; 0.703732; Test 2; 0.053262; 0.895219; 0.702652; Test 2; 0.053262; 0.899288; 0.299630; Test 2; 0.066578; 0.899288; 0.296799; Test 2; 0.066578; 0.900305; 0.296648; Test 2; 0.078562; 0.900305; 0.294499; Test 2; 0.078562; 0.901322; 0.294224; Test 2; 0.085220; 0.901322; 0.291751; Test 2; 0.085220; 0.902340; 0.291516;

Test 2; 0.093209; 0.902340; 0.288554; Test 2; 0.093209; 0.903357; 0.288216; Test 2; 0.115846; 0.903357; 0.281913; Test 2; 0.115846; 0.904374; 0.281094; Test 2; 0.119840; 0.904374; 0.280238; Test 2; 0.119840; 0.906409; 0.278648; Test 2; 0.125166; 0.906409; 0.277527; Test 2; 0.125166; 0.907426; 0.277210; Test 2; 0.142477; 0.907426; 0.270404; Test 2; 0.142477; 0.908444; 0.270400; Test 2; 0.151798; 0.908444; 0.267904;

Test 2; 0.151798; 0.909461; 0.267875; Test 2; 0.154461; 0.909461; 0.267634; Test 2; 0.154461; 0.910478; 0.267607; Test 2; 0.155792; 0.910478; 0.267450; Test 2; 0.155792; 0.911495; 0.266681; Test 2; 0.165113; 0.911495; 0.263676; Test 2; 0.165113; 0.912513; 0.262978; Test 2; 0.174434; 0.912513; 0.260579; Test 2; 0.174434; 0.913530; 0.260040; Test 2; 0.214381; 0.913530; 0.249387; Test 2; 0.214381; 0.914547; 0.248597;

Test 2; 0.227696; 0.914547; 0.246351; Test 2; 0.227696; 0.915565; 0.245978; Test 2; 0.241012; 0.915565; 0.242892; Test 2; 0.241012; 0.916582; 0.242867; Test 2; 0.242344; 0.916582; 0.242798; Test 2; 0.242344; 0.917599; 0.242505; Test 2; 0.245007; 0.917599; 0.242459; Test 2; 0.245007; 0.918616; 0.242295; Test 2; 0.250333; 0.918616; 0.240292; Test 2; 0.250333; 0.919634; 0.239860; Test 2; 0.252996; 0.919634; 0.239486;

Test 2; 0.252996; 0.920651; 0.239376; Test 2; 0.256991; 0.920651; 0.237988; Test 2; 0.256991; 0.921668; 0.237848; Test 2; 0.259654; 0.921668; 0.234761; Test 2; 0.259654; 0.922686; 0.234247; Test 2; 0.263648; 0.922686; 0.233521; Test 2; 0.263648; 0.924720; 0.232339; Test 2; 0.276964; 0.924720; 0.228810; Test 2; 0.276964; 0.925738; 0.228583; Test 2; 0.284953; 0.925738; 0.225420; Test 2; 0.284953; 0.926755; 0.224984;

Test 2; 0.300932; 0.926755; 0.221177; Test 2; 0.300932; 0.928789; 0.220559; Test 2; 0.304927; 0.928789; 0.220280; Test 2; 0.304927; 0.929807; 0.220061; Test 2; 0.315579; 0.929807; 0.216566; Test 2; 0.315579; 0.930824; 0.215786; Test 2; 0.330226; 0.930824; 0.210700; Test 2; 0.330226; 0.931841; 0.210303; Test 2; 0.340879; 0.931841; 0.208804; Test 2; 0.340879; 0.932859; 0.207857; Test 2; 0.372836; 0.932859; 0.199782;

Test 2; 0.372836; 0.933876; 0.198284; Test 2; 0.380826; 0.933876; 0.196665; Test 2; 0.380826; 0.934893; 0.196655; Test 2; 0.387483; 0.934893; 0.195294; Test 2; 0.387483; 0.935910; 0.194109; Test 2; 0.394141; 0.935910; 0.193154; Test 2; 0.394141; 0.936928; 0.192457; Test 2; 0.399467; 0.936928; 0.190652; Test 2; 0.399467; 0.937945; 0.189984; Test 2; 0.400799; 0.937945; 0.189880; Test 2; 0.400799; 0.938962; 0.189609;

Test 2; 0.411451; 0.938962; 0.187853; Test 2; 0.411451; 0.939980; 0.187731; Test 2; 0.419441; 0.939980; 0.186350; Test 2; 0.419441; 0.940997; 0.185861; Test 2; 0.426099; 0.940997; 0.184498; Test 2; 0.426099; 0.942014; 0.183700; Test 2; 0.431425; 0.942014; 0.182458; Test 2; 0.431425; 0.943032; 0.181433; Test 2; 0.432756; 0.943032; 0.181198; Test 2; 0.432756; 0.944049; 0.180637; Test 2; 0.435419; 0.944049; 0.180247;

Test 2; 0.435419; 0.945066; 0.180066; Test 2; 0.442077; 0.945066; 0.178389; Test 2; 0.442077; 0.946083; 0.176992; Test 2; 0.455393; 0.946083; 0.174329; Test 2; 0.455393; 0.947101; 0.174268; Test 2; 0.507324; 0.947101; 0.161100; Test 2; 0.507324; 0.948118; 0.161077; Test 2; 0.519308; 0.948118; 0.155414; Test 2; 0.519308; 0.949135; 0.155251; Test 2; 0.523302; 0.949135; 0.154284; Test 2; 0.523302; 0.950153; 0.154185;

Test 2; 0.553928; 0.950153; 0.148946; Test 2; 0.553928; 0.953204; 0.148516; Test 2; 0.561917; 0.953204; 0.147503; Test 2; 0.561917; 0.954222; 0.147399; Test 2; 0.591212; 0.954222; 0.139016; Test 2; 0.591212; 0.955239; 0.138658; Test 2; 0.595206; 0.955239; 0.137457; Test 2; 0.595206; 0.956256; 0.137233; Test 2; 0.597870; 0.956256; 0.136928; Test 2; 0.597870; 0.957274; 0.136460; Test 2; 0.625832; 0.957274; 0.126543;

Test 2; 0.625832; 0.958291; 0.125630; Test 2; 0.647137; 0.958291; 0.118123; Test 2; 0.647137; 0.959308; 0.118053; Test 2; 0.655126; 0.959308; 0.116314; Test 2; 0.655126; 0.961343; 0.116098; Test 2; 0.668442; 0.961343; 0.112287; Test 2; 0.668442; 0.962360; 0.111311; Test 2; 0.687084; 0.962360; 0.105451; Test 2; 0.687084; 0.963377; 0.105011; Test 2; 0.693742; 0.963377; 0.103312; Test 2; 0.693742; 0.965412; 0.102838;

Test 2; 0.695073; 0.965412; 0.102443; Test 2; 0.695073; 0.967447; 0.100286; Test 2; 0.707057; 0.967447; 0.098203; Test 2; 0.707057; 0.968464; 0.096623; Test 2; 0.708389; 0.968464; 0.095907; Test 2; 0.708389; 0.969481; 0.095854; Test 2; 0.716378; 0.969481; 0.091805; Test 2; 0.716378; 0.970498; 0.091462; Test 2; 0.728362; 0.970498; 0.086942; Test 2; 0.728362; 0.971516; 0.086197; Test 2; 0.740346; 0.971516; 0.082143;

Test 2; 0.740346; 0.972533; 0.081996; Test 2; 0.756325; 0.972533; 0.077446; Test 2; 0.756325; 0.973550; 0.077369; Test 2; 0.756988; 0.973550; 0.075302; Test 2; 0.758988; 0.974568; 0.075094; Test 2; 0.761651; 0.974568; 0.074725; Test 2; 0.761651; 0.975585; 0.073845; Test 2; 0.765646; 0.975585; 0.071671; Test 2; 0.765646; 0.976602; 0.071463; Test 2; 0.781625; 0.976602; 0.067303; Test 2; 0.781625; 0.977620; 0.067024;

Test 2; 0.782956; 0.977620; 0.067019; Test 2; 0.782956; 0.978637; 0.066460; Test 2; 0.797603; 0.978637; 0.063155; Test 2; 0.797603; 0.979654; 0.062847; Test 2; 0.809587; 0.979654; 0.059031; Test 2; 0.809587; 0.981689; 0.057949; Test 2; 0.812250; 0.981689; 0.057655; Test 2; 0.812250; 0.982706; 0.057489; Test 2; 0.822903; 0.982706; 0.053584; Test 2; 0.822903; 0.983723; 0.053121; Test 2; 0.828229; 0.983723; 0.052551;

Test 2; 0.828229; 0.984741; 0.052208; Test 2; 0.861518; 0.984741; 0.043945; Test 2; 0.861518; 0.985758; 0.043878; Test 2; 0.862850; 0.985758; 0.043754; Test 2; 0.862850; 0.986775; 0.043693; Test 2; 0.870839; 0.986775; 0.040981; Test 2; 0.870839; 0.987792; 0.040843; Test 2; 0.877497; 0.987792; 0.038599; Test 2; 0.877497; 0.988810; 0.037962; Test 2; 0.881491; 0.988810; 0.036249; Test 2; 0.881491; 0.990844; 0.035500;

Test 2; 0.885486; 0.990844; 0.034908; Test 2; 0.885486; 0.991862; 0.034784; Test 2; 0.888149; 0.991862; 0.033383; Test 2; 0.888149; 0.992879; 0.032437; Test 2; 0.896138; 0.992879; 0.030468; Test 2; 0.896138; 0.993896; 0.029462; Test 2; 0.918775; 0.993896; 0.024706; Test 2; 0.918775; 0.994914; 0.024157; Test 2; 0.936085; 0.994914; 0.018519; Test 2; 0.936085; 0.995931; 0.017971; Test 2; 0.948069; 0.995931; 0.013301;

Test 2; 0.948069; 0.996948; 0.013152; Test 2; 0.957390; 0.996948; 0.011759; Test 2; 0.957390; 0.997965; 0.010847; Test 2; 0.980027; 0.997965; 0.007082; Test 2; 0.980027; 0.998983; 0.006821; Test 2; 0.982690; 0.998983; 0.005658; Test 2; 0.982690; 1.000000; 0.004615; Test 2; 1.000000; 1.000000; 0.000208; Test 3; 0.000000; 0.000000; 1.999956; Test 3; 0.000000; 0.050000; 0.999956; Test 3; 0.000000; 0.100000; 0.990948;

Test 3; 0.047619; 0.100000; 0.979349; Test 3; 0.047619; 0.950000; 0.713646; Test 3; 0.571429; 0.950000; 0.130024; Test 3; 0.571429; 1.000000; 0.125860; Test 3; 1.000000; 1.000000; 0.004180; Test 4; 0.000000; 0.000000; 1.999935; Test 4; 0.000000; 0.005435; 0.999935; Test 4; 0.000000; 0.010870; 0.999436; Test 4; 0.004566; 0.010870; 0.997893; Test 4; 0.004566; 0.038043; 0.992219; Test 4; 0.009132; 0.038043; 0.991704;

Test 4; 0.009132; 0.070652; 0.982650; Test 4; 0.013699; 0.070652; 0.982532; Test 4; 0.013699; 0.130435; 0.966631; Test 4; 0.018265; 0.130435; 0.965263; Test 4; 0.018265; 0.163043; 0.952443; Test 4; 0.022831; 0.163043; 0.951708; Test 4; 0.022831; 0.211957; 0.936703; Test 4; 0.027397; 0.211957; 0.936373; Test 4; 0.027397; 0.222826; 0.931510; Test 4; 0.031963; 0.222826; 0.931347; Test 4; 0.031963; 0.342391; 0.893639;

Test 4; 0.036530; 0.342391; 0.893399; Test 4; 0.036530; 0.434783; 0.855350; Test 4; 0.041096; 0.434783; 0.854973; Test 4; 0.041096; 0.467391; 0.838823; Test 4; 0.045662; 0.467391; 0.838160; Test 4; 0.045662; 0.483696; 0.836993; Test 4; 0.050228; 0.483696; 0.836594; Test 4; 0.050228; 0.489130; 0.832842; Test 4; 0.054795; 0.489130; 0.832195; Test 4; 0.054795; 0.586957; 0.800707; Test 4; 0.059361; 0.586957; 0.800387;

Test 4; 0.059361; 0.750000; 0.747229; Test 4; 0.068493; 0.750000; 0.742813; Test 4; 0.068493; 0.766304; 0.738634; Test 4; 0.073059; 0.766304; 0.737680; Test 4; 0.073059; 0.788043; 0.735310; Test 4; 0.077626; 0.788043; 0.734457; Test 4; 0.077626; 0.804348; 0.730151; Test 4; 0.082192; 0.804348; 0.729749; Test 4; 0.082192; 0.826087; 0.726282; Test 4; 0.091324; 0.826087; 0.722906; Test 4; 0.091324; 0.891304; 0.700225;

Test 4; 0.159817; 0.891304; 0.275891; Test 4; 0.159817; 0.896739; 0.273518; Test 4; 0.168950; 0.896739; 0.270637; Test 4; 0.168950; 0.902174; 0.269415; Test 4; 0.296804; 0.902174; 0.224142; Test 4; 0.296804; 0.907609; 0.223610; Test 4; 0.324201; 0.907609; 0.218285; Test 4; 0.324201; 0.913043; 0.217029; Test 4; 0.365297; 0.913043; 0.205603; Test 4; 0.365297; 0.918478; 0.204805; Test 4; 0.420091; 0.918478; 0.194224;

Test 4; 0.420091; 0.923913; 0.193792; Test 4; 0.438356; 0.923913; 0.190627; Test 4; 0.438356; 0.929348; 0.190310; Test 4; 0.497717; 0.929348; 0.158525; Test 4; 0.497717; 0.934783; 0.157676; Test 4; 0.543379; 0.934783; 0.149914; Test 4; 0.543379; 0.940217; 0.149355; Test 4; 0.561644; 0.940217; 0.145001; Test 4; 0.561644; 0.945652; 0.142660; Test 4; 0.584475; 0.945652; 0.137556; Test 4; 0.584475; 0.951087; 0.135728;

Test 4; 0.598174; 0.951087; 0.132825; Test 4; 0.598174; 0.956522; 0.127952; Test 4; 0.625571; 0.956522; 0.119566; Test 4; 0.625571; 0.961957; 0.118717; Test 4; 0.643836; 0.961957; 0.108656; Test 4; 0.643836; 0.967391; 0.108491; Test 4; 0.703196; 0.967391; 0.096752; Test 4; 0.703196; 0.972826; 0.096513; Test 4; 0.716895; 0.972826; 0.094712; Test 4; 0.716895; 0.978261; 0.092068; Test 4; 0.726027; 0.978261; 0.086396;

Test 4; 0.726027; 0.983696; 0.086288; Test 4; 0.735160; 0.983696; 0.084864; Test 4; 0.735160; 0.989130; 0.084107; Test 4; 0.853881; 0.989130; 0.047592; Test 4; 0.853881; 0.994565; 0.046147; Test 4; 0.876712; 0.994565; 0.039284; Test 4; 0.876712; 1.000000; 0.036216; Test 4; 1.000000; 1.000000; 0.001026; Test 5; 0.000000; 0.000000; 1.997760; Test 5; 0.000000; 0.033333; 0.997760; Test 5; 0.000000; 0.166667; 0.928720;

Test 5; 0.050000; 0.166667; 0.926023; Test 5; 0.050000; 0.200000; 0.921494; Test 5; 0.100000; 0.200000; 0.917027; Test 5; 0.100000; 0.466667; 0.767988; Test 5; 0.150000; 0.466667; 0.760134; Test 5; 0.150000; 0.733333; 0.295431; Test 5; 0.350000; 0.733333; 0.275327; Test 5; 0.350000; 0.766667; 0.269164; Test 5; 0.450000; 0.766667; 0.219247; Test 5; 0.450000; 0.800000; 0.208416; Test 5; 0.550000; 0.800000; 0.200690;

Test 5; 0.550000; 0.866667; 0.162464; Test 5; 0.650000; 0.866667; 0.154124; Test 5; 0.650000; 0.966667; 0.129157; Test 5; 0.950000; 0.966667; 0.015653; Test 5; 0.950000; 1.000000; 0.014107; Test 5; 1.000000; 1.000000; 0.008589; Test 6; 0.000000; 0.000000; 1.950075; Test 6; 0.050000; 0.000000; 0.950075; Test 6; 0.050000; 0.050000; 0.934406; Test 6; 0.150000; 0.050000; 0.816059; Test 6; 0.150000; 0.250000; 0.284937;

Test 6; 0.250000; 0.250000; 0.244699; Test 6; 0.250000; 0.300000; 0.237509; Test 6; 0.400000; 0.300000; 0.182607; Test 6; 0.400000; 0.350000; 0.162707; Test 6; 0.450000; 0.350000; 0.154932; Test 6; 0.450000; 0.450000; 0.138937; Test 6; 0.500000; 0.450000; 0.137762; Test 6; 0.500000; 0.600000; 0.133989; Test 6; 0.550000; 0.600000; 0.133029; Test 6; 0.550000; 0.650000; 0.119355; Test 6; 0.600000; 0.650000; 0.112142;

Test 6; 0.600000; 0.850000; 0.101943; Test 6; 0.850000; 0.850000; 0.067615; Test 6; 0.850000; 0.900000; 0.064231; Test 6; 1.000000; 0.900000; 0.016702; Test 6; 1.000000; 1.000000; 0.001002; Test 7; 0.000000; 0.000000; 1.999723; Test 7; 0.000000; 0.000690; 0.999723; Test 7; 0.000000; 0.030366; 0.989357; Test 7; 0.001130; 0.030366; 0.989114; Test 7; 0.001130; 0.044168; 0.984287; Test 7; 0.002260; 0.044168; 0.984195;

Test 7; 0.002260; 0.052450; 0.982363; Test 7; 0.003390; 0.052450; 0.982331; Test 7; 0.003390; 0.085576; 0.971860; Test 7; 0.004520; 0.085576; 0.971834; Test 7; 0.004520; 0.087647; 0.970814; Test 7; 0.005650; 0.087647; 0.970555; Test 7; 0.005650; 0.089027; 0.970023; Test 7; 0.006780; 0.089027; 0.969762; Test 7; 0.006780; 0.092478; 0.969189; Test 7; 0.007910; 0.092478; 0.968853; Test 7; 0.007910; 0.120773; 0.960541;

Test 7; 0.009040; 0.120773; 0.960533; Test 7; 0.009040; 0.131815; 0.956683; Test 7; 0.010169; 0.131815; 0.955365; Test 7; 0.010169; 0.135266; 0.954601; Test 7; 0.011299; 0.135266; 0.954421; Test 7; 0.011299; 0.142857; 0.951690; Test 7; 0.012429; 0.142857; 0.951628; Test 7; 0.012429; 0.171153; 0.943371; Test 7; 0.013559; 0.171153; 0.943285; Test 7; 0.013559; 0.218772; 0.925766; Test 7; 0.014689; 0.218772; 0.925665;

Test 7; 0.014689; 0.222912; 0.925153; Test 7; 0.015819; 0.222912; 0.925140; Test 7; 0.015819; 0.234645; 0.922287; Test 7; 0.016949; 0.234645; 0.921910; Test 7; 0.016949; 0.237405; 0.920330; Test 7; 0.018079; 0.237405; 0.920160; Test 7; 0.018079; 0.239476; 0.919878; Test 7; 0.019209; 0.239476; 0.919723; Test 7; 0.019209; 0.249137; 0.917032; Test 7; 0.020339; 0.249137; 0.916228; Test 7; 0.020339; 0.252588; 0.915338;

Test 7; 0.021469; 0.252588; 0.915149; Test 7; 0.021469; 0.270531; 0.909422; Test 7; 0.022599; 0.270531; 0.909331; Test 7; 0.022599; 0.273292; 0.908377; Test 7; 0.024859; 0.273292; 0.908335; Test 7; 0.024859; 0.282264; 0.905717; Test 7; 0.025989; 0.282264; 0.905420; Test 7; 0.025989; 0.289855; 0.902714; Test 7; 0.027119; 0.289855; 0.902703; Test 7; 0.027119; 0.300897; 0.899185; Test 7; 0.028249; 0.300897; 0.899080;

Test 7; 0.028249; 0.325742; 0.892112; Test 7; 0.029379; 0.325742; 0.891724; Test 7; 0.029379; 0.333333; 0.889890; Test 7; 0.030508; 0.333333; 0.889472; Test 7; 0.030508; 0.340235; 0.886465; Test 7; 0.031638; 0.340235; 0.886357; Test 7; 0.031638; 0.344375; 0.884891; Test 7; 0.032768; 0.344375; 0.884093; Test 7; 0.032768; 0.356108; 0.879397; Test 7; 0.035028; 0.356108; 0.879329; Test 7; 0.035028; 0.359558; 0.878471;

Test 7; 0.036158; 0.359558; 0.878402; Test 7; 0.036158; 0.366460; 0.876209; Test 7; 0.037288; 0.366460; 0.876192; Test 7; 0.037288; 0.387854; 0.870191; Test 7; 0.038418; 0.387854; 0.870016; Test 7; 0.038418; 0.392685; 0.868410; Test 7; 0.039548; 0.392685; 0.868251; Test 7; 0.039548; 0.400276; 0.866108; Test 7; 0.040678; 0.400276; 0.866078; Test 7; 0.040678; 0.406487; 0.864899; Test 7; 0.041808; 0.406487; 0.864094;

Test 7; 0.041808; 0.418219; 0.860869; Test 7; 0.042938; 0.418219; 0.860689; Test 7; 0.042938; 0.423741; 0.858355; Test 7; 0.044068; 0.423741; 0.858105; Test 7; 0.044068; 0.429262; 0.856748; Test 7; 0.045198; 0.429262; 0.856634; Test 7; 0.045198; 0.442374; 0.850471; Test 7; 0.046328; 0.442374; 0.850118; Test 7; 0.046328; 0.465839; 0.842008; Test 7; 0.047458; 0.465839; 0.841989; Test 7; 0.047458; 0.469979; 0.840552;

Test 7; 0.048588; 0.469979; 0.839965; Test 7; 0.048588; 0.476881; 0.837547; Test 7; 0.049718; 0.476881; 0.836345; Test 7; 0.049718; 0.481021; 0.835121; Test 7; 0.050847; 0.481021; 0.834511; Test 7; 0.050847; 0.485852; 0.833397; Test 7; 0.051977; 0.485852; 0.832671; Test 7; 0.051977; 0.486542; 0.832540; Test 7; 0.053107; 0.486542; 0.832487; Test 7; 0.053107; 0.498275; 0.829871; Test 7; 0.054237; 0.498275; 0.829818;

Test 7; 0.054237; 0.502415; 0.828440; Test 7; 0.055367; 0.502415; 0.828418; Test 7; 0.055367; 0.514838; 0.824302; Test 7; 0.056497; 0.514838; 0.824144; Test 7; 0.056497; 0.521049; 0.822215; Test 7; 0.057627; 0.521049; 0.821037; Test 7; 0.057627; 0.581090; 0.803897; Test 7; 0.058757; 0.581090; 0.803785; Test 7; 0.058757; 0.581781; 0.803660; Test 7; 0.059887; 0.581781; 0.803472; Test 7; 0.059887; 0.657695; 0.774860;

Test 7; 0.061017; 0.657695; 0.774838; Test 7; 0.061017; 0.668047; 0.770914; Test 7; 0.062147; 0.668047; 0.770714; Test 7; 0.062147; 0.688751; 0.764233; Test 7; 0.063277; 0.688751; 0.764055; Test 7; 0.063277; 0.690821; 0.763415; Test 7; 0.064407; 0.690821; 0.762613; Test 7; 0.064407; 0.696342; 0.760699; Test 7; 0.065537; 0.696342; 0.760458; Test 7; 0.065537; 0.701863; 0.758593; Test 7; 0.066667; 0.701863; 0.758543;

Test 7; 0.066667; 0.766736; 0.739948; Test 7; 0.067797; 0.766736; 0.739889; Test 7; 0.067797; 0.768806; 0.739496; Test 7; 0.068927; 0.768806; 0.739347; Test 7; 0.068927; 0.797792; 0.730473; Test 7; 0.070056; 0.797792; 0.730361; Test 7; 0.070056; 0.802622; 0.729365; Test 7; 0.071186; 0.802622; 0.729301; Test 7; 0.071186; 0.819186; 0.724623; Test 7; 0.072316; 0.819186; 0.724370; Test 7; 0.072316; 0.822636; 0.723389;

Test 7; 0.073446; 0.822636; 0.723334; Test 7; 0.073446; 0.854382; 0.712491; Test 7; 0.074576; 0.854382; 0.712452; Test 7; 0.074576; 0.896480; 0.700177; Test 7; 0.088136; 0.896480; 0.296695; Test 7; 0.088136; 0.897170; 0.296567; Test 7; 0.089266; 0.897170; 0.295764; Test 7; 0.089266; 0.897861; 0.295217; Test 7; 0.090395; 0.897861; 0.294945; Test 7; 0.090395; 0.898551; 0.294730; Test 7; 0.094915; 0.898551; 0.294357;

Test 7; 0.094915; 0.899241; 0.293774; Test 7; 0.105085; 0.899241; 0.288910; Test 7; 0.105085; 0.899931; 0.288703; Test 7; 0.108475; 0.899931; 0.288607; Test 7; 0.108475; 0.901311; 0.286910; Test 7; 0.110734; 0.901311; 0.286861; Test 7; 0.110734; 0.902692; 0.286174; Test 7; 0.112994; 0.902692; 0.285785; Test 7; 0.112994; 0.903382; 0.285754; Test 7; 0.115254; 0.903382; 0.285329; Test 7; 0.115254; 0.904072; 0.285014;

Test 7; 0.120904; 0.904072; 0.282187; Test 7; 0.120904; 0.904762; 0.281835; Test 7; 0.125424; 0.904762; 0.280723; Test 7; 0.125424; 0.905452; 0.280508; Test 7; 0.150282; 0.905452; 0.274804; Test 7; 0.150282; 0.906142; 0.273270; Test 7; 0.151412; 0.906142; 0.273214; Test 7; 0.151412; 0.906832; 0.272991; Test 7; 0.159322; 0.906832; 0.270742; Test 7; 0.159322; 0.907522; 0.269985; Test 7; 0.181921; 0.907522; 0.263021;

Test 7; 0.181921; 0.908213; 0.262977; Test 7; 0.185311; 0.908213; 0.262219; Test 7; 0.185311; 0.908903; 0.261898; Test 7; 0.215819; 0.908903; 0.251627; Test 7; 0.215819; 0.909593; 0.251385; Test 7; 0.225989; 0.909593; 0.248845; Test 7; 0.225989; 0.910283; 0.248734; Test 7; 0.232768; 0.910283; 0.247804; Test 7; 0.232768; 0.910973; 0.247758; Test 7; 0.235028; 0.910973; 0.247568; Test 7; 0.235028; 0.911663; 0.247448;

Test 7; 0.237288; 0.911663; 0.247020; Test 7; 0.237288; 0.912353; 0.246921; Test 7; 0.242938; 0.912353; 0.246252; Test 7; 0.242938; 0.913043; 0.245807; Test 7; 0.244068; 0.913043; 0.245648; Test 7; 0.244068; 0.913734; 0.244836; Test 7; 0.246328; 0.913734; 0.244597; Test 7; 0.246328; 0.914424; 0.243524; Test 7; 0.251977; 0.914424; 0.242885; Test 7; 0.251977; 0.915804; 0.242839; Test 7; 0.262147; 0.915804; 0.239908;

Test 7; 0.262147; 0.916494; 0.239439; Test 7; 0.265537; 0.916494; 0.238994; Test 7; 0.265537; 0.917184; 0.237742; Test 7; 0.268927; 0.917184; 0.237154; Test 7; 0.268927; 0.917874; 0.236190; Test 7; 0.275706; 0.917874; 0.235119; Test 7; 0.275706; 0.918565; 0.234784; Test 7; 0.280226; 0.918565; 0.233618; Test 7; 0.280226; 0.919945; 0.233111; Test 7; 0.283616; 0.919945; 0.232447; Test 7; 0.283616; 0.920635; 0.232412;

Test 7; 0.284746; 0.920635; 0.232207; Test 7; 0.284746; 0.921325; 0.232193; Test 7; 0.289266; 0.921325; 0.231654; Test 7; 0.289266; 0.922015; 0.231518; Test 7; 0.292655; 0.922015; 0.231052; Test 7; 0.292655; 0.922705; 0.230920; Test 7; 0.307345; 0.922705; 0.224726; Test 7; 0.307345; 0.924086; 0.224402; Test 7; 0.315254; 0.924086; 0.222852; Test 7; 0.315254; 0.924776; 0.221896; Test 7; 0.319774; 0.924776; 0.220405;

Test 7; 0.319774; 0.925466; 0.220111; Test 7; 0.322034; 0.925466; 0.220030; Test 7; 0.322034; 0.926156; 0.219695; Test 7; 0.324294; 0.926156; 0.219119; Test 7; 0.324294; 0.926846; 0.218946; Test 7; 0.325424; 0.926846; 0.218933; Test 7; 0.325424; 0.928226; 0.218820; Test 7; 0.326554; 0.928226; 0.218523; Test 7; 0.326554; 0.928916; 0.218412; Test 7; 0.346893; 0.928916; 0.211234; Test 7; 0.346893; 0.929607; 0.211145;

Test 7; 0.348023; 0.929607; 0.211064; Test 7; 0.348023; 0.930297; 0.210759; Test 7; 0.349153; 0.930297; 0.210046; Test 7; 0.349153; 0.930987; 0.209773; Test 7; 0.351412; 0.930987; 0.209214; Test 7; 0.351412; 0.931677; 0.209043; Test 7; 0.352542; 0.931677; 0.209032; Test 7; 0.352542; 0.932367; 0.208272; Test 7; 0.358192; 0.932367; 0.206411; Test 7; 0.358192; 0.933057; 0.206373; Test 7; 0.361582; 0.933057; 0.205669;

Test 7; 0.361582; 0.934438; 0.204446; Test 7; 0.364972; 0.934438; 0.202015; Test 7; 0.364972; 0.935128; 0.201902; Test 7; 0.369492; 0.935128; 0.200497; Test 7; 0.369492; 0.935818; 0.200408; Test 7; 0.371751; 0.935818; 0.199113; Test 7; 0.371751; 0.936508; 0.198618; Test 7; 0.372881; 0.936508; 0.198584; Test 7; 0.372881; 0.937198; 0.198067; Test 7; 0.379661; 0.937198; 0.195756; Test 7; 0.379661; 0.938578; 0.195434;

Test 7; 0.387571; 0.938578; 0.194173; Test 7; 0.387571; 0.939268; 0.194027; Test 7; 0.409040; 0.939268; 0.188127; Test 7; 0.409040; 0.940649; 0.187742; Test 7; 0.423729; 0.940649; 0.183571; Test 7; 0.423729; 0.941339; 0.183444; Test 7; 0.428249; 0.941339; 0.182638; Test 7; 0.428249; 0.942029; 0.182612; Test 7; 0.447458; 0.942029; 0.175513; Test 7; 0.447458; 0.942719; 0.174941; Test 7; 0.450847; 0.942719; 0.174248;

Test 7; 0.450847; 0.943409; 0.174115; Test 7; 0.451977; 0.943409; 0.173955; Test 7; 0.451977; 0.944099; 0.173679; Test 7; 0.465537; 0.944099; 0.168842; Test 7; 0.465537; 0.944790; 0.167970; Test 7; 0.476836; 0.944790; 0.165007; Test 7; 0.476836; 0.945480; 0.164898; Test 7; 0.499435; 0.945480; 0.158321; Test 7; 0.499435; 0.946170; 0.158184; Test 7; 0.502825; 0.946170; 0.158018; Test 7; 0.502825; 0.947550; 0.157559;

Test 7; 0.510734; 0.947550; 0.154473; Test 7; 0.510734; 0.948240; 0.154403; Test 7; 0.512994; 0.948240; 0.153365; Test 7; 0.512994; 0.948930; 0.152540; Test 7; 0.520904; 0.948930; 0.148656; Test 7; 0.520904; 0.949620; 0.148203; Test 7; 0.525424; 0.949620; 0.147232; Test 7; 0.525424; 0.950311; 0.146858; Test 7; 0.531073; 0.950311; 0.145608; Test 7; 0.531073; 0.951001; 0.144777; Test 7; 0.536723; 0.951001; 0.142896;

Test 7; 0.536723; 0.951691; 0.142663; Test 7; 0.551412; 0.951691; 0.139547; Test 7; 0.551412; 0.952381; 0.139335; Test 7; 0.554802; 0.952381; 0.138386; Test 7; 0.554802; 0.953071; 0.138229; Test 7; 0.555932; 0.953071; 0.137742; Test 7; 0.555932; 0.953761; 0.137662; Test 7; 0.557062; 0.953761; 0.137483; Test 7; 0.557062; 0.954451; 0.137272; Test 7; 0.558192; 0.954451; 0.137162; Test 7; 0.558192; 0.955141; 0.137059;

Test 7; 0.570621; 0.955141; 0.134172; Test 7; 0.570621; 0.955832; 0.134026; Test 7; 0.571751; 0.955832; 0.133516; Test 7; 0.571751; 0.957212; 0.132898; Test 7; 0.574011; 0.957212; 0.132289; Test 7; 0.574011; 0.959972; 0.131364; Test 7; 0.578531; 0.959972; 0.130394; Test 7; 0.578531; 0.960663; 0.130157; Test 7; 0.580791; 0.960663; 0.130019; Test 7; 0.580791; 0.961353; 0.129895; Test 7; 0.594350; 0.961353; 0.126130;

Test 7; 0.594350; 0.962043; 0.125744; Test 7; 0.595480; 0.962043; 0.125616; Test 7; 0.595480; 0.962733; 0.125431; Test 7; 0.598870; 0.962733; 0.124571; Test 7; 0.598870; 0.963423; 0.124380; Test 7; 0.600000; 0.963423; 0.124251; Test 7; 0.600000; 0.964113; 0.123627; Test 7; 0.605650; 0.964113; 0.120983; Test 7; 0.605650; 0.964803; 0.120584; Test 7; 0.623729; 0.964803; 0.117115; Test 7; 0.623729; 0.965493; 0.116981;

Test 7; 0.653107; 0.965493; 0.109326; Test 7; 0.653107; 0.966184; 0.109072; Test 7; 0.684746; 0.966184; 0.099551; Test 7; 0.684746; 0.966874; 0.098755; Test 7; 0.685876; 0.966874; 0.098440; Test 7; 0.685876; 0.967564; 0.097977; Test 7; 0.697175; 0.967564; 0.093818; Test 7; 0.697175; 0.968254; 0.093695; Test 7; 0.698305; 0.968254; 0.093321; Test 7; 0.698305; 0.968944; 0.093269; Test 7; 0.718644; 0.968944; 0.088752;

Test 7; 0.718644; 0.969634; 0.088201; Test 7; 0.727684; 0.969634; 0.085801; Test 7; 0.727684; 0.970324; 0.085740; Test 7; 0.738983; 0.970324; 0.080925; Test 7; 0.738983; 0.971014; 0.080872; Test 7; 0.751412; 0.971014; 0.074769; Test 7; 0.751412; 0.971705; 0.074583; Test 7; 0.752542; 0.971705; 0.074554; Test 7; 0.752542; 0.972395; 0.074508; Test 7; 0.757062; 0.972395; 0.073970; Test 7; 0.757062; 0.973085; 0.073838;

Test 7; 0.759322; 0.973085; 0.073556; Test 7; 0.759322; 0.973775; 0.073426; Test 7; 0.769492; 0.973775; 0.071520; Test 7; 0.769492; 0.974465; 0.071330; Test 7; 0.771751; 0.974465; 0.070926; Test 7; 0.771751; 0.975155; 0.070757; Test 7; 0.780791; 0.975155; 0.068400; Test 7; 0.780791; 0.975845; 0.068380; Test 7; 0.793220; 0.975845; 0.063176; Test 7; 0.793220; 0.976536; 0.063157; Test 7; 0.795480; 0.976536; 0.062704;

Test 7; 0.795480; 0.977226; 0.062148; Test 7; 0.801130; 0.977226; 0.061656; Test 7; 0.801130; 0.977916; 0.061532; Test 7; 0.805650; 0.977916; 0.060523; Test 7; 0.805650; 0.978606; 0.060307; Test 7; 0.806780; 0.978606; 0.060249; Test 7; 0.806780; 0.979296; 0.060185; Test 7; 0.810169; 0.979296; 0.059568; Test 7; 0.810169; 0.979986; 0.059441; Test 7; 0.813559; 0.979986; 0.058846; Test 7; 0.813559; 0.980676; 0.058190;

Test 7; 0.815819; 0.980676; 0.056931; Test 7; 0.815819; 0.981366; 0.056790; Test 7; 0.818079; 0.981366; 0.056254; Test 7; 0.818079; 0.982747; 0.054991; Test 7; 0.823729; 0.982747; 0.053602; Test 7; 0.823729; 0.983437; 0.052855; Test 7; 0.824859; 0.983437; 0.052679; Test 7; 0.824859; 0.984127; 0.051984; Test 7; 0.829379; 0.984127; 0.051391; Test 7; 0.829379; 0.984817; 0.051307; Test 7; 0.832768; 0.984817; 0.050791;

Test 7; 0.832768; 0.985507; 0.050481; Test 7; 0.836158; 0.985507; 0.049557; Test 7; 0.836158; 0.986197; 0.049465; Test 7; 0.837288; 0.986197; 0.049440; Test 7; 0.837288; 0.986888; 0.049395; Test 7; 0.838418; 0.986888; 0.048921; Test 7; 0.838418; 0.987578; 0.048797; Test 7; 0.844068; 0.987578; 0.046877; Test 7; 0.844068; 0.988268; 0.046752; Test 7; 0.877966; 0.988268; 0.036540; Test 7; 0.877966; 0.988958; 0.036009;

Test 7; 0.880226; 0.988958; 0.034517; Test 7; 0.880226; 0.989648; 0.034303; Test 7; 0.888136; 0.989648; 0.032017; Test 7; 0.888136; 0.990338; 0.031807; Test 7; 0.900565; 0.990338; 0.028489; Test 7; 0.900565; 0.991028; 0.028327; Test 7; 0.911864; 0.991028; 0.024833; Test 7; 0.911864; 0.991718; 0.024176; Test 7; 0.915254; 0.991718; 0.022560; Test 7; 0.915254; 0.992409; 0.022032; Test 7; 0.918644; 0.992409; 0.021659;

Test 7; 0.918644; 0.993099; 0.021466; Test 7; 0.931073; 0.993099; 0.018941; Test 7; 0.931073; 0.993789; 0.018752; Test 7; 0.934463; 0.993789; 0.017628; Test 7; 0.934463; 0.994479; 0.017504; Test 7; 0.955932; 0.994479; 0.013153; Test 7; 0.955932; 0.995169; 0.012949; Test 7; 0.967232; 0.995169; 0.009869; Test 7; 0.967232; 0.995859; 0.009852; Test 7; 0.968362; 0.995859; 0.008926; Test 7; 0.968362; 0.996549; 0.008386;

Test 7; 0.971751; 0.996549; 0.007605; Test 7; 0.971751; 0.997239; 0.007378; Test 7; 0.974011; 0.997239; 0.006964; Test 7; 0.974011; 0.997930; 0.006807; Test 7; 0.990960; 0.997930; 0.003340; Test 7; 0.990960; 0.998620; 0.003041; Test 7; 0.998870; 0.998620; 0.001413; Test 7; 0.998870; 1.000000; 0.001321; Test 7; 1.000000; 1.000000; 0.000104; Test 8; 0.000000; 0.000000; 1.999945; Test 8; 0.001010; 0.000000; 0.999945;

Test 8; 0.001010; 0.002527; 0.999661; Test 8; 0.002020; 0.002527; 0.999630; Test 8; 0.002020; 0.003370; 0.998722; Test 8; 0.003030; 0.003370; 0.997965; Test 8; 0.003030; 0.026116; 0.990634; Test 8; 0.004040; 0.026116; 0.990494; Test 8; 0.004040; 0.027801; 0.989362; Test 8; 0.005051; 0.027801; 0.989189; Test 8; 0.005051; 0.048863; 0.984057; Test 8; 0.006061; 0.048863; 0.983968; Test 8; 0.006061; 0.057287; 0.980723;

Test 8; 0.007071; 0.057287; 0.980316; Test 8; 0.007071; 0.080876; 0.971900; Test 8; 0.008081; 0.080876; 0.971797; Test 8; 0.008081; 0.103623; 0.966695; Test 8; 0.009091; 0.103623; 0.966229; Test 8; 0.009091; 0.122157; 0.960403; Test 8; 0.010101; 0.122157; 0.960222; Test 8; 0.010101; 0.154170; 0.951272; Test 8; 0.011111; 0.154170; 0.950927; Test 8; 0.011111; 0.155013; 0.950863; Test 8; 0.012121; 0.155013; 0.950614;

Test 8; 0.012121; 0.161752; 0.948992; Test 8; 0.013131; 0.161752; 0.948721; Test 8; 0.013131; 0.172704; 0.944407; Test 8; 0.014141; 0.172704; 0.944329; Test 8; 0.014141; 0.180286; 0.941322; Test 8; 0.015152; 0.180286; 0.941233; Test 8; 0.015152; 0.189553; 0.936322; Test 8; 0.016162; 0.189553; 0.936163; Test 8; 0.016162; 0.197978; 0.934207; Test 8; 0.017172; 0.197978; 0.933335; Test 8; 0.017172; 0.206403; 0.930864;

Test 8; 0.018182; 0.206403; 0.930726; Test 8; 0.018182; 0.229992; 0.923623; Test 8; 0.019192; 0.229992; 0.922977; Test 8; 0.019192; 0.235889; 0.921563; Test 8; 0.020202; 0.235889; 0.921441; Test 8; 0.020202; 0.256950; 0.915188; Test 8; 0.021212; 0.256950; 0.914778; Test 8; 0.021212; 0.279697; 0.907718; Test 8; 0.022222; 0.279697; 0.906820; Test 8; 0.022222; 0.319292; 0.894644; Test 8; 0.023232; 0.319292; 0.894327;

Test 8; 0.023232; 0.320135; 0.894250; Test 8; 0.024242; 0.320135; 0.894186; Test 8; 0.024242; 0.320977; 0.893749; Test 8; 0.025253; 0.320977; 0.893476; Test 8; 0.025253; 0.329402; 0.890122; Test 8; 0.026263; 0.329402; 0.890028; Test 8; 0.026263; 0.390059; 0.868798; Test 8; 0.027273; 0.390059; 0.868789; Test 8; 0.027273; 0.390901; 0.868463; Test 8; 0.028283; 0.390901; 0.868444; Test 8; 0.028283; 0.401853; 0.865289;

Test 8; 0.029293; 0.401853; 0.865217; Test 8; 0.029293; 0.417860; 0.861047; Test 8; 0.030303; 0.417860; 0.860928; Test 8; 0.030303; 0.419545; 0.860357; Test 8; 0.031313; 0.419545; 0.859324; Test 8; 0.031313; 0.433867; 0.854278; Test 8; 0.032323; 0.433867; 0.853915; Test 8; 0.032323; 0.439764; 0.852502; Test 8; 0.033333; 0.439764; 0.852248; Test 8; 0.033333; 0.468408; 0.843936; Test 8; 0.034343; 0.468408; 0.843796;

Test 8; 0.034343; 0.483572; 0.839526; Test 8; 0.035354; 0.483572; 0.838585; Test 8; 0.035354; 0.506318; 0.827725; Test 8; 0.036364; 0.506318; 0.827030; Test 8; 0.036364; 0.508846; 0.826945; Test 8; 0.037374; 0.508846; 0.826789; Test 8; 0.037374; 0.513058; 0.825788; Test 8; 0.038384; 0.513058; 0.825199; Test 8; 0.038384; 0.530750; 0.819328; Test 8; 0.039394; 0.530750; 0.819294; Test 8; 0.039394; 0.587195; 0.797933;

Test 8; 0.040404; 0.587195; 0.797625; Test 8; 0.040404; 0.641112; 0.779950; Test 8; 0.041414; 0.641112; 0.779924; Test 8; 0.041414; 0.647852; 0.778905; Test 8; 0.042424; 0.647852; 0.778815; Test 8; 0.042424; 0.650379; 0.778255; Test 8; 0.043434; 0.650379; 0.778102; Test 8; 0.043434; 0.653749; 0.777183; Test 8; 0.044444; 0.653749; 0.776836; Test 8; 0.044444; 0.682393; 0.767904; Test 8; 0.045455; 0.682393; 0.767863;

Test 8; 0.045455; 0.684078; 0.767781; Test 8; 0.046465; 0.684078; 0.767422; Test 8; 0.046465; 0.700084; 0.762248; Test 8; 0.047475; 0.700084; 0.762177; Test 8; 0.047475; 0.700927; 0.761917; Test 8; 0.048485; 0.700927; 0.761580; Test 8; 0.048485; 0.726201; 0.753588; Test 8; 0.049495; 0.726201; 0.753191; Test 8; 0.049495; 0.743050; 0.746558; Test 8; 0.050505; 0.743050; 0.746321; Test 8; 0.050505; 0.743892; 0.746051;

Test 8; 0.051515; 0.743892; 0.745928; Test 8; 0.051515; 0.759056; 0.741593; Test 8; 0.052525; 0.759056; 0.741087; Test 8; 0.052525; 0.782645; 0.732805; Test 8; 0.053535; 0.782645; 0.732602; Test 8; 0.053535; 0.802864; 0.724674; Test 8; 0.054545; 0.802864; 0.724546; Test 8; 0.054545; 0.807077; 0.723274; Test 8; 0.055556; 0.807077; 0.723164; Test 8; 0.055556; 0.818871; 0.721073; Test 8; 0.056566; 0.818871; 0.720744;

Test 8; 0.056566; 0.820556; 0.719751; Test 8; 0.057576; 0.820556; 0.719518; Test 8; 0.057576; 0.844145; 0.712358; Test 8; 0.058586; 0.844145; 0.712050; Test 8; 0.058586; 0.862679; 0.708469; Test 8; 0.060606; 0.862679; 0.708128; Test 8; 0.060606; 0.866049; 0.706605; Test 8; 0.061616; 0.866049; 0.706054; Test 8; 0.061616; 0.874473; 0.704295; Test 8; 0.062626; 0.874473; 0.704279; Test 8; 0.062626; 0.879528; 0.702457;

Test 8; 0.063636; 0.879528; 0.702270; Test 8; 0.063636; 0.883741; 0.700056; Test 8; 0.071717; 0.883741; 0.298484; Test 8; 0.071717; 0.884583; 0.298360; Test 8; 0.075758; 0.884583; 0.297261; Test 8; 0.075758; 0.885425; 0.297188; Test 8; 0.081818; 0.885425; 0.295334; Test 8; 0.081818; 0.886268; 0.295156; Test 8; 0.092929; 0.886268; 0.293342; Test 8; 0.092929; 0.887110; 0.292943; Test 8; 0.094949; 0.887110; 0.292598;

Test 8; 0.094949; 0.887953; 0.292520; Test 8; 0.095960; 0.887953; 0.292316; Test 8; 0.095960; 0.888795; 0.292189; Test 8; 0.097980; 0.888795; 0.290871; Test 8; 0.097980; 0.889638; 0.290797; Test 8; 0.100000; 0.889638; 0.289209; Test 8; 0.100000; 0.890480; 0.288943; Test 8; 0.102020; 0.890480; 0.288454; Test 8; 0.102020; 0.891323; 0.288362; Test 8; 0.118182; 0.891323; 0.284460; Test 8; 0.118182; 0.892165; 0.284230;

Test 8; 0.126263; 0.892165; 0.282862; Test 8; 0.126263; 0.893008; 0.282642; Test 8; 0.127273; 0.893008; 0.281897; Test 8; 0.127273; 0.894693; 0.281657; Test 8; 0.134343; 0.894693; 0.278781; Test 8; 0.134343; 0.895535; 0.278736; Test 8; 0.138384; 0.895535; 0.276839; Test 8; 0.138384; 0.896377; 0.276049; Test 8; 0.140404; 0.896377; 0.276011; Test 8; 0.140404; 0.897220; 0.275632; Test 8; 0.156566; 0.897220; 0.271306;

Test 8; 0.156566; 0.898905; 0.271107; Test 8; 0.163636; 0.898905; 0.269565; Test 8; 0.163636; 0.900590; 0.269047; Test 8; 0.167677; 0.900590; 0.267484; Test 8; 0.167677; 0.901432; 0.267175; Test 8; 0.173737; 0.901432; 0.265159; Test 8; 0.173737; 0.902275; 0.264887; Test 8; 0.175758; 0.902275; 0.264860; Test 8; 0.175758; 0.903117; 0.264555; Test 8; 0.179798; 0.903117; 0.262331; Test 8; 0.179798; 0.903960; 0.262224;

Test 8; 0.182828; 0.903960; 0.261632; Test 8; 0.182828; 0.905644; 0.261038; Test 8; 0.189899; 0.905644; 0.258502; Test 8; 0.189899; 0.906487; 0.258498; Test 8; 0.197980; 0.906487; 0.255920; Test 8; 0.197980; 0.907329; 0.255104; Test 8; 0.198990; 0.907329; 0.254950; Test 8; 0.198990; 0.908172; 0.254658; Test 8; 0.201010; 0.908172; 0.253908; Test 8; 0.201010; 0.909014; 0.253672; Test 8; 0.229293; 0.909014; 0.245614;

Test 8; 0.229293; 0.909857; 0.245601; Test 8; 0.230303; 0.909857; 0.245490; Test 8; 0.230303; 0.910699; 0.245318; Test 8; 0.260606; 0.910699; 0.236290; Test 8; 0.260606; 0.911542; 0.235676; Test 8; 0.287879; 0.911542; 0.226609; Test 8; 0.287879; 0.912384; 0.226549; Test 8; 0.289899; 0.912384; 0.226163; Test 8; 0.289899; 0.913227; 0.226098; Test 8; 0.292929; 0.913227; 0.224879; Test 8; 0.292929; 0.914069; 0.224585;

Test 8; 0.312121; 0.914069; 0.220166; Test 8; 0.312121; 0.914912; 0.220131; Test 8; 0.318182; 0.914912; 0.218643; Test 8; 0.318182; 0.915754; 0.218393; Test 8; 0.321212; 0.915754; 0.217309; Test 8; 0.321212; 0.916596; 0.217117; Test 8; 0.323232; 0.916596; 0.216413; Test 8; 0.323232; 0.917439; 0.216327; Test 8; 0.325253; 0.917439; 0.216171; Test 8; 0.325253; 0.918281; 0.216067; Test 8; 0.332323; 0.918281; 0.214223;

Test 8; 0.332323; 0.919124; 0.213861; Test 8; 0.336364; 0.919124; 0.212729; Test 8; 0.336364; 0.919966; 0.212188; Test 8; 0.342424; 0.919966; 0.211403; Test 8; 0.342424; 0.920809; 0.210428; Test 8; 0.344444; 0.920809; 0.209180; Test 8; 0.344444; 0.921651; 0.208921; Test 8; 0.346465; 0.921651; 0.208406; Test 8; 0.346465; 0.922494; 0.208341; Test 8; 0.355556; 0.922494; 0.204961; Test 8; 0.355556; 0.923336; 0.204940;

Test 8; 0.364646; 0.923336; 0.202339; Test 8; 0.364646; 0.924179; 0.202242; Test 8; 0.373737; 0.924179; 0.199481; Test 8; 0.373737; 0.925021; 0.199411; Test 8; 0.381818; 0.925021; 0.197888; Test 8; 0.381818; 0.925864; 0.197613; Test 8; 0.382828; 0.925864; 0.196919; Test 8; 0.382828; 0.926706; 0.196532; Test 8; 0.383838; 0.926706; 0.196511; Test 8; 0.383838; 0.928391; 0.196246; Test 8; 0.384848; 0.928391; 0.195286;

Test 8; 0.384848; 0.930076; 0.195169; Test 8; 0.392929; 0.930076; 0.193314; Test 8; 0.392929; 0.931761; 0.193056; Test 8; 0.393939; 0.931761; 0.192827; Test 8; 0.393939; 0.932603; 0.192574; Test 8; 0.395960; 0.932603; 0.191441; Test 8; 0.395960; 0.934288; 0.190286; Test 8; 0.396970; 0.934288; 0.190202; Test 8; 0.396970; 0.935131; 0.190158; Test 8; 0.404040; 0.935131; 0.188812; Test 8; 0.404040; 0.935973; 0.188556;

Test 8; 0.408081; 0.935973; 0.187479; Test 8; 0.408081; 0.936816; 0.187463; Test 8; 0.422222; 0.936816; 0.183505; Test 8; 0.422222; 0.937658; 0.183273; Test 8; 0.429293; 0.937658; 0.180632; Test 8; 0.429293; 0.938500; 0.180547; Test 8; 0.454545; 0.938500; 0.172450; Test 8; 0.454545; 0.939343; 0.172371; Test 8; 0.470707; 0.939343; 0.167558; Test 8; 0.470707; 0.940185; 0.167354; Test 8; 0.497980; 0.940185; 0.160210;

Test 8; 0.497980; 0.941028; 0.159985; Test 8; 0.501010; 0.941028; 0.159734; Test 8; 0.501010; 0.941870; 0.159618; Test 8; 0.506061; 0.941870; 0.158498; Test 8; 0.506061; 0.942713; 0.158378; Test 8; 0.535354; 0.942713; 0.152194; Test 8; 0.535354; 0.943555; 0.151877; Test 8; 0.538384; 0.943555; 0.151527; Test 8; 0.538384; 0.944398; 0.150940; Test 8; 0.540404; 0.944398; 0.150349; Test 8; 0.540404; 0.945240; 0.150132;

Test 8; 0.544444; 0.945240; 0.149615; Test 8; 0.544444; 0.946083; 0.149522; Test 8; 0.563636; 0.946083; 0.145080; Test 8; 0.563636; 0.946925; 0.144896; Test 8; 0.567677; 0.946925; 0.143065; Test 8; 0.567677; 0.947767; 0.142742; Test 8; 0.575758; 0.947767; 0.141669; Test 8; 0.575758; 0.948610; 0.141533; Test 8; 0.577778; 0.948610; 0.141102; Test 8; 0.577778; 0.950295; 0.140966; Test 8; 0.581818; 0.950295; 0.138493;

Test 8; 0.581818; 0.951137; 0.138478; Test 8; 0.603030; 0.951137; 0.130355; Test 8; 0.603030; 0.951980; 0.130291; Test 8; 0.613131; 0.951980; 0.125621; Test 8; 0.613131; 0.952822; 0.125203; Test 8; 0.623232; 0.952822; 0.123044; Test 8; 0.623232; 0.953665; 0.123008; Test 8; 0.624242; 0.953665; 0.122970; Test 8; 0.624242; 0.954507; 0.122876; Test 8; 0.630303; 0.954507; 0.120984; Test 8; 0.630303; 0.955350; 0.120953;

Test 8; 0.639394; 0.955350; 0.118211; Test 8; 0.639394; 0.957035; 0.117547; Test 8; 0.645455; 0.957035; 0.114848; Test 8; 0.645455; 0.957877; 0.114816; Test 8; 0.647475; 0.957877; 0.114232; Test 8; 0.647475; 0.958719; 0.114212; Test 8; 0.657576; 0.958719; 0.111332; Test 8; 0.657576; 0.959562; 0.111187; Test 8; 0.664646; 0.959562; 0.108456; Test 8; 0.664646; 0.960404; 0.108116; Test 8; 0.667677; 0.960404; 0.106634;

Test 8; 0.667677; 0.961247; 0.106609; Test 8; 0.674747; 0.961247; 0.105532; Test 8; 0.674747; 0.962089; 0.105500; Test 8; 0.676768; 0.962089; 0.104660; Test 8; 0.676768; 0.962932; 0.104580; Test 8; 0.687879; 0.962932; 0.101967; Test 8; 0.687879; 0.963774; 0.101922; Test 8; 0.688889; 0.963774; 0.101819; Test 8; 0.688889; 0.964617; 0.101651; Test 8; 0.690909; 0.964617; 0.101046; Test 8; 0.690909; 0.966302; 0.100940;

Test 8; 0.700000; 0.966302; 0.099337; Test 8; 0.700000; 0.967144; 0.099173; Test 8; 0.706061; 0.967144; 0.096959; Test 8; 0.706061; 0.967987; 0.096292; Test 8; 0.709091; 0.967987; 0.095306; Test 8; 0.709091; 0.968829; 0.095108; Test 8; 0.710101; 0.968829; 0.094714; Test 8; 0.710101; 0.969671; 0.094706; Test 8; 0.723232; 0.969671; 0.092075; Test 8; 0.723232; 0.971356; 0.091284; Test 8; 0.750505; 0.971356; 0.083175;

Test 8; 0.750505; 0.972199; 0.082781; Test 8; 0.779798; 0.972199; 0.072943; Test 8; 0.779798; 0.973041; 0.072830; Test 8; 0.788889; 0.973041; 0.070585; Test 8; 0.788889; 0.973884; 0.069430; Test 8; 0.789899; 0.973884; 0.068996; Test 8; 0.789899; 0.974726; 0.068756; Test 8; 0.794949; 0.974726; 0.067215; Test 8; 0.794949; 0.975569; 0.066886; Test 8; 0.805051; 0.975569; 0.064714; Test 8; 0.805051; 0.976411; 0.064580;

Test 8; 0.806061; 0.976411; 0.064362; Test 8; 0.806061; 0.977254; 0.063849; Test 8; 0.811111; 0.977254; 0.062734; Test 8; 0.811111; 0.978096; 0.062489; Test 8; 0.815152; 0.978096; 0.060862; Test 8; 0.815152; 0.978939; 0.060464; Test 8; 0.821212; 0.978939; 0.059019; Test 8; 0.821212; 0.979781; 0.058977; Test 8; 0.838384; 0.979781; 0.054238; Test 8; 0.838384; 0.980623; 0.053843; Test 8; 0.846465; 0.980623; 0.052753;

Test 8; 0.846465; 0.981466; 0.052705; Test 8; 0.848485; 0.981466; 0.052514; Test 8; 0.848485; 0.982308; 0.051617; Test 8; 0.851515; 0.982308; 0.051003; Test 8; 0.851515; 0.983151; 0.050886; Test 8; 0.860606; 0.983151; 0.048945; Test 8; 0.860606; 0.984836; 0.048333; Test 8; 0.863636; 0.984836; 0.047157; Test 8; 0.863636; 0.985678; 0.047032; Test 8; 0.871717; 0.985678; 0.044653; Test 8; 0.871717; 0.986521; 0.043774;

Test 8; 0.874747; 0.986521; 0.042625; Test 8; 0.874747; 0.987363; 0.042243; Test 8; 0.878788; 0.987363; 0.041681; Test 8; 0.878788; 0.988206; 0.040797; Test 8; 0.884848; 0.988206; 0.039011; Test 8; 0.884848; 0.989890; 0.038076; Test 8; 0.890909; 0.989890; 0.034545; Test 8; 0.890909; 0.990733; 0.034249; Test 8; 0.894949; 0.990733; 0.033146; Test 8; 0.894949; 0.991575; 0.032875; Test 8; 0.896970; 0.991575; 0.032525;

Test 8; 0.896970; 0.992418; 0.031648; Test 8; 0.897980; 0.992418; 0.031576; Test 8; 0.897980; 0.993260; 0.031461; Test 8; 0.908081; 0.993260; 0.028225; Test 8; 0.908081; 0.994103; 0.028113; Test 8; 0.909091; 0.994103; 0.027149; Test 8; 0.909091; 0.994945; 0.027120; Test 8; 0.926263; 0.994945; 0.021288; Test 8; 0.926263; 0.995788; 0.021068; Test 8; 0.933333; 0.995788; 0.019362; Test 8; 0.933333; 0.996630; 0.019296;

Test 8; 0.942424; 0.996630; 0.017090; Test 8; 0.942424; 0.997473; 0.016040; Test 8; 0.981818; 0.997473; 0.006377; Test 8; 0.981818; 0.998315; 0.005934; Test 8; 0.994949; 0.998315; 0.002338; Test 8; 0.994949; 0.999158; 0.002324; Test 8; 0.998990; 0.999158; 0.001598; Test 8; 0.998990; 1.000000; 0.001357; Test 8; 1.000000; 1.000000; 0.000849

S10. PRISMA Checklist

Section and Topic	Item #	Checklist item	Location where item is reported
TITLE			
Title	1	Identify the report as a systematic review.	Yes, Page 1
ABSTRACT			
Abstract	2	See the PRISMA 2020 for Abstracts checklist.	Yes, Page 1
INTRODUCTION			
Rationale	3	Describe the rationale for the review in the context of existing knowledge.	Yes, Page 1-2
Objectives	4	Provide an explicit statement of the objective(s) or question(s) the review addresses.	Yes, Page 1-2
METHODS			
Eligibility criteria	5	Specify the inclusion and exclusion criteria for the review and how studies were grouped for the syntheses.	Yes, Page 2-4
Information sources	6	Specify all databases, registers, websites, organisations, reference lists and other sources searched or consulted to identify studies. Specify the date when each source was last searched or consulted.	Yes, Page 2-4
Search strategy	7	Present the full search strategies for all databases, registers and websites, including any filters and limits used.	Yes, Page 2-4
Selection process	8	Specify the methods used to decide whether a study met the inclusion criteria of the review, including how many reviewers screened each record and each report retrieved, whether they worked independently, and if applicable, details of automation tools used in the process.	Yes, Page 2-4
Data collection process	9	Specify the methods used to collect data from reports, including how many reviewers collected data from each report, whether they worked independently, any processes for obtaining or confirming data from study investigators, and if applicable, details of automation tools used in the process.	Yes, Page 2-4
Data items	10a	List and define all outcomes for which data were sought. Specify whether all results that were compatible with each outcome domain in each study were sought (e.g. for all measures, time points, analyses), and if not, the methods used to decide which results to collect.	Yes, Page 2-4
	10b	List and define all other variables for which data were sought (e.g. participant and intervention characteristics, funding sources). Describe any assumptions made about any missing or unclear information.	Yes, Page 2-4
Study risk of bias assessment	11	Specify the methods used to assess risk of bias in the included studies, including details of the tool(s) used, how many reviewers assessed each study and whether they worked independently, and if applicable, details of automation tools used in the process.	Yes, Page 2-4
Effect measures	12	Specify for each outcome the effect measure(s) (e.g. risk ratio, mean difference) used in the synthesis or presentation of results.	Yes, Page 2-4
Synthesis methods	13a	Describe the processes used to decide which studies were eligible for each synthesis (e.g. tabulating the study intervention characteristics and comparing against the planned groups for each synthesis (item #5)).	Yes, Page 2-4
	13b	Describe any methods required to prepare the data for presentation or synthesis, such as handling of missing summary statistics, or data conversions.	Yes, Page 2-4
	13c	Describe any methods used to tabulate or visually display results of individual studies and syntheses.	Yes, Page 2-4
	13d	Describe any methods used to synthesize results and provide a rationale for the choice(s). If meta-analysis was performed, describe the model(s), method(s) to identify the presence and extent of statistical heterogeneity, and software package(s) used.	Yes, Page 2-4
	13e	Describe any methods used to explore possible causes of heterogeneity among study results (e.g. subgroup analysis, meta-regression).	Yes, Page 2-4
	13f	Describe any sensitivity analyses conducted to assess robustness of the synthesized results.	Yes, Page 2-4
Reporting bias assessment	14	Describe any methods used to assess risk of bias due to missing results in a synthesis (arising from reporting biases).	Yes, Page 2-4
Certainty assessment	15	Describe any methods used to assess certainty (or confidence) in the body of evidence for an outcome.	Yes, Page 2-4
RESULTS			

Study selection	16a	Describe the results of the search and selection process, from the number of records identified in the search to the number of studies included in the review, ideally using a flow diagram.	Yes, Page 4-17
	16b	Cite studies that might appear to meet the inclusion criteria, but which were excluded, and explain why they were excluded.	Yes, Page 4-17
Study characteristics	17	Cite each included study and present its characteristics.	Yes, Page 4-17
Risk of bias in studies	18	Present assessments of risk of bias for each included study.	Yes, Page 4-17
Results of individual studies	19	For all outcomes, present, for each study: (a) summary statistics for each group (where appropriate) and (b) an effect estimate and its precision (e.g. confidence/credible interval), ideally using structured tables or plots.	Yes, Page 4-17
Results of syntheses	20a	For each synthesis, briefly summarise the characteristics and risk of bias among contributing studies.	Yes, Page 4-17
	20b	Present results of all statistical syntheses conducted. If meta-analysis was done, present for each the summary estimate and its precision (e.g. confidence/credible interval) and measures of statistical heterogeneity. If comparing groups, describe the direction of the effect.	Yes, Page 4-17
	20c	Present results of all investigations of possible causes of heterogeneity among study results.	Yes, Page 4-17
	20d	Present results of all sensitivity analyses conducted to assess the robustness of the synthesized results.	Yes, Page 4-17
Reporting biases	21	Present assessments of risk of bias due to missing results (arising from reporting biases) for each synthesis assessed.	Yes, Page 4-17
Certainty of evidence	22	Present assessments of certainty (or confidence) in the body of evidence for each outcome assessed.	Yes, Page 4-17
DISCUSSION			
Discussion	23a	Provide a general interpretation of the results in the context of other evidence.	Yes, Page 17- 22
	23b	Discuss any limitations of the evidence included in the review.	Yes, Page 17- 22
	23c	Discuss any limitations of the review processes used.	Yes, Page 17- 22
	23d	Discuss implications of the results for practice, policy, and future research.	Yes, Page 17- 22
OTHER INFORMATION			
Registration and protocol	24a	Provide registration information for the review, including register name and registration number, or state that the review was not registered.	Yes, Page 3
	24b	Indicate where the review protocol can be accessed, or state that a protocol was not prepared.	Yes, Page 3
	24c	Describe and explain any amendments to information provided at registration or in the protocol.	Yes, Page 3
Support	25	Describe sources of financial or non-financial support for the review, and the role of the funders or sponsors in the review.	Yes, Page 23
Competing interests	26	Declare any competing interests of review authors.	Yes, Page 23
Availability of data, code and other materials	27	Report which of the following are publicly available and where they can be found: template data collection forms; data extracted from included studies; data used for all analyses; analytic code; any other materials used in the review.	Yes, Page 23 (Supplementary material)

From: Page MJ, McKenzie JE, Bossuyt PM, Boutron I, Hoffmann TC, Mulrow CD, et al. The PRISMA 2020 statement: an updated guideline for reporting systematic reviews. BMJ 2021;372:n71. doi: 10.1136/bmj.n71. This work is licensed under CC BY 4.0. To view a copy of this license, visit https://creativecommons.org/licenses/by/4.0/
